# Resolving human α *versus* β cell fate allocation for the generation of stem cell-derived islets

**DOI:** 10.1038/s41467-026-75255-7

**Published:** 2026-07-09

**Authors:** Melis Akgün Canan, Corinna Cozzitorto, Michael Sterr, Lama Saber, Eunike S. A. Setyono, Alessandro Dema, Kei Kozawa, Xianming Wang, Juliane Merl-Pham, Tobias Greisle, Ingo Burtscher, Heiko Lickert

**Affiliations:** 1 Institute of Diabetes and Regeneration Research, Helmholtz Center Munich, Neuherberg, Germany; 2 School of Medicine, Technical University of Munich, Munich, Germany; 3 Stem Cell Institute, Ankara University, Ankara, Türkiye; 4 Integrated Technologies Research Center (BÜTAM), Ankara University, Ankara, Türkiye; 5 German Center for Diabetes Research (DZD), Neuherberg, Germany; 6 Metabolomics and Proteomics Core, Helmholtz Center Munich, Munich, Germany

**Keywords:** Stem-cell differentiation, Differentiation, Diabetes

## Abstract

Stem cell-derived glucagon-(α) and insulin-producing (β) cells allow to engineer in vitro biomimetics of islet of Langerhans, the micro-organ controlling glycemia; however, a knowledge gap in the mechanism by which human stem cell-derived α and β cells are specified persists. Mouse studies postulated that Aristaless Related homeobox (Arx) and Paired box 4 (Pax4) transcription factors cross-inhibit each other in endocrine progenitors to promote α/β fate allocation, respectively. To test this model in human, we combine lineage labelling with single-cell multiomic analysis in our newly generated *ARX*^*CFP/CFP*^; *PAX4*^*mCherry/mCherry*^ knock-in induced pluripotent stem cell reporter line. Lineage tracing, proteomic and gene regulatory network analysis and potency assays reveal a human specific regulation of α/β cell fate allocation. Pharmacological perturbations previously proposed to trigger α-to-β transdifferentiation or identified by our gene regulatory network lead to enhanced endocrine induction and directed α/β cell fate. Studying mechanisms of endocrinogenesis and fate segregation enables the engineering of islets in vitro, and has broader implications for cell-replacement therapy, disease modelling and drug screening.

## Abstract (web summary)

The human-specific mechanism governing pancreatic α/β cell fate determination via the transcription factors ARX and PAX4 remains poorly defined. Lineage tracing of stem cell-derived islets supports a new model of these factors’ interaction.

## Introduction

Diabetes is a metabolic disease characterized by impaired blood glucose homoeostasis due to insufficient insulin levels. Causes are immune-dependent destruction of insulin-producing β cells (type 1 diabetes – T1D) or β cell dysfunction (type 2 diabetes – T2D)^1,2^. Depending on the species, β cells make up between 60% (human and non-human primates) and 80% (mouse) of the hormone-producing endocrine compartment of the pancreas, the islet of Langerhans. Other cellular components of pancreatic islets are glucagon-producing α cells, somatostatin-producing δ cells, together with ε and PP cells producing ghrelin and pancreatic polypeptide, respectively^3–5^.

Islet transplantation is an effective treatment to reach independence from exogenous insulin, but availability of cadaveric islets from human donors is scarce^6^. Within the search for possible replacements for lost or dysfunctional β cells, efforts over the years focused on the establishment of differentiation protocols that could provide an unlimited source of stem cell-derived β cells (SC-β cells) for cell therapy^7,8^. Stepwise differentiation protocols mimic pancreatic development, progressing through anterior definitive endoderm (ADE), primitive gut tube (PGT), pancreatic progenitor (PP), and endocrine progenitor (EP) stages to guide the differentiation of hormone-producing cells in vitro starting from diverse human pluripotent stem cell (PSC) sources^9–18^. These protocols not only heavily rely on the knowledge acquired from studying mouse pancreas development, but also use empirically tested chemicals to improve certain stages of differentiation, resulting in cell products (SC-islets) that do not completely resemble either human or mouse islets. In addition to generating heterogeneous SC-islets comprising undesired cell types, state-of-the-art protocols progressively lose differentiation efficiency starting from the induction of EPs to the differentiation of SC-β cells^8,19^.

To improve the differentiation efficiency towards either SC-α or β cells, a better understanding of the mechanisms of endocrine cell specification during human pancreas development is needed. In the mouse, endocrine cell fate decision towards α or β cells takes place when EPs expressing the endocrine master regulator *Neurogenin 3* (*Neurog3, Ngn3*) start to express the transcription factors (TFs) *Aristaless Related homeobox* (*Arx*) or *Paired Box 4* (*Pax4*), respectively^20,21^. *Arx* knock-out (KO) mice have an increased number of β cells with a near-total loss of α cells, whereas KO mice for *Pax4* have an increased number of α cells and virtually no β cells^20,22^. In addition, double KO mice for *Arx* and *Pax4* show a virtual total loss of both α and β cells, with increased numbers of δ and PP cells^23^. These genetic studies support a model of α *versus* β cell fate decision in which Arx and Pax4 stochastically compete with each other to promote α or β cell differentiation^21,23^. However, many questions remain open, i.e., (1) How would this direct cross-regulation be achieved mechanistically? (2) What are the upstream regulators of both *ARX* and *PAX4* transcription, and consequently α vs β cell fate allocation? (3) Is this mouse-based model conserved during evolution and in human pancreas development? All these aspects have never been tested due to the lack of available tools to directly assess ARX and PAX4 activity and potency. As of the preparation of this manuscript, three protocols have been established to direct pluripotent stem cell differentiation toward the α cell fate^24–26^ and two studies described the human PSC reporter lines for α and β cells^27,28^, but no human model has been established to specifically follow spatio-temporally ARX and PAX4 activity to decipher mechanistically how α vs β cell fate allocation occurs.

Studies in mouse models proposed α-to-β cell fate conversion from residual or supernumerary α cells as a potential mechanism for β cell regeneration in T1D^29–31^. In the search for potential drugs promoting this mechanism, one study showed artemether as a trigger for α-to-β cell transdifferentiation both in vitro and in vivo^32^. Since then, the effect of the antimalarial drugs Artemisinins on *Arx* and *Pax4* expression and, more in general, on pancreatic cell fate decision and regeneration is controversial. Some studies reported their impact on Arx and Pax4 expression and/or function, endocrine cell fate commitment and regeneration of β cells^32–34^, while other authors reported opposite results or no changes upon treatment^35–37^. It is important to notice that while these studies explored the effect of Artemisinins in multiple species and experimental settings, to the best of our knowledge, none of them interrogated the effect of this class of antimalarial drugs during human pancreatic endocrine development.

Here, we generated a hiPSC fluorescent double reporter line to follow ARX and PAX4 dynamics. We first used this tool to study the dichotomy between the two TFs. We used multiomic and proteomic analysis together with cell potency assays to generate a human gene regulatory network (GRN) involving both *ARX* and *PAX4* and propose a revised model of human α *versus* β cell fate decision. We then used the same cell line to test the effect of drugs that have been identified by our GRN analysis or proposed as triggers of α-to-β cell transdifferentiation on α *versus* β cell fate decision. In particular, we showed that treatment with a small-molecule inhibitor of RE1 Silencing Transcription Factor (REST) increases the number of PAX4^+^ cells and consequently SC-β cells. Treatment with artemether during human SC-islets differentiation promotes late PP expansion, endocrine induction, and increases β cell differentiation at the expense of α cells. With the generation of a double ARX/PAX4 hiPSC reporter line, our results shed light on the mode of human endocrinogenesis and pave the way for drug testing to improve differentiation protocols to generate SC-islets with a defined ratio of SC-α and β cells to mimic the islet of Langerhans.

## Results

### PAX4 and ARX are not co-expressed in early human NEUROG3^+^ progenitors in vitro and in vivo

**Fig. 1:**
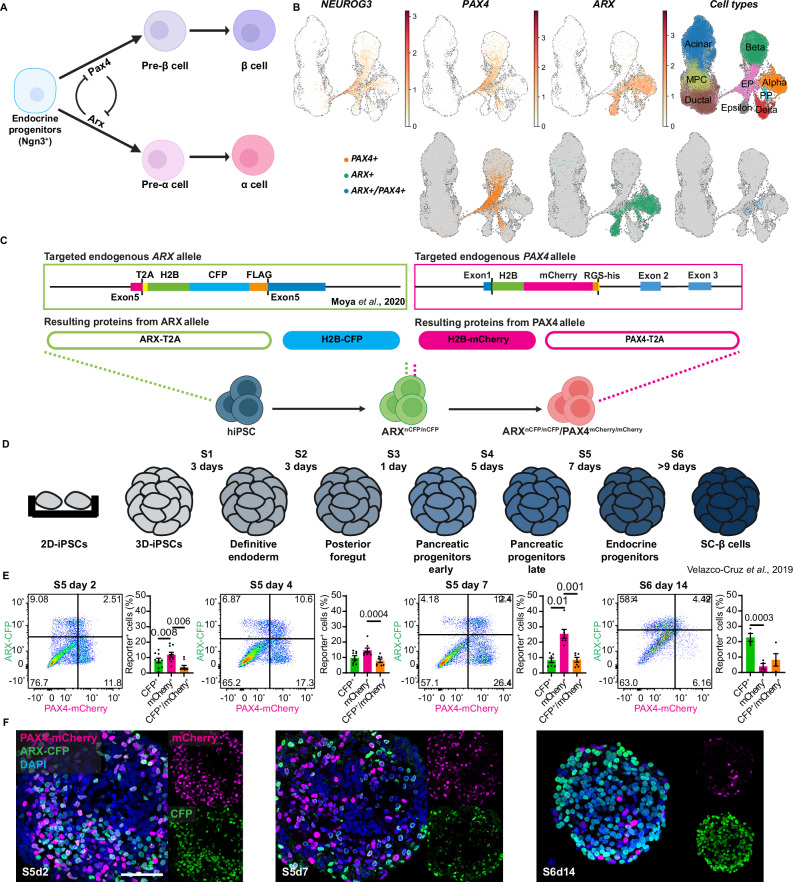
PAX4- and ARX-reporters have asynchronous expression patterns. **A** Postulated NGN3-PAX4-ARX signaling cascade involved in α and β cells differentiation in mouse. **B** Uniform Manifold Approximation and Projections (UMAPs) showing *NEUROG3, PAX4*, and *ARX* mRNA expression, as well as PAX4^+^, ARX^+^, and ARX^+^/PAX4^+^ cell populations within human pancreata ranging from 7- to 20-weeks post-conception. **C** Generation strategy of the *ARX*^*CFP/CFP*^*/PAX4*^*mCherry/mCherry*^ reporter hiPSC line via CRISPR-Cas9-based targeting starting from the *ARX*^*nCFP/nCFP*^ reporter cell line^41^. H2B-mCherry was inserted in exon 1 of the endogenous *PAX4* allele together with an RGS-histidine tag. Resulting proteins from both TFs alleles are shown. Two homozygous clones for both genetic alterations were generated, and one was used for all subsequent experiments. **D** Differentiation protocol as in Velazco-Cruz et al., 2019. **E** Representative flow cytometry plots and related quantifications of ARX-CPF and PAX4-mCherry reporter-positive cells during SC-β cell differentiation at the beginning (S5 day 2-S5d2), mid (S5d4), and end of EP stage (S5d7), and the SC-β cell stage (S6d14). *n* = 10, 8, 7, 4 distinct differentiation experiments, mean ± SE, repeated measures one-way ANOVA with Tukey multiple comparison test. **F** Representative maximum intensity projections of Z-stack confocal acquisitions of immunofluorescence (IF) staining for ARX-CFP and PAX4-mCherry in ARX/PAX4 reporter clusters at the indicated stages. Scale bars 50 µm.

PAX4 and ARX TFs govern pancreatic EP lineage fate decision toward α or β cells in the mouse^21^. PAX4 promotes β cell differentiation, while ARX promotes differentiation towards the α cell lineage^20,22,23^ (Fig. 1A). This model would require *PAX4* and *ARX* to be mostly co-expressed upon induction and segregation in early EPs to allow the two proteins to act on each other's *loci* and stochastically drive α or β cell fate commitment. To test if this minimal requirement is fulfilled during human pancreas development in vivo, we interrogated a recently published human atlas of foetal pancreas development^38,39^. We looked at the mRNA expression of *PAX4*, *ARX*, and *NEUROG3*, a marker of EPs and master regulator of endocrine induction, in single-cell RNA-sequencing (scRNA-Seq) datasets (Fig. 1B; Supplementary Fig. 1A). This analysis showed expression of *PAX4* in EPs, early β cells, and early δ cells, but also revealed a transient *PAX4* co-expression with *NEUROG3* in EPs and early β cells. On the other hand, *ARX* expression was restricted to a discrete subset of EPs, α and ε cells. In addition, very few late EP cells showed co-expression of *PAX4* and *ARX* in this dataset. Taken together, these observations suggest that in humans, the commonly accepted mode of action of PAX4 and ARX (Fig. 1A) is likely not accurate, as the two opposing TFs are not largely co-expressed in early *NEUROG3*-positive EPs.

Due to the several limitations concerning the study of human endocrinogenesis in vivo, we took advantage of established in vitro human β cell differentiation protocols to study α *versus* β cell fate decision and to analyse the role of the TFs PAX4 and ARX in this context. We first interrogated an already published scRNA-Seq dataset of human PSC lines undergoing β cell differentiation in vitro^40^. We looked at the expression of *NEUROG3, PAX4*, and *ARX* in stem cell-derived EPs (stage 5–S5). In line with our in vivo findings, *NEUROG3* and *PAX4* were largely co-expressed in early, mid, and late EPs, while *ARX* was expressed in α and δ cells expressing *Somatostatin* (*SST*) and *Haematopoietically Expressed Homeobox* (*HHEX*) (Supplementary Fig. 1B-C). To temporally resolve and mechanistically understand how, and to which extent, ARX and PAX4 control α and β cell fate decision at the single cell level, we generated a homozygous human iPSC line expressing the CFP reporter for ARX and the mCherry reporter for PAX4 (*ARX*^*nCFP/nCFP*^; *PAX4*^*mCherry/mCherry*^), hereafter called “ARX/PAX4 reporter”. Using CRISPR/Cas9 engineering and our previously published *ARX*^*nCFP/nCFP*^ reporter iPSC line^41^, we inserted the coding sequence of a nuclear-tagged (histone 2B-H2B) mCherry fluorescent protein in exon 1 of the endogenous *PAX4* allele (Fig. 1C). The strategies to target both *ARX* and *PAX4* involved an insertion of a T2A sequence between the TF and the respective fluorescent protein. Consequently, ARX and PAX4 proteins are not fused to the fluorescent reporters, but TFs and reporters are co-translated. Using this strategy and taking advantage of the long half-life of the fluorescent proteins integrated into histone 2B, we aimed to simultaneously look at the rate of transcription of both genes encoding the TFs, and lineage trace ARX^+^ α cells and PAX4^+^ β cells during endocrine induction and lineage segregation. The generated line did not carry any chromosomal aberration and passed all routine quality controls (Supplementary Fig. 1D–F). Importantly, the ARX/PAX4 reporter iPSC line is pluripotent and can differentiate into all three germ layers (Supplementary Fig. 1H, I).

To study in greater detail *PAX4* and *ARX* expression during β cell differentiation, we differentiated the ARX/PAX4 reporter iPSC line using an adapted version of a previously published β and islet cell differentiation protocol in 3D culture^17^ (Fig. 1D). We monitored the presence and reporter activity of mCherry and CFP as a proxy of *PAX4* and *ARX* expression, respectively, using live flow cytometry and immunofluorescence (IF) (Fig. 1E, F). Cell clusters at early PP stage (S3) showed less than 1% positive cells for both mCherry and CFP by live flow cytometry (Supplementary Fig. 2A). The percentage of fluorophore-positive cells started to increase in the transition between early and late PPs (day 2 of S4 – S4d2) (Supplementary Fig. 2A). While the relative number of cells positive for mCherry peaked at the EP stage (S5d7), the relative number of CFP-positive cells peaked at the SC-β cell stage (S6d14) (Fig. 1E, F; Supplementary Fig. 2C). For the CFP reporter, this major peak was preceded by another minor peak at S4d5, probably due to the specification of ARX^+^ ε progenitors, as suggested by a recent scRNA-Seq on embryonic human samples^42^. Since fluorescent reporters are transcribed and translated together with the TFs themselves (Supplementary Fig. 2B, C), the presence of the reporters is a proxy for TF activity. Therefore, these data suggest that *PAX4* and *ARX* expression patterns, and therefore their activity, are asynchronous. For comparison, we quantified mRNA and protein levels of TFs and their fluorophores in the ARX/PAX4 reporter line and compared them with the levels of the endogenous PAX4 and ARX proteins in wild-type clusters (XM001^43^) (Fig. 1F; Supplementary Fig. 2B–D). We analysed multiple time points during the differentiation protocol toward SC-β cells and found similar expression patterns. Due to the fusion of the fluorescent reporter to histone H2B, both nuclear localized mCherry and CFP seemed to be more stable and detectable for a longer timespan compared to the endogenous TFs at the protein level, making mCherry and CFP lineage tracers for both PAX4^+^ and ARX^+^ cell lineages in the ARX/PAX4 reporter cell line (Supplementary Fig. 2). Importantly, onset of *ARX* and *PAX4* expression and their protein synthesis are accurately reflected by our double reporter system which allows us to dynamically resolve temporal changes of the two lineage determining TFs, and consequently, their downstream GRNs. Together with the human foetal expression patterns, this data suggests a first wave of expression of *ARX* to allow the specification of the ε progenitors, followed by a sequential expression of *PAX4* and *ARX* during human α *versus* β cell specification and differentiation in which *PAX4* is transiently co-expressed with *NEUROG3* and transcriptionally active in early- and mid-EPs, while *ARX* is expressed and active in α cell progenitors at later stages of differentiation. If true, these data would support a different model for human EP differentiation towards the α or β cell fate than proposed in vivo in the mouse (Fig. 1A).

### Single-cell multiomic analysis suggests ARX repression of *PAX4* during in vitro human SC-β cell differentiation

**Fig. 2:**
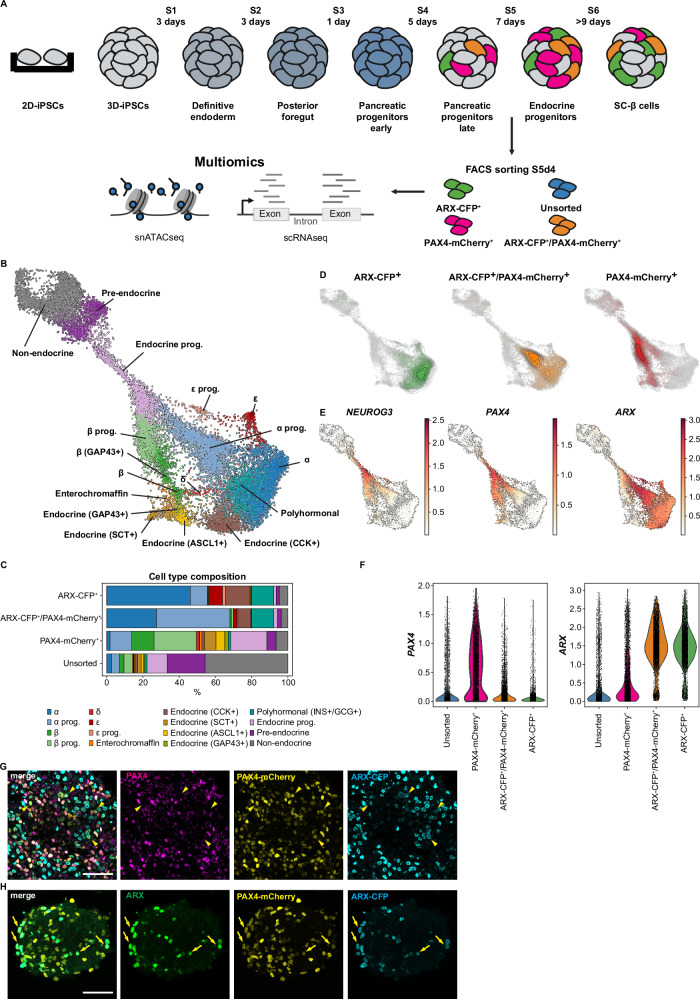
Single cells multiomics suggests ARX repression of PAX4 during in vitro SC-β cell differentiation. **A** Experimental strategy used for multiome analysis of ARX-CFP^+^, ARX-CFP^+^/PAX4-mCherry^+^ and PAX4-mCherry^+^ FACS-sorted fractions. Unsorted live cells were used as control. **B** Annotated multiomic UMAP showing the integration of all samples to identify cell clusters. **C** Relative frequencies of cell types in each dataset as per annotated UMAP in B. **D** Embedding densities of PAX4-mCherry^+^, ARX-CFP^+^/PAX4-mCherry^+^, and ARX-CFP^+^ samples. **E** UMAPs showing *NEUROG3* and *PAX4* expression starting from EPs, and *ARX* expression in a subset of EPs and α-cell progenitors. **F** Violin plots showing normalized *PAX4* and *ARX* expression in ARX-CFP^+^, ARX-CFP^+^/PAX4-mCherry^+^, PAX4-mCherry^+^ and unsorted fractions. Representative maximum intensity projections of Z-stack confocal acquisitions of IF staining for endogenous PAX4 (**G**) and ARX (**H**), together with PAX4-mCherry and ARX-CFP in ARX/PAX4 reporter clusters at mid S5. Yellow arrowheads in G show double-positive cells for PAX4-mCherry/ARX-CFP but negative for endogenous PAX4. Yellow arrows in H show double-positive cells for PAX4-mCherry/ARX-CFP that are instead positive for endogenous ARX. Scale bars 50 µm.

To test our hypothesis and characterize the regulatory program governing α *versus* β cell fate acquisition during differentiation of human SC-islets, we took advantage of the ARX/PAX4 reporter iPSC line. Due to high expression of the master regulator of endocrine differentiation *NEUROG3* at this stage, S5d4 clusters should be enriched in cells going through the transition from late PPs towards EPs (Fig. 1D, E), making this differentiation time point ideal to study the dichotomy between ARX- and PAX4-mediated α *versus* β lineage determination. We therefore used fluorescence activated cell sorting (FACS) to isolate PAX4-mCherry^+^, ARX-CFP^+^ and PAX4-mCherry^+^/ARX-CFP^+^ cells at S5d4 and performed single cell multiomic analysis of all three fractions together with an unenriched sample (Fig. 2A; Supplementary Fig. 3A). To simultaneously monitor gene expression and chromatin accessibility in single cells, we conducted paired single-nucleus RNA-seq and single-nucleus assay for transposase-accessible chromatin using sequencing (snATAC-seq) resulting in a total of 18’544 cells. We first performed cluster analysis of the two datasets separately, and then, to ensure more precise identification of cell types^44,45^, we integrated both datasets (Supplementary Fig. 3B). This identified sixteen distinct cell clusters (Fig. 2B). One cluster was comprised of non-endocrine cells and one of pre-endocrine cells. One EP population was annotated. Two α cell clusters were present: α progenitors and α cells. In addition, three β cell clusters were annotated: β progenitors, β cells expressing the neuronal marker *Growth Associated Protein 43* (*GAP43*), and β cells. Our dataset also contained ε progenitor and ε cell clusters, together with other endocrine cells characterized by the expression of other hormones/genes and polyhormonal cells (Fig. 2B; Supplementary Fig. 3C). We then looked at the relative frequency of cell types across our FACS-sorted fractions (Fig. 2C, D; Supplementary Fig. 3D). As expected, half of the control unsorted sample was comprised of non-endocrine cells, while the other half of EPs and endocrine cells. The PAX4-mCherry^+^ fraction contained mostly EPs, β cell progenitors, and β cells. The ARX-CFP^+^ fraction contained α cells, endocrine cells expressing the hormone *cholecystokinin* (*CCK*), polyhormonal cells, α cell progenitors, and ε cells, which were previously hardly detected in any other single cell datasets^18,40,44^. On the other hand, and similarly to the ARX-CFP^+^ sample, the fraction enriched for double positive cells for both ARX and PAX4 reporters (ARX-CFP^+^/PAX4-mCherry^+^) was constituted mainly by α cell progenitors, α cells, polyhormonal and *CCK*-expressing cells (Fig. 2C, D; Supplementary Fig. 3D). In agreement with our flow cytometry and IF analysis (Fig. 1E, F) and the publicly available scRNA-seq previously analysed (Supplementary Fig. 1B, C), *PAX4* expression mostly coincided with *NEUROG3* expression in EPs and subsets of α and β cell progenitors, while *ARX* expression was elevated in α cell progenitors, α cells, polyhormonal, *CCK*, and *GHRL* expressing cells (Fig. 2E; Supplementary Fig. 3E–H). Interestingly, *PAX4* expression was present in the PAX4-mCherry^+^ fraction but absent from the fraction enriched for ARX-CFP^+^/PAX4-mCherry^+^ cells (Fig. 2F). To further validate this evidence by IF, we stained clusters from the ARX/PAX4 line at mid S5 for both the endogenous TFs and their fluorescent reporters. We found that mCherry^+^/CFP^+^ cells showed no staining for endogenous PAX4 (Fig. 2G, yellow arrowheads). This data was further validated by the very low presence (on average ≤1% of the entire population) of ARX^+^/PAX4^+^ cells in wildtype clusters through the entire differentiation (Supplementary Fig. 2D). In conclusion, multiomic analysis showed that (1) simultaneous *ARX* and *PAX4* mRNAs and reporter activity reflects TF transcription and activity in our system, (2) *PAX4* expression is transient expressed in EPs, therefore mCherry is a transient *PAX4* reporter in emerging α cell progenitors, and (3) *PAX4* expression, and therefore *mCherry* activity, are maintained in β cell progenitors.

To validate our scRNA-Seq data, we performed bulk proteomic analysis of FACS-sorted fractions at S5d4 (Supplementary Fig. 4A). Gene ontology analysis on differentially abundant proteins between unsorted and enriched samples revealed commitment towards β cells of PAX4-mCherry^+^ fractions, and α cells of both the PAX4-mCherry^+^/ARX-CFP^+^ and ARX-CFP^+^ fractions (Supplementary Fig. 4). In agreement with EPs being postmitotic and less adhesive to the extracellular matrix (ECM)^46–51^, all three samples showed downregulation of proteins related to ECM organization and cell cycle (Supplementary Fig. 4). Also, the transcriptomic (Fig. 2C, D) and proteomic gene ontology data of the PAX4-mCherry^+^/ARX-CFP^+^ and ARX-CFP^+^ samples showed very similar profiles (Supplementary Fig. 4).

In accordance with our hypothesis, these data suggest a sequential expression of *PAX4* and *ARX* during human SC-α and β cell differentiation. In this scenario, the transient action of PAX4 would (directly or indirectly) induce *ARX* expression only in some EPs. Once translated, ARX would be responsible for the direct or indirect repression of *PAX4* mRNA expression in α cell progenitors, promoting α cell differentiation.

### ARX TF action determines α *versus* β cell fate decision

**Fig. 3:**
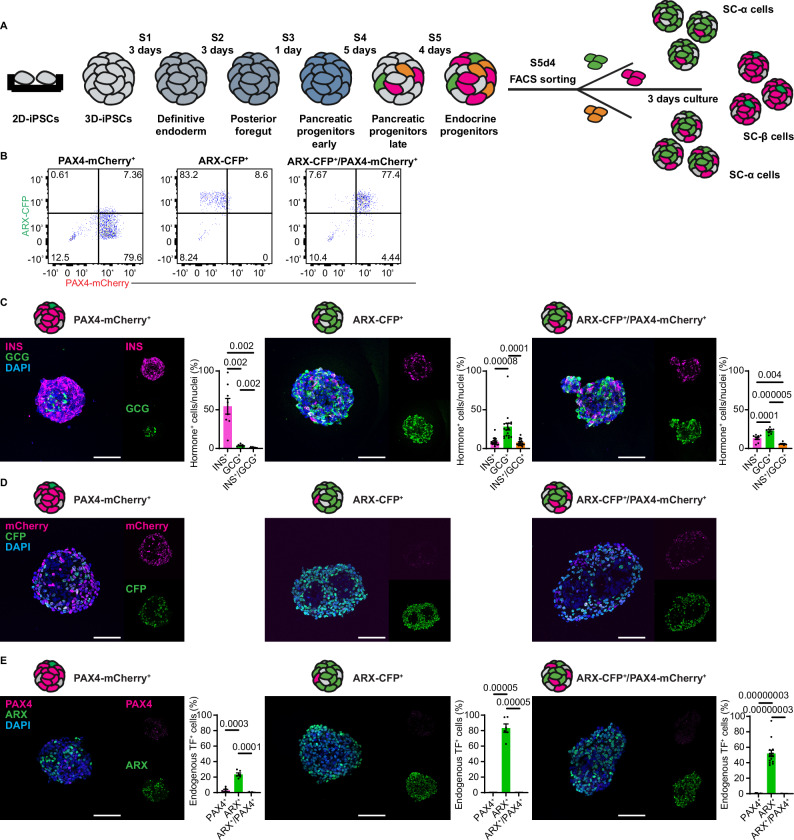
ARX expression coordinates α versus β cell fate decision. **A** Experimental strategy for potency assay of ARX-CFP^+^, ARX-CFP^+^/PAX4-mCherry^+^ and PAX4-mCherry^+^ FACS-sorted fractions. **B** Representative flow cytometry plots of fraction purity directly after sorting. **C**–**E** Representative maximum intensity projections of Z-stack confocal acquisitions of IF and related quantifications on FACS-sorted ARX-CFP^+^, ARX-CFP^+^/PAX4-mCherry^+^ and PAX4-mCherry^+^ fraction-derived clusters showing insulin and glucagon (**C**), ARX-CFP and PAX4-mCherry (**D**), and endogenous PAX4 and ARX (**E**) after 3 days in culture. *n* = 3 biological replicates from distinct differentiations; each dot represents a single technical replicate (cells cluster) from all 3 biological replicates. INS insulin, GCG glucagon. Data are presented as mean ± SE; repeated measures one-way ANOVA with Tukey multiple comparison test. Scale bars: 50 µm.

To functionally test this hypothesis, we performed in vitro potency assays. We sorted PAX4-mCherry^+^, ARX-CFP^+^, and PAX4-mCherry^+^/ARX-CFP^+^ in the middle of the EP stage (S5d4) and differentiated them toward early SC-islet state for 3 days (Fig. 3A, B). As expected, most cells within the PAX4-mCherry^+^-enriched cultures differentiated into insulin-producing SC-β cells (Fig. 3C). On the other hand, most cells within both ARX-CFP^+^- and PAX4-mCherry^+^/ARX-CFP^+^-enriched cultures differentiated into glucagon-producing SC-α cells (Fig. 3C). IF for PAX4 and ARX and their reporters revealed that, while single positive cells for ARX-CFP^+^ kept producing CFP and ARX during the entire culture, PAX4-mCherry^+^/ARX-CFP^+^ cells eventually decreased both PAX4 and mCherry production, producing mostly cells positive for CFP and ARX by the end of the culture (Fig. 3D, E). On the other hand, as expected by the expression of *PAX4* in both NEUROG3^+^ endocrine progenitor and β progenitors at S5d4, PAX4-mCherry^+^ clusters at S5d7 were composed mostly by mCherry^+^ cells, as well as few cells positive for CFP and ARX (Fig. 3D, E). Confirming both the transient expression of PAX4 during differentiation and the persistence of its mCherry reporter after *PAX4* transcription downregulation (Fig. 2), cells positive for endogenous PAX4 were mostly absent from all clusters at S5d7 (Fig. 3E), while mCherry^+^ cells persist in PAX4-mCherry^+^- and PAX4-mCherry^+^/ARX-CFP^+^-enriched cultures (Fig. 3D). Similar results were obtained with in vitro potency assays following sorting of PAX4-mCherry^+^, ARX-CFP^+^, and PAX4-mCherry^+^/ARX-CFP^+^ at the of the EP stage (S5d7) and differentiation toward early SC-β cells separately for 2 weeks (Supplementary Fig. 5).

Taking together, multiomics, proteomics, and potency assays suggest the lack of stochasticity in the expression of *PAX4 versus ARX* in double-positive cells, and highlight ARX expression and activity as a major determinant of α cell fate determination. Consequently, cells expressing both TFs in the mid/late EP stage of endocrinogenesis commit to the α cell lineage.

### ARX and PAX4 participate in a GRN amenable to manipulation

**Fig. 4:**
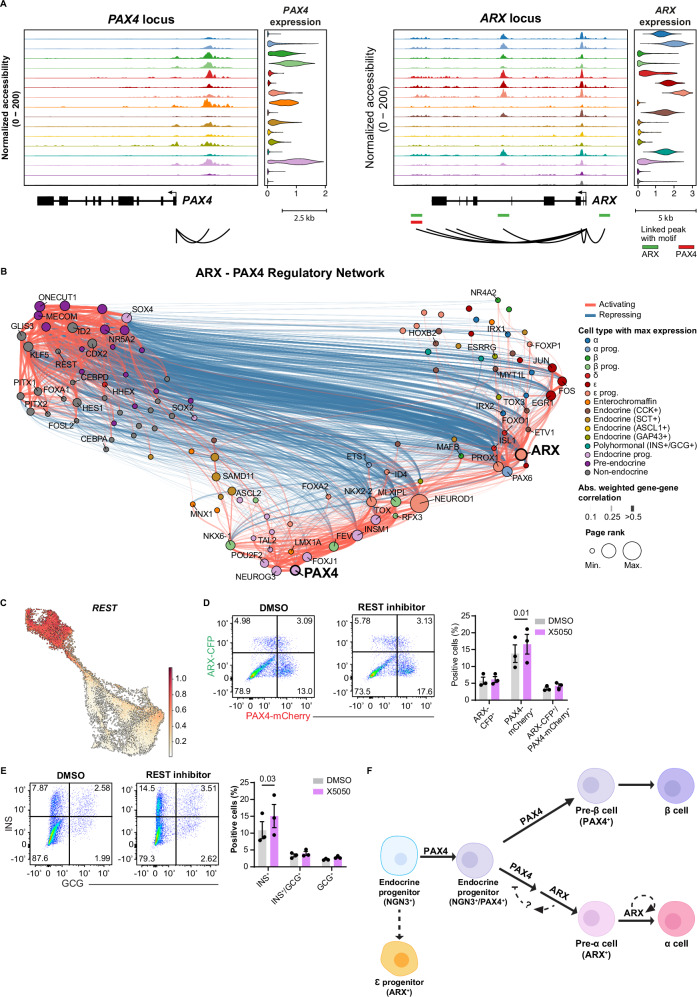
ARX and PAX4 participate in a GRN that can be manipulated. **A** ATAC sequencing peaks showing chromatin accessibility of the *PAX4* and *ARX* loci in each cell type. Arcs link significantly correlated peaks (i.e., putative CREs) to the TSS. Putative CREs with PAX4 or ARX motifs are shown separately. **B** Prediction of a GRN involving PAX4 and ARX using multiomic datasets from ARX-CFP^+^, ARX-CFP^+^/PAX4-mCherry^+^ and PAX4-mCherry^+^ FACS-sorted fractions. **C** UMAPs showing enriched *REST* expression in non-endocrine and pre-endocrine cells. Representative flow cytometry plot (left) and related quantification (right) of the percentage of ARX-CFP^+^, ARX-CFP^+^/PAX4-mCherry^+^ and PAX4-mCherry^+^ cells (**D**) or INS^+^, GCG^+^, and bihormonal cells (**E**) at S5d7 (EPs) after treatment with REST inhibitor (X5050) starting from S5d1. *n* = 3 biological replicates from distinct differentiations, mean ± SE, two-way ANOVA with Šídák multiple comparison test. **F** Working model of ARX and PAX4 mechanism of action during human in vitro cell fate decision per our data. Dashed lines represent regulations suggested by our data but that were not biochemically validated. Question marks represent unknown TFs mediating the indirect repression of *PAX4* transcription by ARX. INS insulin, GCG glucagon.

To study if and how ARX and PAX4 modulate each other’s molecular and cellular activity, we queried the single-nuclei ATAC-Seq modality for potential PAX4 and ARX binding sites within each other’s putative cis-regulatory elements (CRE). While we did not find any elements with ARX or PAX4 motifs in the direct vicinity of *PAX4*, we found motifs of both TFs in putative CREs in close proximity of the *ARX* gene (Fig. 4A, Supplementary Fig. 6). The positive correlation between *ARX* gene expression and the accessibility of the linked CREs with *ARX* motifs suggests an autoregulatory loop reinforcing *ARX* expression. On the other hand, we did not find a significant correlation between the expression of *PAX4* and the accessibility of the CRE with the PAX4 motif, indicating that PAX4 is not involved in the regulation of *ARX* directly. When we expanded our analysis to more distal putative CREs (up to 100 kb from the transcription starting site), we found an ARX motif in a peak region linked to *PAX4*, however, there was no correlation between the accessibility of the CRE and *ARX* expression (Supplementary Fig. 6C, D). We thus concluded that there are no direct regulatory interactions between PAX4 and ARX in the region we analysed, and that ARX might promote its own expression via a positive feedback loop (Fig. 4A). We then used Pando^52^ to infer a global GRN to predict PAX4 and ARX target genes during α and β cell differentiation (Fig. 4B). In line with our previous analysis, the GRN suggested that ARX and PAX4 do not directly interact and that several intermediate TFs are involved in *ARX* and *PAX4* transcriptional regulation.

Our GRN allows for identification of putative upstream regulators of *ARX* and *PAX4*. In particular, REST seems to repress endocrine genes that are also direct upstream regulators of *PAX4* (*FEV, INSM1, NKX2-2*), while promoting non-endocrine marker genes (*HES1, ONECUT1, SOX9*) in non-endocrine cells. This circuitry suggests REST as keeper of pancreatic identity and repressor of endocrine induction in PPs, as well as an indirect upstream regulator of *PAX4* expression. This correlation is in line with recent literature where REST inhibition in pancreatic organoids and Zebrafish embryos, using the small molecule X5050, increases endocrinogenesis^53,54^. To test the validity of our GRN and our ability to manipulate it, we inhibited REST in emerging ARX/PAX4 reporter SC-islets at S5. Treatment with X5050 resulted in small but significant increases of both PAX4-mCherry^+^ cells and insulin^+^ cells at S5d7, compared to cells treated with DMSO vehicle alone (Fig. 4D, E), highlighting a previously unrecognized indirect role of REST upstream of PAX4. Thus, our GRN analysis using the ARX/PAX4 double reporter line identifies druggable targets to manipulate and study human α *versus* β cell fate decision in vitro.

Taken together, these results led us to hypothesize a model of human α *versus* β cell fate allocation in which PAX4 and ARX do not stochastically cross-inhibit each other. Instead, we suggest that *PAX4* expression in early EPs temporally precedes *ARX* expression in a few late EPs and α progenitors. ARX in turn would directly reinforce its own expression, dominating differentiation toward the α cell lineage (Fig. 4F).

### Artemether has a profound effect on endocrine lineage induction and segregation

**Fig. 5:**
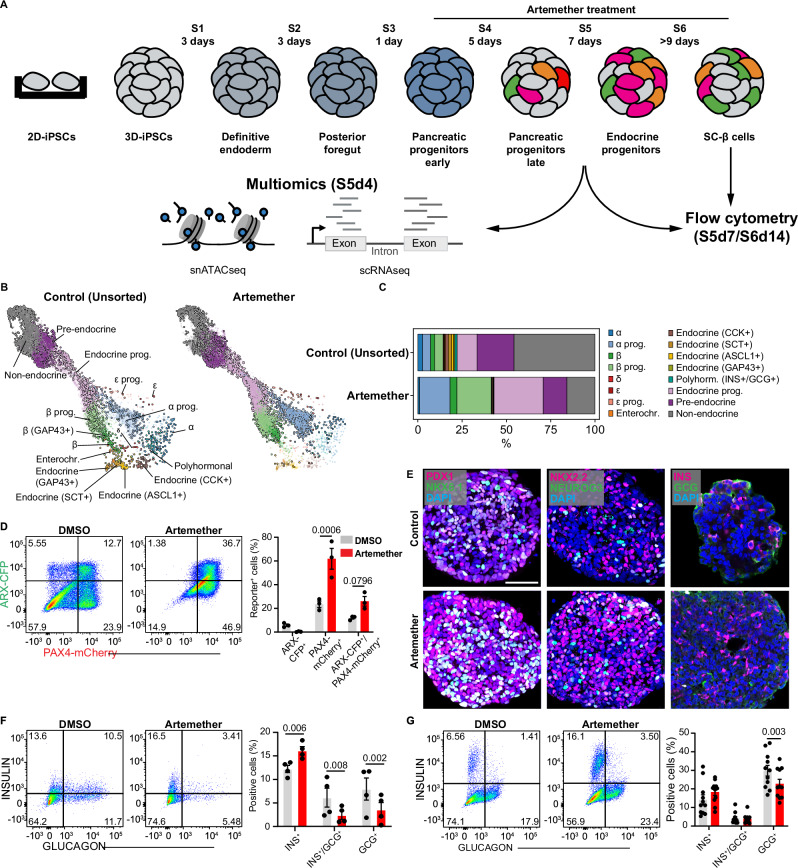
Artemether has a profound effect on endocrine lineage induction and segregation. **A** Experimental strategy for artemether treatment followed by multiomic analysis at S5d4 and flow cytometry analysis at S5d7. Multiome UMAPs of unsorted (4’086 cells) and artemether-treated samples (4’379 cells) (**B**) and relative cell type composition (**C**). **D** Representative flow cytometry plots and related quantification of the percentage of ARX-CFP^+^, ARX-CFP^+^/PAX4-mCherry^+^ and PAX4-mCherry^+^ cells at S5d7 after artemether treatment; *n* = 3. **E** Representative maximum intensity projections of Z-stack confocal acquisitions of IF staining showing EP markers (PDX1, NKX6.1, NKX2.2, and NEUROG3), insulin and glucagon hormones within artemether-treated and control clusters at S5d7. Representative flow cytometry plots and related quantification of hormone-positive cells at S5d7 (**F**) and S6d14 (**G**) after continuous treatment with artemether starting from S4; *n* = 4, 11. For **D**, **F**, **G**, data are presented as mean ± SE; two-ways ANOVA with Šídák multiple comparison test. INS insulin, GCG glucagon. Scale bars: 50 µm.

The generation of the double ARX/PAX4 reporter iPSC line opens avenues to study simultaneously lineage trajectories on temporal resolved single-cell level and the more general biological consequences of small-molecule treatments on α *versus* β cell fate decision during SC-islet differentiation. Here we tested the use of the FDA-approved antimalarial drug artemether, which has been reported to trigger α-to-β transdifferentiation in some but not all studies^32–35,37^. Therefore, we continuously treated ARX/PAX4 differentiating reporter cells starting from early PP until SC-β cell (S4 to end of S6) stage and performed multiomics analysis (scRNA-seq and snATAC-seq) on cells derived from unsorted clusters at S5d4 and live flow cytometry at S5d7 and S6d14 (Fig. 5A; Supplementary Fig 7B). Multiomic analysis at S5d4 showed increased endocrine, β, and α progenitor cell numbers at the expense of both non-endocrine and more differentiated cell types upon artemether treatment (Fig. 5B, C). Accordingly, differential gene expression analysis of each untreated cluster *versus* its artemether-treated counterpart revealed increased expression of EP markers (such as *NEUROG3* and *Insulinoma-associated 1* - *INSM1*) upon treatment, while hormone expression levels were reduced (Supplementary Fig. 7C). Together, these data suggest a remarkable increase in endocrine induction and slower differentiation of artemether-treated cells. Confirming this hypothesis, flow cytometry analysis three days later (S5d7) revealed a significantly increased percentage of PAX4-mCherry^+^ cells upon artemether treatment, suggesting an enrichment for both endocrine and β cell progenitors (Fig. 5D). We confirmed this hypothesis with flow cytometry and IF analysis, which also revealed increased number of cells positive for EP markers (NEUROG3, NKX2.2, NKX6.1) and insulin, with a reduction in the percentage of glucagon-positive cells and bihormonal cells (insulin^+^/glucagon^+^) (Fig. 5E, F). Continuous treatment with artemether until the end of S5 (S5d7) or the end of the culture at S6d14 resulted in a strong reduction of glucagon-positive cells and a trend towards increased number of insulin-positive cells, as shown by flow cytometry (Fig. 5G; Supplementary Fig 8A–C), suggesting that EPs enriched at both S5d4 and S5d7 during artemether treatment would preferentially differentiate towards the β cell lineage. This increase in the number of insulin-positive cells did not result in an improved glucose-stimulated insulin secretion (GSIS) of artemether-treated clusters (Supplementary Fig. 8D, E). Taking together, these data suggest that artemether treatment has an impact on α *versus* β cell fate decision without affecting GSIS.

Due to the current controversy about artemether's potential to induce α-to-β cell transdifferentiation^32,35,36^, we tested the ability of artemether to induce transdifferentiation of any ARX-CFP^+^ cells (fated to become α cells) into β cells in our in vitro culture. We sorted ARX-CFP^+^ together with PAX4-mCherry^+^/ARX-CFP^+^ cells at S5d7 and then cultured these cells for 2 weeks in S6 media with artemether or DMSO (Supplementary Fig. 8F). Quantification of IF on S6d14 clusters revealed increased numbers of insulin-positive cells without a modulation of either bihormonal cells (INS^+^/GCG^+^) or glucagon-positive cell numbers upon artemether treatment compared to DMSO-treated controls (Supplementary Fig. 8G). Due to the unchanged number of both bihormonal and glucagon^+^ cells, this result rules out involvement of α-to-β transdifferentiation upon artemether treatment in vitro following direct β cell differentiation of human iPSCs. We attribute the small increase in insulin-positive cell numbers to the action of artemether on the residual EPs present in the culture, due to the asynchronous nature of in vitro differentiation together with the not 100% purity of our sorting condition.

### Artemether induces cell proliferation while protecting against cell death

**Fig. 6:**
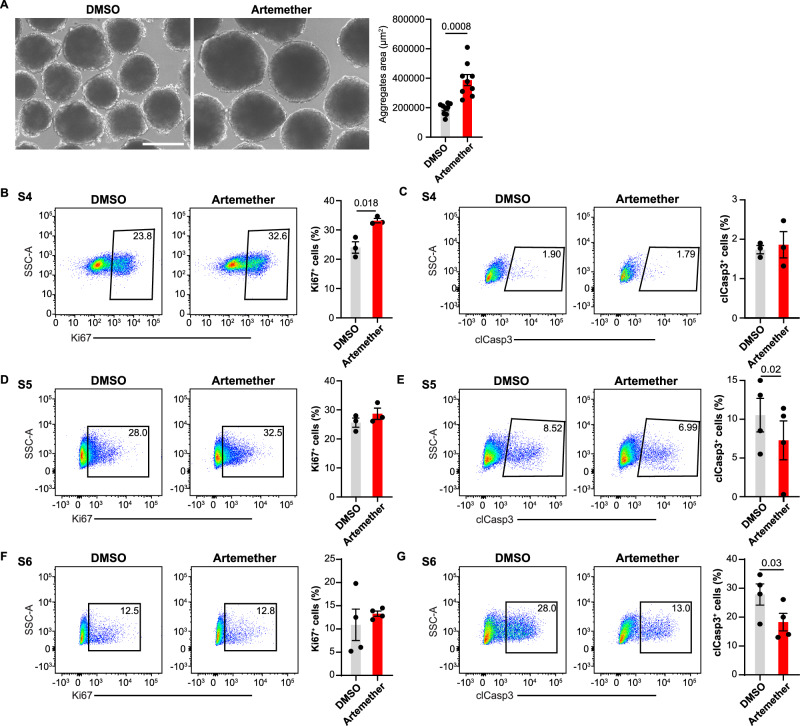
Artemether induces cell proliferation while protecting against cell death. **A** Representative bright-field pictures and related area quantification of clusters treated with artemether or DMSO at S5d7. Note the size increase and the increased sphericity of the artemether-treated clusters; *n* = 9. Representative flow cytometry plots and related quantifications of the percentage of Ki67-positive cells or clCasp3-positive cells at S4d5 (**B**, **C**), S5d7 (**D**, **E**), and S6d14 (**F**, **G**) after DMSO or artemether treatment; *n* = 3, 3, 3, 4, 4, 4. For **A**–**G**, data are presented as mean ± SE; paired Student’s *t*-test, two-tailed. Scale bar: 300 µm.

In addition to the altered composition of SC-islets, artemether-treated clusters were bigger than DMSO-treated controls (Fig. 6A). To understand what drives this phenotype, we quantified cell proliferation and death using flow cytometry. The percentage of proliferating cells (Ki67^+^) was increased specifically at S4d5 (Fig. 6B, D, F), whereas the percentage of apoptotic cells (cleaved Caspase3 - clCasp3^+^) was decreased at the EP (S5d7) and SC-β cell stage (S6d14) in artemether-treated samples compared to DMSO-treated (Fig. 6C, E, G).

Since artemether was reported to act through the γ-aminobutyric acid (GABA) signalling to modulate α cell identity^32^, we tested if manipulation of GABA signalling would phenocopy our treatment with artemether during SC-islets differentiation. Treatment of the ARX/PAX4 reporter line with GABA per se at S4 and S5 did not affect the number of ARX^+^ and PAX4^+^ cells at S5d7 (Supplementary Fig. 9A, B). Interestingly, both treatments with Picrotoxin and Baclofen (a GABA_A_ antagonist and a GABA_B_ agonist, respectively) resulted in an increased number of ARX^+^/PAX4^+^ and ARX^+^ cells (Supplementary Fig. 9C). Since treatments with both a GABA antagonist and an agonist showed the opposite effect compared to treatment with artemether, we excluded GABA signalling as a potential mechanism of action of artemether for induction of endocrine progenitors in vitro.

Together, we showed how artemether treatment could improve SC-β cell differentiation protocols: it promotes PP expansion, increases EP induction and positively affects SC-islets cellular composition, independently of GABA signalling.

## Discussion

The role of ARX and PAX4 during human pancreas endocrinogenesis has not been fully understood; their upstream and downstream regulation has not been characterized, and the dichotomy between the two TFs has never been fully demonstrated. Here, we generated a human iPSC double reporter line allowing to test the dogma by which the activity of the two TFs ARX and PAX4 in EPs would stochastically drive α or β cell fate decision, respectively. Following multiomics analysis, we generated a GRN centred around ARX and PAX4 and formulated a revised model of human α *versus* β cell fate decision. We showcased the ARX/PAX4 reporter line both as a tool to dissect genetic regulation underlying human endocrinogenesis, and as a useful tool for drug discovery, uncovering its potential to improve SC-islet differentiation protocols. We showed artemether-driven EP induction and decreased α cell differentiation, together with PP expansion, suggesting that artemether has the potential to improve the yield of PPs and EPs during direct SC-β cell differentiation. Additional experiments would be needed to find the best duration and concentration of artemether treatment. On the other hand, the effect of artemether on *ARX* and *PAX4* transcription seems negligible in our system.

Our study showed that *ARX* and *PAX4* temporal and spatial expression patterns do not largely coincide during human endocrinogenesis. We proved, both in vitro and in vivo, that at the time of α *versus* β cell fate commitment, *PAX4* is transiently expressed in EPs shortly after *NEUROG3*, while *ARX* is mostly expressed in cells already committed to the α cell lineage. This data is in line with recent human first trimester foetal single cell multiomics data, highlighting conserved and divergent transcriptional regulations during human and murine endocrinogenesis^42^. Consequently, our work suggests *PAX4* as a transient EP marker, downstream of *NEUROG3*. Our model of human endocrinogenesis does not fully resemble the one from Ma and colleagues^42^, as our work did not completely characterize the *ARX*-positive ε cell differentiation branch. In line with the presence of this early branch establishing ε cell commitment before α and β cell fate decision, we detected ARX-CFP^+^ and ARX^+^ cells at S4 during differentiation of both the ARX/PAX4 reporter line and its parental iPSC line (Supplementary Fig. 2A, D). In addition, samples embedded within our multiomics analysis showed contribution to the *ε* progenitors and *ε* cell clusters only from the ARX-CFP^+^ sample but not from the ARX-CFP^+^/PAX4-mCherry^+^ (Fig. 2D). Our GRN found a feedforward loop between *NEUROG3* and *PAX4*, showing how both are each other’s targets. In addition, while PAX4 has been classically described as a repressor of hormone transcription^55–57^, it acts mainly as an activator at the EP stage in our system and GRN. On the other hand, ARX exhibits mainly a transcriptional repressor activity. To definitively test the model, Chromatin ImmunoPrecipitation (ChIP) assays would be the gold standard. Unfortunately, due to a lack of antibodies amenable to ARX and PAX4 pulldown, the necessary ChIP-Seq experiments are not possible in the ARX/PAX4 iPSC reporter cell line. Ideally, this technical roadblock could be circumvented by combining lineage tracing, cell sorting, and single-cell multiomic assays at multiple time points during in vitro differentiation. However, CHIP-qPCR performed on human small intestine organoids overexpressing FLAG-tagged ARX and/or FLAG-tagged PAX4 showed no direct interaction between ARX and PAX4 in enteroendocrine cells^58^, a closely related cell type to pancreatic endocrine cells. Moreover, CUT and RUN assays on the stable β cell line EndoC-βH1 transduced with FLAG-tagged PAX4 showed no binding of PAX4 to the *ARX locus*^59^. Within the same locus, the authors also showed no overlap between differentially expressed genes in PAX4 KO stem cell-derived EP and PAX4 accessible peaks identified in EndoC-βH1 cells overexpressing *PAX4*.

Previous studies reported KOs of *PAX4* and *ARX* in human stem cells. In line with ARX orchestrating α cell fate decision in humans, islets from patients with *ARX* mutations lack α and PP cells, a phenotype that human ESC KO for *ARX* do not resemble completely^60,61^. We observed some *Pancreatic Polypeptide Y (PPY)* expression within the GCG^+^ cells; however, these cells did not form a separate cluster. This is likely due to the optimization towards β cell differentiation of the protocol used. In line with our GRN showing a direct orchestration of β cell fate acquisition by PAX4, *PAX4* KO human hiPSC resulted in decreased β cell maturation and secretion together with upregulation of α cell-related genes^62^. Additionally, bulk RNA sequencing of *PAX4* KO human hiPSC showed an upregulation of *ARX* expression^59^, which is not in contrast with our model in which *PAX4* expression temporally precedes *ARX* expression but does not orchestrate it. In particular, in contrast to our use of single-cell multiomics, previous studies analysed the effect of *PAX4* modulations using bulk-based technologies^59,62,63^, precluding the discrimination between differences in gene expression due to transcriptional change at the single-cell level and changes due to variation in cell composition of different samples. This would explain why bulk analysis of *Pax4* KO mice, as well as *PAX4* KO and loss-of-function human cell lines, has increased α cell/bihormonal cell numbers and/or *ARX* expression. Methodology and cell source differences could therefore explain apparent discrepancies between our model and previous studies.

We showed how useful our ARX/PAX4 double reporter line could be as a drug screening tool. In combination with our GRN, it allows identification and testing of druggable hubs for the optimization of differentiation protocols, with the goal of generating SC-islets with proportions of α and β cells similar to human islets. It can also be used to simultaneously test the mechanisms of action and impact on SC-islet differentiation of putative α-to-β transdifferentiation inducers in a relevant human model system. Our GRN identified both uncharacterized and established regulators of endocrine induction and differentiation. As upstream regulators of *PAX4* we found multiple genes involved in pancreas development, such as Regulatory Factor X3 (RFX3)^64^, FEV Transcription Factor, ETS Family Member (FEV)^65,66^, INSM1^67–69^, Forkhead Box A2 (FOXA2)^70–72^, and SRY-Box Transcription Factor 4 (SOX4)^73–75^, as well as genes that have not yet been related to pancreas development, such as TAL BHLH Transcription Factor 2 (TAL2), Inhibitor of DNA Binding 4 (ID4), and Forkhead Box J1 (FOXJ1). Upstream of *ARX*, we found pancreas development-related genes such as Early Growth Response 1 (EGR1)^76^, FOXA2, IRX2^77^, ISL1^78^ and One Cut Homeobox 1 (ONECUT1)^79,80^, together with genes that have not yet been linked to pancreas development, such as MDS1 And EVI1 Complex Locus (MECOM), KLF5, and Paired Like Homeodomain 1 (PITX1).

Our GRN identified REST as an upstream repressor of the endocrine induction machinery (*FEV, INSM1, NKX2-2*) and a keeper of PP identity (*ONECUT1, SOX9, HES1*) in non-endocrine and pre-endocrine cells. The results of REST inhibition in our system further strengthen this hypothesis and are in line with the previous literature, where REST inactivation in pancreatic organoids induced *PDX1*, *NEUROG3* and *INS* transcription; treatment of Zebrafish embryos with X5050 showed enlarged secondary islets, and *Rest* KO mice have increased β cell mass^54^. This data highlights a previously unrecognized role of REST in preserving pancreatic cell identity while suppressing endocrine induction, and thus preventing endocrine cell fate acquisition in PP during human in vitro differentiation toward β cells.

On the other hand, the consequences of treatment with artemisinin and its derivatives on pancreatic cell fate decision and regeneration have been controversial. Artemether was reported to modulate endocrine cell fate commitment and promote β cell regeneration in immortalized α cell lines, murine and human primary islets, *Danio Rerio*, and mice^32–34^, with some studies identifying GABA signalling as an underlying mechanism of action^32,81,82^. Other studies reported instead dedifferentiation of murine and human primary islets^35^ or no changes upon treatment in vivo in mice^36,37^. While none of these studies looked at artemether potential during human endocrinogenesis, in this context our data provide strong evidence that artemether induces endocrine induction due to upregulation of *NEUROG3* and *INSM1* expression, and favours β cells at the expense of α cell differentiation in vitro, independently of GABA signalling. In addition, we showed that in our settings artemether does not induce α-to-β transdifferentiation.

Finally, our study provides detailed insights into the regulation of human α *versus* β cell fate decision both in vitro and in vivo that could pave the way to the establishment of better protocols for the differentiation of SC-islets for cell replacement therapy, as well as human-relevant drug testing tools for regenerative therapy purposes.

## Methods

### Ethics

The Ethics Committee of the Technical University of Munich positively voted on non-commercial research on human iPS cells (219/20 S). The donor of the original material expressed his/her informed consent to the generation of iPSCs from fibroblasts and further research activities. The material was donated under an epidemiological study at the University Hospital Tübingen.

### Generation of hiPSC line

HMGUi001-A-46 (a.k.a. ARX^nCFP/nCFP^; PAX4^mCherry/mCherry^) transgenic double-reporter hiPSC line was generated by insertion of mCherry in the PAX4 allele of HMGUi001-A-4 hiPSC line^41^. First, PAX4-single-guide RNA was cloned into the pu6-(BbsI) sgRNA-CAG-Cas9-Venus-bpA plasmid (Addgene plasmid #86986) via Gibson assembly, which introduced a double-strand break at the N-terminus of Exon 1, specifically after 10 bp from the start codon. The double-strand break was repaired by a targeting vector of the Histone 2B-mCherry-RGSHis-T2A construct flanked by a 703 bp 5’ homology arm (5’HA) and a 1008 bp 3’ homology arm (3’HA). Both homology arms were amplified from genomic DNA of HMGUi001-A hiPSCs (a.k.a. XM001) by PCR. hiPSC transfection was performed according to previous studies^83^. Briefly, HMGUi001-A-4 hiPSCs were seeded at a density of 0.4 × 10^6^ cells/well of a 6-well tissue culture plate with StemMACS™ iPS-Brew XF medium (Miltenyi Biotec cat#130-104-368) and 10 µM Y-27632 (SantaCruz cat# sc-281642A). The next day, the medium was replaced without Y-27632 for at least 4 hours before proceeding with the transfection. The targeting construct, H2B-mCherry-RGSHis-2A, and targeting plasmid, pu6-(Bbsl)PAX4-sgRNA-CAG-Cas9-Venus-bpA, were delivered via Lipofectamine™ Stem Transfection Reagent (Fisher Scientific, Cat# STEM00003). 48 hours after transfection, cells were treated with EDTA for 5 min and then harvested with StemMACS™ iPS-Brew XF supplemented with 10 µM Y-27632. The cell suspension was collected and centrifuged at 200 *g* for 4 min, and then the pellet was resuspended and filtered through low-attachment polypropylene flow cytometry tubes. Transfection efficiencies were assessed by recording the Venus-positive cells via flow cytometry. 3000-4000 Venus-positive cells were sorted and seeded onto pre-filled Geltrex (Thermo Fisher Scientific, Gibco cat#A1413302)-coated 10 cm tissue culture plate (Thermo Fisher Scientific, cat#150350). Sequences of the sgRNA and primers used for cloning and PCR can be found in Supplementary table 3-5.

### hiPSC culture

HMGUi001-A-46 hiPSCs were cultured in adhesion on tissue culture plates coated with 1:100 Geltrex in StemMACS™ iPSBrew XF culture medium at a density of 1 × 10^5^ cells/cm^2^ under standard culture conditions (37 °C, 5% CO2, and 95% humidity). The medium was replaced daily, and cells were passaged every 3-4 days once 70% confluency was reached. Cultures were rinsed with Dulbecco phosphate-buffered saline (DPBS, no Calcium, no Magnesium, Thermo Fisher Scientific, Gibco cat#14190094) and incubated with Accutase (Merck, Sigma-Aldrich cat# A6964-100ml) for 4-5 min at 37 °C. Dispersed hiPSCs were spun at 200 g for 4 min at room temperature. hiPSCs were resuspended in StemMACS™ iPS-Brew XF medium with 10 µM Y-27632 and seeded on pre-filled Geltrex-coated dishes. After 24 hours, the medium was replaced without Y-27632. For suspension culture HMGUi001-A-46 hiPSCs were dispersed using Accutase (Merck, Sigma-Aldrich cat# A6964-100ml) and counted using counting chambers (Hirschmann EM techcolor cat#8100204) 1 × 10^6^ cells/ml were seeded into prefilled 30 ml spinner flask with StemMACS™ iPS-Brew XF medium with 10 µM Y-27632 and placed onto magnetic stirrer platform (ABLE Biott cat# ABBWBP03N0S-6) at 60 rpm. Clusters of approximately 100 µm in diameter formed within 48 hours. The clusters were split every 3 days once a diameter of 200–300 um was reached. They were collected and rinsed with DPBS and incubated with Accutase for 7 min at 37 °C. The clusters were gently dispersed by pipetting and filtered using 100 µm cell strainer (Corning, cat# 431752). 1 × 10^6^ cells/ml cells were transferred into prefilled 30 ml spinner flask with StemMACS™ iPS-Brew XF medium with 10 µM Y-27632. The medium was replaced without Y-27632 within 48 h. HMGUi001-A-46 line was differentiated towards SC-β cells following the protocol outlined by Velazco-Cruz and colleagues^17^ after 2–3 passages in suspension culture. hiPSCs were seeded at a density of 1 × 10^6^ cells/ml in a 30 ml spinner flask for differentiation. Subsequently, differentiating clusters were transferred to 6-well Ultra-Low binding plates (Corning, Falcon cat#1015443) at a density of 5×10^6^ cells/well at the beginning of S4 or S5 for further treatments.

### Live flow cytometry and sorting

For live flow cytometry, 30 differentiating clusters were incubated with Accutase for 5 min in a water bath and dispersed to single cells. mCherry and CFP reporters were analyzed through PE-Texas Red and CFP channels, respectively, at S3-S6 using a FACS Aria III (Becton Dickinson) operated with BD FACSDiva software. A total of 30,000 events were recorded. FlowJo software (Becton Dickinson, version 10.7.1) was used for downstream analysis. For the sorting, clusters at S5 days 4 and 7 were dispersed into single cells and enriched based on CFP and mCherry fluorescence. The sorting parameters were established in FACS DIVA software. The clusters were dispersed using Accutase, then 10 × 10^6^ cells were resuspended in 2 ml of StemMACS™ iPS-Brew XF supplemented with 10 µM Y-27632 and transferred into a polystyrene round-bottom FACS tube (Corning, Falcon cat#3522235). PAX4-mCherry^+^, ARX-CFP^+^/PAX4-mCherry^+^, and ARX-CFP^+^ cells were then sorted into a polypropylene round-bottom FACS tube (Corning, Falcon cat#352063). Sorting accuracy was verified by re-analyzing 100 µl of each sorted population.

### Flow cytometry staining

Clusters were dispersed into single cells at indicated differentiation stages. Cells were blocked and permeabilized using Donkey Block (DB - PBS 1x, 10% FBS, heat inactivated, 0.1% Tween-20, 0.1% BSA, 3% Donkey serum) buffer containing 0.2% Triton X-100 (Merck, Sigma-Aldrich cat# T8787-250 ML) for one hour at room temperature. The cells were then incubated with primary antibodies diluted in DB + 0.2% Triton X-100 overnight at 4 °C. The next day, the cells were spun down, washed twice with DPBS, and incubated with secondary antibodies for two hours at room temperature. After staining, the cells were washed three times with DPBS and processed through flow cytometry. FACS Diva Software was used to plot the data, matching the specific fluorescence channels of the antibodies used. Controls included stained HMGUi001-A-46 hiPSCs and samples stained only with secondary antibodies. All experiments were analyzed using FlowJo software (Becton Dickinson, version 10.7.1). Lists of the used primary and secondary antibodies can be found in Supplementary Tables 1, 2.

### Clusters sectioning and staining

50 clusters were collected, washed with DPBS, and fixed with ice-cold 4% Paraformaldehyde (PFA; Boster cat # BSBTAR1068) for 20 min at room temperature. They were then immersed in a 10% (w/vol) D-(+)-Sucrose (ITW Reagents, PanReac AppliChem cat#57-50-1) solution for 2 hours at room temperature, followed by a 30% (w/vol) sucrose solution and incubated overnight in a 1:1 mixture of 30% sucrose and Tissue Freezing Medium (Leica, (14)020108926) in 4 °C. The next day, the clusters were embedded in cryomolds (Tissue-Tek cat#4566) filled with Tissue Freezing Medium, frozen on dry ice for 1 hour, and then transferred to −80 °C. The clusters were sectioned at 15 μm thickness using a Cryostat (Leica, CM1860). For staining, the sections were rehydrated in DPBS for 30 min and permeabilized in DPBS with 0.2% Triton X-100 and 1 M Glycin (Merck, Sigma-Aldrich cat#G8898-1KG). The slides were blocked with DB for 2 hours at room temperature and then incubated overnight at 4 °C with primary antibodies diluted in DB. The next day, the slides were washed in DPBS, incubated with secondary antibodies diluted in DB for 2 hours at room temperature, and stained using DAPI (2 µg/mL) for 20 min at room temperature. Finally, slides were mounted using Elvanol (25% Glycerol, 10% Mowiol, 100 mM Tris pH 8.0, 2% DABCO in ddH_2_O) and left to dry overnight at room temperature. Imaging was performed on a Zeiss LSM 880 at 63x magnification and 1x zoom, followed by image processing and analysis using Fiji. Zen Blue software or Fiji were used to generate maximum projections of Z-stack acquisitions.

### hiPSCs pluripotency analysis

Adherent HMGUi001-A-46 hiPSCs were dissociated using Accutase for 5 min at 37 °C, spun down at 200 *g* for 4 min, and resuspended in StemMACS™ iPS-Brew XF medium with 10 µM Y-27632. 1 × 10^6^ cells were stained with SSEA-4-488-FITC and TRA-1-60-PE FACS antibodies. The control samples were stained with isotype control (REA-Control)-FACS antibodies (Miltenyi Biotec cat# 130-104-610, 130-107-146) according to the manufacturer’s instructions (Miltenlyi Biotec). The samples were analyzed using FACS Aria III; 30000 events per sample were recorded.

### Three germ layer differentiation

HMGUi001-A-46 hiPSCs were differentiated towards endoderm, ectoderm, and mesoderm using the StemMACS™ Trilineage Differentiation Kit (Miltenyi Biotec, Cat# 130-115-660) according to the manufacturer’s instructions. Lists of the kits and materials used can be found in Supplementary table 6.

### Short tandem repeat polymerase chain reaction analysis

STR analysis was performed by the Genomic Core Facility, Helmholtz Zentrum München. Briefly, STR analysis was performed with the AmpFLSTR Identifiler PCR Amplification Kit (Thermo Fisher Scientific, Darmstadt, Germany) using 10 ng of genomic DNA according to the manufacturer’s recommendation. This kit amplifies 15 STR markers and amelogenin in one multiplex assay. Amplification products were mixed with GeneScan 500 LIZ size standard (Thermo Fisher Scientific, Darmstadt, Germany) and separated by size using capillary electrophoresis on a SeqStudio Genetic Analyzer (Thermo Fisher Scientific, Darmstadt, Germany). Data analysis was performed with GeneMapper 5.0 software (Thermo Fisher Scientific, Darmstadt, Germany).

### Karyotyping

Karyotyping was performed by the Institute of Human Genetics, Technische Universität München. Briefly, 10–30% confluent iPSCs were treated with colcemid to inhibit cell division, trypsinised, hypotonised, and fixed. Finally, the cell suspension was applied to several slides, dried overnight, and stained with Giemsa stain. At least 20 metaphases were microscopically acquired and analyzed using the Applied Spectral Imaging software.

### Static glucose-stimulated insulin secretion (GSIS) assay

Static GSIS assay was performed to assess the functionality of the differentiated aggregates at S6d14. 15–20 aggregates were starved in 200 μl KRBH buffer supplemented with 2.8 mM glucose for 1 hour under standard culture conditions. As an adaptation period after starvation, aggregates were incubated for 30 min at low glucose (2.8 mM glucose in KRBH buffer) and then for 30 min at high glucose (20 mM glucose in KRBH buffer). Aggregates were then incubated twice for 1 hour each at low and high glucose, with a washing step in KRBH buffer in between. Finally, aggregates were treated with 25 mM KCl for 30 min. All supernatants were collected for insulin quantification using ELISA. After GSIS, the aggregates were lysed overnight for DNA extraction. The secreted insulin was measured and then normalized to DNA content and the number of insulin-positive cells.

### RNA isolation, cDNA preparation and qPCR analysis

Total RNA for bulk analysis was extracted using the miRNeasy mini kit (Qiagen). Extracted RNA was reverse transcribed utilizing the SuperScript Vilo cDNA and cDNA synthesis kit (Life Technologies-Thermofisher Scientific). qPCR was performed using predesigned TaqMan probes (Life Technologies, Supplementary table 7) and 15 ng of cDNA per reaction. qPCR was performed using Viia7 (Thermo Fisher Scientific). Gene expression levels were determined by the 2^−ΔΔCT^ method following normalization to the reference gene (*GAPDH*).

### Western blotting

For western blot, aggregates were homogenized in RIPA buffer containing protease and phosphatase inhibitors. Cell lysates were then resolved by SDS-PAGE, transferred to PVDF membranes (BioRad), and incubated with blocking solution for 30 min at RT while rotating. Membranes were incubated with primary antibodies (Supplementary Table 1) overnight at 4 ᵒC. Samples were incubated with HRP-conjugated secondary antibodies (Supplementary Table 2) for 1–2 hours at RT while rotating. Protein bands were resolved using chemiluminescence reagent (Bio-Rad, Cat #1705061) reacting with HRP-conjugated antibodies.

### Single cell suspension preparation

The clusters were dissociated at S5 day 4 into single cells. 1 × 10^5^ PAX4-mCherry^+^, ARX-CFP^+^/PAX4-mCherry^+^, and ARX-CFP^+^ cells were sorted as described in section 3. Additionally, 1 × 10^5^ unsorted live cells were enriched using the DAPI channel, serving as control for multiomics.

### Single-cell multiome (paired snRNA/snATAC)

For nuclei isolation and library construction, a low-input nuclei isolation protocol adapted from 10X Genomics (CG000365) was performed. In brief, sorted cells were washed once with 1 mL PBS + 1% BSA, counted, centrifuged, and the supernatant was aspirated. Subsequently, the washed cell pellet was resuspended in chilled lysis buffer with 0.5x detergent concentration (10 mM Tris-HCl (pH 7.4), 10 mM NaCl, 3 mM MgCl₂, 0.05% Tween-20, 0.05% Nonidet P40 Substitute, 0.005% Digitonin, 1% BSA, 1 mM DTT, and 1 U/µl RNase Inhibitor; 50 μl/sample) and placed on ice for 5 min. Then, wash buffer (500 μL per sample) was added, and nuclei were centrifuged. To gradually change from wash to diluted nuclei buffer, cells were washed once in a 1:1 mixture of wash buffer and diluted nuclei buffer and subsequently once with pure diluted nuclei buffer. The washed isolated nuclei were then resuspended in 7–10 μL diluted nuclei buffer and were directly added to the transposition reaction after quality control and counting. In all following steps, 10X Genomics’ Single Cell Multiome ATAC and gene-expression protocols (CG000338) were followed according to the manufacturer’s specifications and guidelines. The final libraries were sequenced on the Illumina NovaSeq 6000 platform following the recommendations from 10X Genomics. Raw reads were aligned to the human genome (GRCh38) and pre-processed using the 10X Genomics CellRangerARC pipeline (v 2.0.0) for downstream analyses.

### Preprocessing of 10X multiome raw data

Multiome data was pre-processed using Scanpy^84^ (v1.10.0) and Muon^85^ v0.1.6). ***Filtering of low-quality cells:*** First, DropletUtils^86^ (v1.22.0) was used with default parameters to estimate ambient gene expression probabilities. Next, each sample was assessed separately using standard quality control measures, and sample-specific maximum mitochondrial gene fraction, minimum number of genes per cell, minimum number of counts per cell, and maximum number of counts per cell were set to filter out low-quality cells (Source data). To further filter out cells with low ATAC-seq quality, Signac (v 1.13.0)^87^ was used to calculate ATAC-specific quality metrics. Sample-specific thresholds were identified for minimum and maximum number of counts, TSS enrichment score and nucleosome signal, as well as minimum fraction of reads in peaks, maximum fraction of fragments in the mitochondrial genome and maximum fraction of counts in blacklist (Source data). ***Doublet detection:*** We used a combination of scrublet^88^ (v0.2.3), DoubletDetection^89^ (v4.2), scds^90^ (v1.18.0), scDblFinder^91^ (v1.16.0), DoubletFinder^92^ (v2.0.4) (default parameters, expected doublet rate 0.8) and SOLO^93^ (as implemented in scvi-tools^94^ v1.1.2) to detect doublets based on the gene expression modality. In addition, scDblFinder and its implementation of AMULET^95^ were used to identify doublets on the ATAC-seq modality. A consensus doublet score was then assigned to each cell, defined as the integer count of independent methods that classified that cell as a multiplet. ***Generation and quantification of common peak set:*** To merge the ATAC-seq data from individual samples, we followed the respective vignette on the Signac^87^ website. In brief, peaks from all samples were merged using the “reduce” function of the GenomicRanges (v1.54.1) package, and only peaks on standard chromosomes were kept. Next, for each sample, fragment counts were determined using Signac and stored, together with gene expression data in a Seurat object, which was subsequently merged into a single object. ***Normalization of ATAC-seq counts:*** Signac was used to run TF-IDF normalization on ATAC-seq counts with default parameters. The TF-IDF-normalized count matrix was then imported into Muon. ***Normalization of gene expression counts:*** Prior to normalization, data from individual samples were merged. SCTransform^96^ (v0.4.1) was used for normalization using settings vst.flavour = ”v2” and clip.range=c(-sqrt(n), sqrt(n)), where n represented the number of cells (*n* = 27585). ***Highly variable genes selection:*** The top 4000 highly variable genes were identified using the devianceFeatureSelection function from the scry package^97^ (v1.14.0) with default parameters. ***Initial annotation:*** To estimate initial cell type labels prior to integration, we calculated a weighted nearest neighbourhood graph (WNN)^98^, combining the unintegrated KNN graphs from both the RNA and ATAC modalities and performed Leiden clustering (resolution 0.9). Clusters were then annotated based on the expression of the marker genes *INS, GCG, GHRL, CCK, GAP43, SCT, SQSTM1, ARX, FEV, NEUROG3, SPINK1, LAMA1, PDGFC, MKI67*. This initial annotation was then used to provide cell type labels to scANVI^99^ in the subsequent steps. ***Multiplet removal:*** To remove putative multiplets from the dataset, a scVI model (scvi-tools v 1.1.2) was trained on the set of highly variable genes with the following hyperparameters: n_hidden=512, n_latent=50, n_layers=2, gene_likelihood = ‘nb’, dispersion = ‘gene-batch’, sample names as batch key, and the initial annotation as labels key. The resulting latent space was further refined with scANVI. Putative multiplet clusters were then pruned through an iterative procedure: (i) construction of a k-nearest-neighbour graph, (ii) Leiden community detection, and (iii) removal of any cluster in which > 95 % of cells had a consensus doublet score ≥ 1. Iterations continued until no additional multiplet clusters were detected. Finally, any remaining individual cells with a consensus doublet score ≥ 4 were excluded from the dataset. ***Integration:*** Following multiplet removal, gene-expression matrices were integrated using a second scVI model trained on the highly variable gene set (n_hidden=512, n_latent=50, n_layers=2, gene_likelihood = ‘nb’, dispersion = ‘gene-batch’) with sample names as batch key and the initial annotation as labels key, which was further refined using scANVI. Chromatin profiles were integrated with PoissonVI (scvi-tools v 1.1.2) configured with n_hidden=1024, n_latent=50, n_layers=2, using sample identifiers as the batch covariate and the transcriptome-derived initial cell type annotations as labels. To generate a joint neighbourhood graph across modalities, separate *k*-nearest-neighbour (K = 14) graphs were generated from the scANVI and PoissonVI latent embeddings and merged into a WNN graph. ***Imputation of gene expression:*** To denoise gene expression counts, Deep Count Autoencoder (DCA) (v0.3.2) was used with the following hyperparameters: batch_size=32, epochs=300, log1p=True, normalize_per_cell=False, scale=False, activation = ‘relu’, ae_type = ‘nb’, batchnorm=True, hidden_size = (1024,512,1024), optimizer = “RMSprop”. The resulting imputed matrix was subsequently log-transformed and used for visualization of gene expression patterns in UMAP and violin plots. ***Clustering and annotation:*** Clustering was performed on the WNN graph using Leiden^100^ clustering with resolution 2.5. To further separate subtypes of endocrine progenitors, the respective clusters were subclustered with lower resolutions (0.1–0.5). The resulting clusters were then annotated using a set of marker genes (Supplementary table 1). Downstream analyses were restricted to clusters corresponding to the principal endocrine and progenitor populations; two small, disconnected clusters expressing *PDGFC* and *SQSTM1* were excluded from further analyses.

### Gene regulatory network inference with Pando

***Peak-to-gene linking:*** To identify putative regulatory elements, we used the Signac (v1.9.0) LinkPeaks function with default parameters to calculate the correlation between chromatin accessibility and gene expression of nearby highly variable genes. These regions were used as candidate regions for GRN inference. ***Motif detection:*** Motifs of transcription factors, expressed in at least 10 % of the cells of any cell type, were detected in each peak using Pando’s find_motifs function and the motif collection provided by the Pando package (v1.1.1)^52^. ***Feature selection:*** We then selected the intersection between highly variable genes and transcription factors with matched motifs in the dataset as input genes for GRN inference. ***GRN inference:*** We then inferred the GRN considering peaks with a maximum distance of 5 × 10^5 bp to the TSS of an input gene using the infer_grn function with the following parameters: upstream = 5e + 05, downstream = 5e + 05, only_tss = TRUE, peak_to_gene_method = ‘Signac’, method = ‘cv.glmnet’, tf_cor = 0.1, alpha = 0.5. Next, we constructed transcription factor modules within the GRN using the find_modules function with a *p*-value threshold of 0.05, an R2 threshold of 0.1, a minimum number of variables of 10, and a minimum number of genes per module of 5. The GRN was visualized as a UMAP embedding of the TFs based on co-expression and regulatory relationship measured by the inferred coefficients, using a modified version of Pando’s plot_network_graph function. Nodes are sized by the PageRank centrality of each TF and coloured according to the enrichment of TF expression. Coverage tracks and peak-to-gene links were visualized using Signac.

### Differential gene expression analysis

We used DElegate to calculate differentially expressed genes between each cell type and all other cell types to identify marker genes, as well as between Artemether-treated and control. For the latter, we subset the data to the cell types of interest, filtered out genes detected in less than 5% of the cells, prior to differential expression analysis. We then applied individual cut-offs for log fold change and adjusted *p*-value for every comparison, given by the minimum absolute log fold change where the corresponding z-score of the absolute log fold change was greater than 0.5 and the minimum *p*value where the corresponding z-score of the *p*-value was greater than 0.25.

### Proteomics

Cell pellets of the different treatments and biological replicates (*n* = 3 or 4 per treatment group, 5 treatment groups) were resuspended in 25 µl of 2x lysis buffer (Preomics GmbH, Martinsried) prior to tryptic digest, applying the iST sample preparation kit (Preomics GmbH, Martinsried) following the manufacturer’s instructions. Briefly, the samples were lysed, reduced and alkylated for 10 min at 95 °C in lysis buffer prior to tryptic digest for 30 min at 37 °C. Digested peptides were loaded on the iST cartridge, washed, and eluted with the Elute buffer. Peptides were speed-vac dried and resuspended in different volumes of 2% acetonitrile, 0.5% TFA, for normalization of varying cell counts in the cell pellets. Equal amounts of digested peptides were measured on a QExactive HF-X mass spectrometer (ThermoFisher Scientific) online coupled to an Ultimate 3000 RSLC nano-HPLC (Dionex) as described in data-dependent acquisition mode (Grosche et al.,2016; Kaplan et al., 2022). Tryptic peptides were trapped on a C18 pre column (300 µm inner diameter (ID) × 5 mm, Acclaim PepMap100 C18, 5 µm, 100 Å, LC Packings) at 30 µl/min flow rate followed by reversed-phase separation on a C18 analytical column (nanoEase MZ HSS T3 Column, 100 Å, 1.8 µm, 75 µm × 250 mm, Waters) at 250 nl/min flow rate in a 95 min nonlinear acetonitrile gradient from 3 to 40% in 0.1% formic acid. The precursor spectrum was acquired at a resolution of 60,000 (full width at half-maximum) with a mass range from 300 to 1500 m/z with automatic gain control target set to 3 × 10E6 and a maximum injection time of 30 ms. From the MS prescan, the 15 most abundant peptide ions were selected for fragmentation (MSMS spectrum) if they were at least doubly charged, with a dynamic exclusion of 30 s. MSMS spectra were recorded at a resolution of 15,000 with automatic gain control target set to 5 × 10E2 and a maximum injection time of 50 ms. The normalized collision energy was 28.

Acquired raw files were analysed in the Proteome Discoverer software (version 2.5, Thermo Fisher Scientific) for peptide and protein identification and quantification. Database search was performed using the Sequest HT search engine against the SwissProt Human database (Release 2020_02, 20432 sequences) as described (Kaplan et al. 2022), considering full tryptic specificity, and one missed tryptic cleavage site was allowed with a minimum peptide length of 6 amino acids. Precursor mass tolerance was set to 10 ppm and fragment mass tolerance to 0.02 Da. Carbamidomethylation of C was set as a static modification. Dynamic modifications included deamidation of N and Q, oxidation of M, and a combination of Met loss with acetylation on the protein N-terminus. Peptide identifications were filtered for a Sequest XCorr threshold of 1 and a peptide false discovery rate of <1% using the Percolator node. Match between runs was enabled with 1 ppm mass tolerance and 0.5 min retention time tolerance. After normalization on total peptide amount, TOP3 unique peptide abundances were used for calculation of normalized protein abundances. Missing values were imputed by low-abundance resampling within Proteome Discoverer 2.5. Ratio calculations were based on normalized protein abundances with a max fold change of 1000. A paired ANOVA with FDR correction (adjusted *p*-values) was used as a hypothesis test. Resulting abundances, ratios, and statistical values were exported and filtered for a protein false discovery rate of <5%.

### Image quantification

For cell counting, positive cells (matching a Hoechst^+^ nucleus) were manually counted and normalized on the total number of Hoechst^+^ nuclei in all focal planes of Z-stack confocal acquisitions analysed. Nuclei were counted as Hoechst^+^ objects after applying a minimum filter and a Moments-based segmentation using Fiji.

### Statistics and reproducibility

No statistical method was used to pre-determine sample sizes, but the presented sample sizes are in line with previous publications. Statistical analysis and graphs were done using GraphPad Prism 9. Statistical methods, together with sample sizes and data representation information, are provided in the respective figure legends. Data distribution was assumed to be normal but not formally tested. Covariate analysis was not performed, in line with previous publications. Independent biological replicates from distinct differentiations were counted to determine the sample size (n).

### Reporting summary

Further information on research design is available in the Nature Portfolio Reporting Summary linked to this article.

## Supplementary information

Supplementary Information

Reporting Summary

Transparent Peer Review file

## Source data

Source Data

## Data Availability

The single-cell multiome data generated in this study have been deposited in the GEO database under accession code GSE264015. The mass spectrometry proteomics data generated in this study have been deposited in the ProteomeXchange Consortium via the PRIDE partner repository under accession code PXD064617. The remaining data generated in this study are provided in the Supplementary Information, Supplementary tables, and Source Data file. Source data are provided with this paper.

## Code availability

Scripts for single-cell multiome analysis are available at https://github.com/lickert-lab/2026_Akguen-Canan_Cozzitorto_ARX-PAX4_SC-Islets.
