# Supplementary material for: Resolving human α versus β cell fate allocation for the generation of stem cell-derived islets
Source: Nat Commun. 2026 Jul 9;17:6050. doi: 10.1038/s41467-026-75255-7 (PMC13351057; doi:10.1038/s41467-026-75255-7)
Supplement: Supplementary file 1 — Supplementary Information [file 41467_2026_75255_MOESM1_ESM.pdf]

**Resolving human α *versus* β cell fate allocation for the generation of stem cell-derived islets**

Melis Akgün Canan[1,2,3,4#], Corinna Cozzitorto[1,5#], Michael Sterr[1,2,5], Lama Saber[1,5], Eunike S.A. Setyono[1,5], Alessandro Dema[1,5], Kei Kozawa[1,5], Xianming Wang[1,5], Juliane Merl-Pham[6], Tobias Greisle[1,5], Ingo Burtscher[1,2,5], Heiko Lickert[1,2,5*]

[1]Institute of Diabetes and Regeneration Research, Helmholtz Center Munich, Neuherberg, Germany.
[2]School of Medicine, Technical University of Munich, Munich, Germany.
[3]Stem Cell Institute, Ankara University, Ankara, Türkiye.
[4]Integrated Technologies Research Center (BÜTAM), Ankara University, Ankara, Türkiye
[5]German Center for Diabetes Research (DZD), Neuherberg, Germany.
[6]Metabolomics and Proteomics Core, Helmholtz Center Munich, Heidemannstr, Germany.

## SUPPLEMENTARY INFORMATION FIGURES 1-9

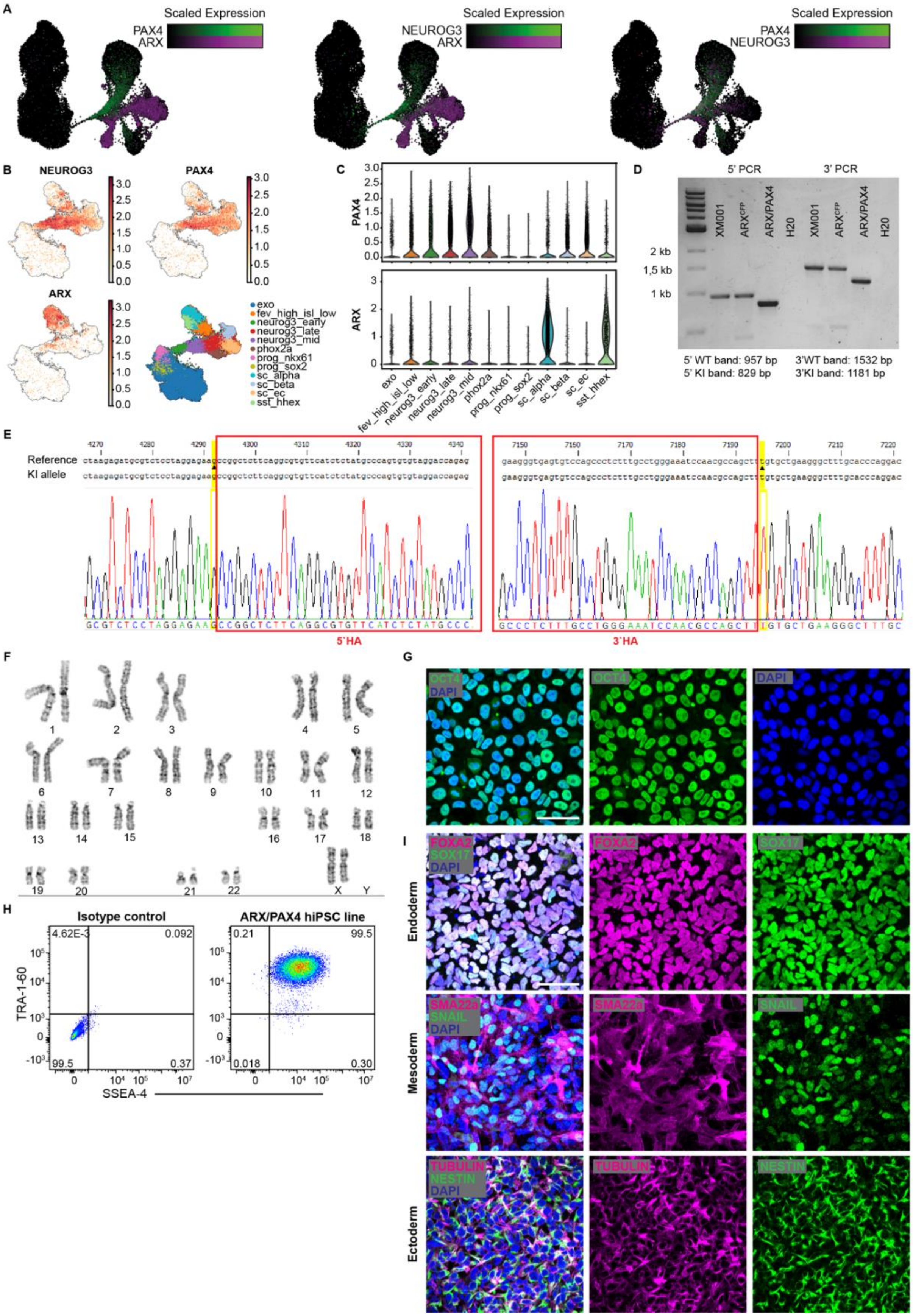

A
Scaled Expression
PAX4
ARX
NEUROG3
B
NEUROG3
PAX4
ARX
exo
fev_high_isl_low
neurog3_early
neurog3_late
neurog3_mid
phox2a
prog_nkx61
prog_sox2
sc_alpha
sc_beta
sc_ec
sst_hhex
C
PAX4
ARX
D
5' PCR
3' PCR
XM001
ARX/PAX4
H20
2 kb
1,5 kb
1 kb
5' WT band: 957 bp
5' KI band: 829 bp
3'WT band: 1532 bp
3'KI band: 1181 bp
E
Reference
KI allele
5`HA
3`HA
F
X
Y
G
OCT4
DAPI
H
Isotype control
ARX/PAX4 hiPSC line
TRA-1-60
SSEA-4
I
FOXA2
SOX17
SMA22a
SNAIL
TUBULIN
NESTIN
Endoderm
Mesoderm
Ectoderm

***Supplementary Fig. 1. Generation and quality controls of a human ARX$^{CFP/CFP}$/PAX4$^{mCherry/mCherry}$ reporter iPSC line.*** **(A)** UMAP showing co-expression of *ARX* and *PAX4* in human foetal pancreas as in Fig. 1B. **(B-C)** UMAPs and violin plots of *NEUROG3, PAX4* and *ARX* expression in human SC-islets at differentiation stage 5 from the publicly available dataset from Veres *et al.*, 2019. **(D)** Genotyping of indicated iPSC clones for 5´ and 3´regions spanning the homology arms after transfection, sorting, and single-cell clonal culture. **(E)** Sanger sequencing of the 3' and 5' recombination boarders of the knock-in (KI) allele including the homology arms (HA). **(F)** Unperturbed female karyotype (46, XX) **(G-H)** Assessment of pluripotency using the nuclear markers OCT3/4 by IF (F), and the surface markers TRA-1-60 and SSEA-4 by flow cytometry analysis (G). **(I)** Direct differentiation toward the three germ layers (endoderm [FOXA2, SOX17], mesoderm [SM22-a, SNAIL] and ectoderm [TUBB3, NESTIN]) show full pluripotency and multilineage differentiation potential of selected clone. Scale bars 50 µm.

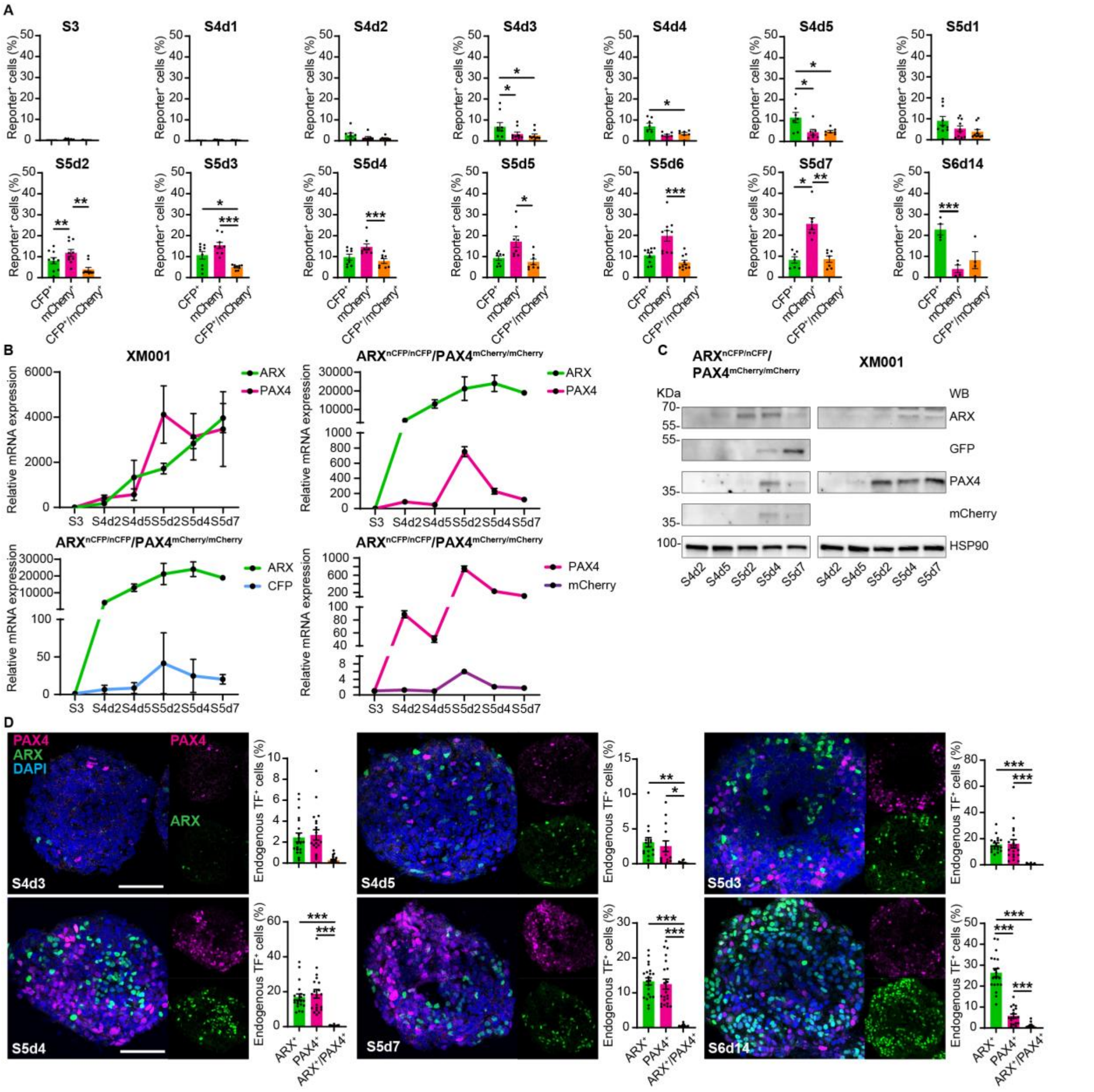

***Supplementary Fig. 2. Comparison of TFs dynamics in ARX/PAX4 cell line and its parental counterpart.*** **(A)** Flow cytometry analysis of the percentage of ARX-CFP and PAX4-mCherry positive cells starting from S3 (early PP stage) to S6d14 (SC-β cells); n=8, 9, 8, 9, 7, 7, 10, 10, 9, 8, 8, 10, 7, 4 from distinct differentiations, mean ± SE, repeated measures one-way ANOVA followed by Tukey's post-test. **(B-C)** Bulk analysis of mRNA and protein levels of endogenous TFs in both the ARX/PAX4 and its parental line (XM001) and their reporters in the ARX/PAX4 cell line at the indicated stages. n=3 from distinct differentiations each. **(D)** Representative maximum intensity projections of Z-stack confocal acquisitions and their quantifications of IF for endogenous ARX and PAX4 in wild-type clusters at the indicated stages. n=3 biological replicates from distinct differentiations; in D each dot represents a single technical replicate (cells cluster) from all 3 biological replicates, mean ± SE, repeated measures one-way ANOVA followed by Tukey's post-test.

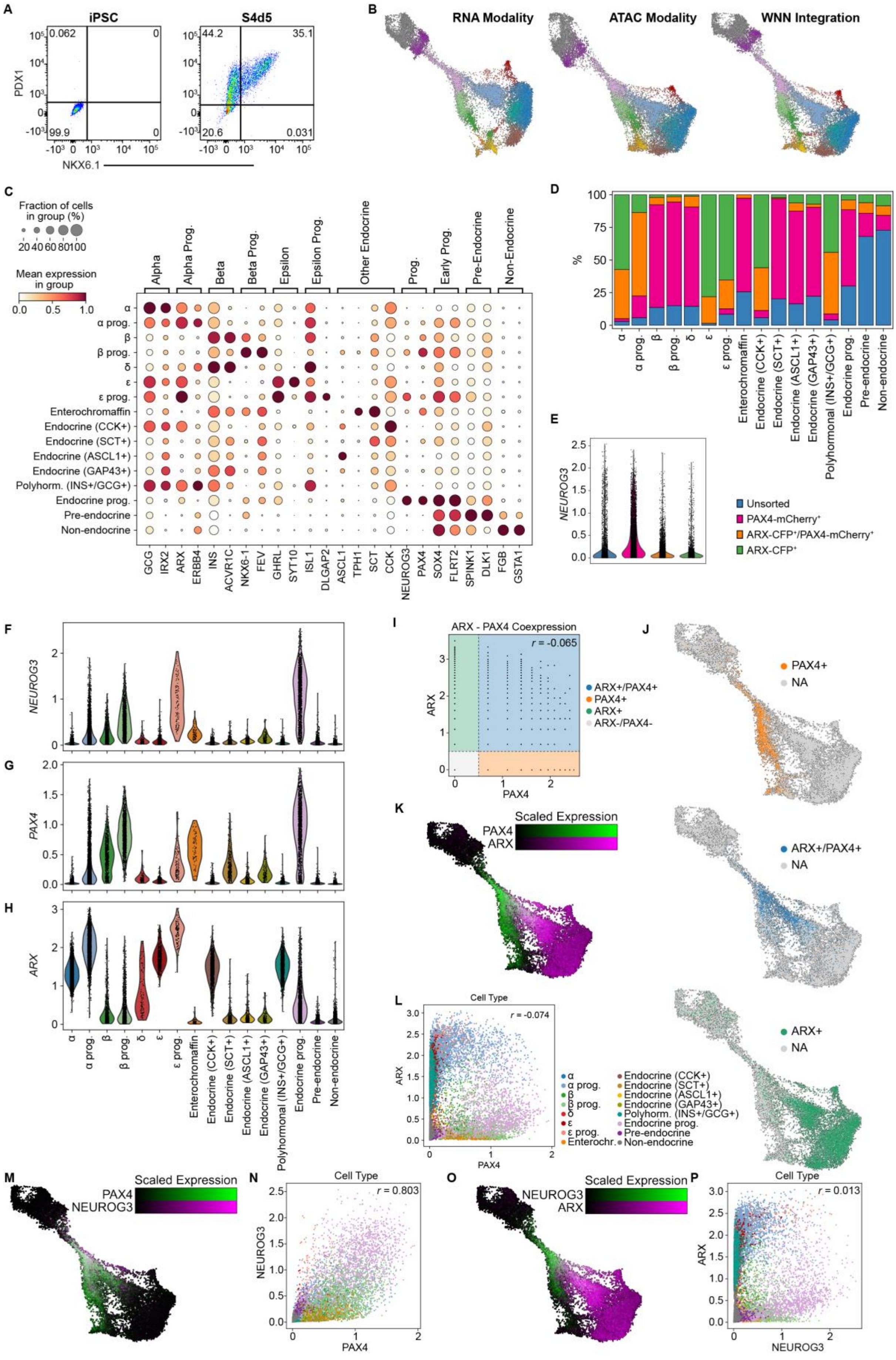

A
iPSC
S4d5
PDX1
NKX6.1
0.062
0
99.9
44.2
35.1
20.6
0.031
B
RNA Modality
ATAC Modality
WNN Integration
C
Fraction of cells in group (%)
20 40 60 80 100
Mean expression in group
0.0 0.5 1.0
Alpha
Alpha Prog.
Beta
Beta Prog.
Epsilon
Epsilon Prog.
Other Endocrine
Prog.
Early Prog.
Pre-Endocrine
Non-Endocrine
α
α prog.
β
β prog.
δ
ε
ε prog.
Enterochromaffin
Endocrine (CCK+)
Endocrine (SCT+)
Endocrine (ASCL1+)
Endocrine (GAP43+)
Polyhorm. (INS+/GCG+)
Endocrine prog.
Pre-endocrine
Non-endocrine
GCG IRX2 ARX ERBB4 INS ACVR1C NKX6-1 FEV GHRL SYT10 ISL1 DLGAP2 ASCL1 TPH1 SCT CCK NEUROG3 PAX4 SOX4 FLRT2 SPINK1 DLK1 FGB GSTA1
D
%
Polyhormonal (INS+/GCG+)
Unsorted
PAX4-mCherry⁺
ARX-CFP⁺/PAX4-mCherry⁺
ARX-CFP⁺
E
NEUROG3
F
NEUROG3
G
PAX4
H
ARX
I
ARX - PAX4 Coexpression
r = -0.065
ARX+/PAX4+
PAX4+
ARX+
ARX-/PAX4-
J
PAX4+
NA
ARX+/PAX4+
NA
ARX+
NA
K
Scaled Expression
PAX4
ARX
L
Cell Type
r = -0.074
Enterochr.
Polyhorm. (INS+/GCG+)
M
Scaled Expression
PAX4
NEUROG3
N
Cell Type
r = 0.803
O
Scaled Expression
NEUROG3
ARX
P
Cell Type
r = 0.013

***Supplementary Fig. 3. Single cells multiomics suggests ARX repression of PAX4 during in vitro SC-β cells differentiation.*** **(A)** Flow cytometry plots showing differentiation efficiency (percentage of cells positive for PDX1 and NKX6.1) at S4d5 of samples subsequently submitted for scRNA-Seq and snATAC-Seq at S5d4 (S4d5) and iPSC controls. **(B)** UMAP embedding of single cells RNA-Sequencing (left), single cells ATAC-Sequencing (middle) and the integration of both (right) for all datasets. **(C)** Expression levels of annotated genes within all clusters. The color assigned to each gene in each cluster represents the average gene expression level, while the size of each circle represents the percentage of positive cells for each gene in that cluster. **(D)** Relative frequencies of cell types in each dataset as per annotated UMAP in Fig. 2B. **(E)** Violin plot showing *NEUROG3* within all datasets. **(F-H)** Violin plots showing *NEUROG3, PAX4 and ARX* expression within all clusters **(I)** Scatter plot showing log normalized counts of *ARX* and *PAX4* and the thresholds for classification of *ARX*$^+$ and *PAX4*$^+$ cells. Pearson correlation coefficient r of log normalized counts is shown. **(J)** UMAP showing *PAX4*$^+$, *ARX*$^+$*/PAX4*$^+$, and *ARX*$^+$ cell populations. **(K, M, O)** UMAPs showing co-expression of *ARX* and *PAX4* (K), *PAX4* and *NEUROG3* (M), and *ARX* and *NEUROG3* (O). **(L, N, P)** Scatter plots showing log imputed counts of *ARX* and *PAX4* (L), *PAX4* and *NEUROG3* (N), and *ARX* and *NEUROG3* (P). Pearson correlation coefficients r of log imputed counts are shown.

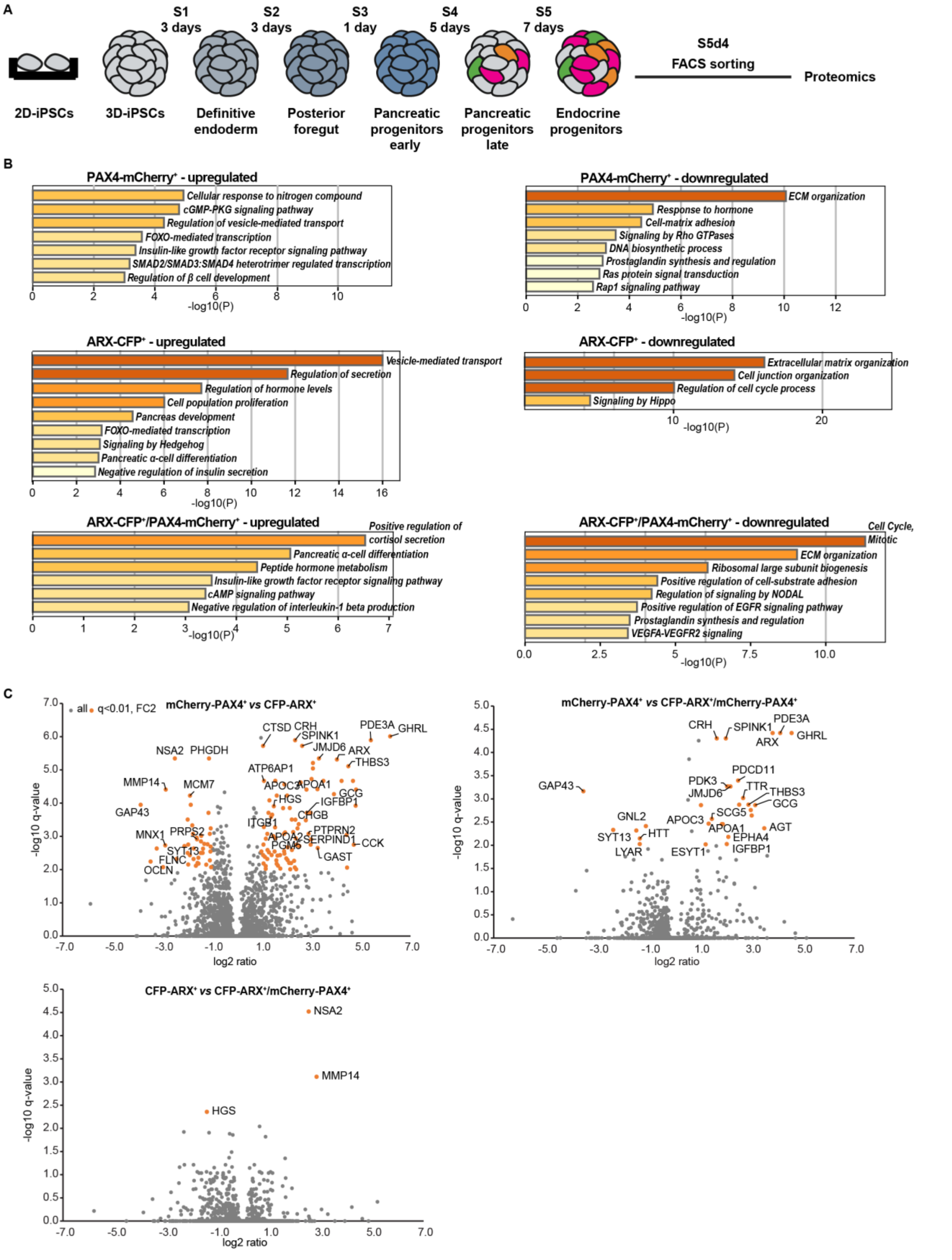


***Supplementary Fig. 4. Proteomics analysis confirms multiomic analysis.*** **(A)** Experimental

strategy for proteomics analysis. **(B)** Ontology analysis of significant differentially expressed proteins for indicated samples against unsorted sample. **(C)** Volcano plots showing differentially expressed proteins between the specified samples. Note the small differences between the CFP-ARX$^+$ and the CFP-ARX$^+$/mCherry-PAX4$^+$ samples. Proteins with a Foldchange of >2 fold (log2<-1 or log2>1) and a q-value >0.01 are represented by orange dots. q-values: Benjamini-Hochberg corrected p-values.

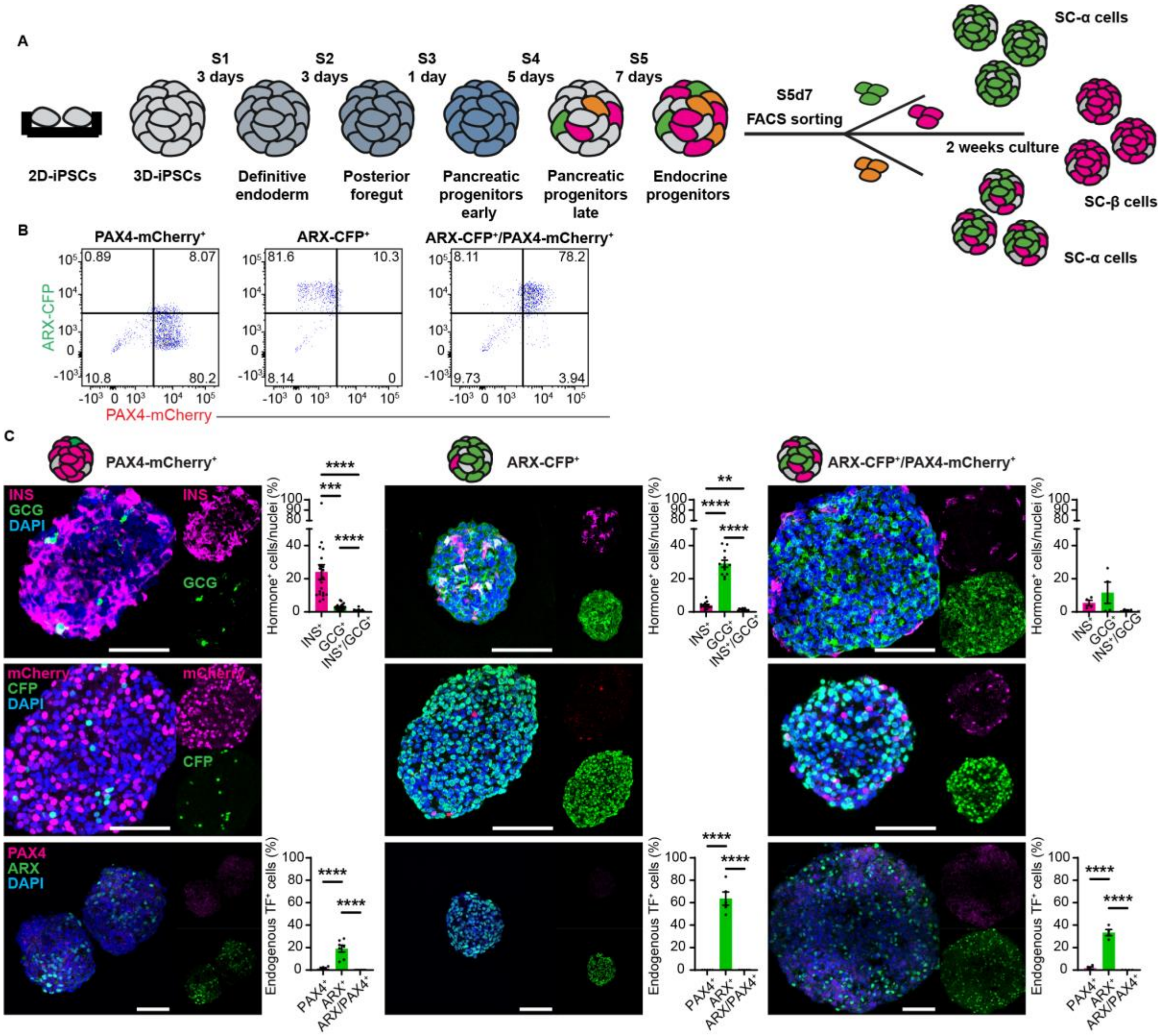


***Supplementary Fig. 5. ARX expression coordinates α versus β cell fate decision.*** **(A)** Experimental strategy for potency assay of ARX-CFP⁺, ARX-CFP⁺/PAX4-mCherry⁺ and PAX4-mCherry⁺ FACS-sorted fractions at S5d7. **(B)** Representative flow cytometry plots of fractions purity directly after sorting. **(C)** Representative maximum intensity projections of Z-stack confocal acquisitions of IF, and related quantifications, on FACS-sorted ARX-CFP⁺, ARX-CFP⁺/PAX4-mCherry⁺ and PAX4-mCherry⁺ fractions-derived clusters showing insulin and glucagon, ARX-CFP and PAX4-mCherry, and endogenous PAX4 and ARX after 2 weeks in culture. n=3 biological replicates from distinct differentiations, each dot represents a single technical replicate (cells cluster) from all 3 biological replicates. INS: insulin, GCG: glucagon. Data are presented as mean ± SE. One-way ANOVA with Tukey multiple comparison test. Scale bars: 50 µm

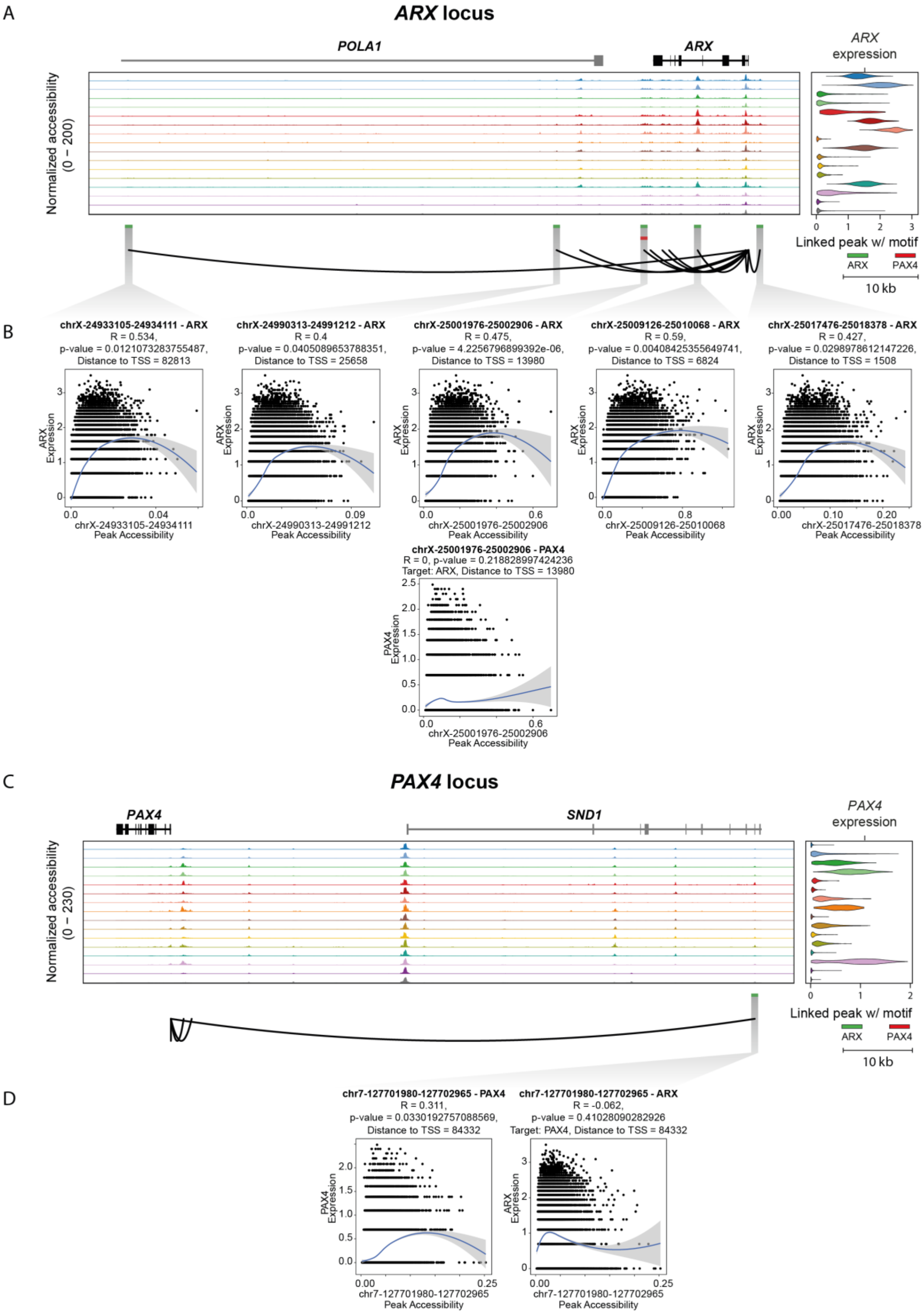
A
ARX locus
POLA1
ARX
ARX expression
Normalized accessibility (0 – 200)
Linked peak w/ motif
ARX
PAX4
10 kb
B
chrX-24933105-24934111 - ARX
R = 0.534,
p-value = 0.0121073283755487,
Distance to TSS = 82813
chrX-24990313-24991212 - ARX
R = 0.4
p-value = 0.0405089653788351,
Distance to TSS = 25658
chrX-25001976-25002906 - ARX
R = 0.475,
p-value = 4.2256796899392e-06,
Distance to TSS = 13980
chrX-25009126-25010068 - ARX
R = 0.59,
p-value = 0.00408425355649741,
Distance to TSS = 6824
chrX-25017476-25018378 - ARX
R = 0.427,
p-value = 0.0298978612147226,
Distance to TSS = 1508
ARX Expression
chrX-24933105-24934111 Peak Accessibility
chrX-24990313-24991212 Peak Accessibility
chrX-25001976-25002906 Peak Accessibility
chrX-25009126-25010068 Peak Accessibility
chrX-25017476-25018378 Peak Accessibility
chrX-25001976-25002906 - PAX4
R = 0, p-value = 0.218828997424236
Target: ARX, Distance to TSS = 13980
PAX4 Expression
chrX-25001976-25002906 Peak Accessibility
C
PAX4 locus
PAX4
SND1
PAX4 expression
Normalized accessibility (0 – 230)
Linked peak w/ motif
ARX
PAX4
10 kb
D
chr7-127701980-127702965 - PAX4
R = 0.311,
p-value = 0.0330192757088569,
Distance to TSS = 84332
chr7-127701980-127702965 - ARX
R = -0.062,
p-value = 0.41028090282926
Target: PAX4, Distance to TSS = 84332
PAX4 Expression
ARX Expression
chr7-127701980-127702965 Peak Accessibility

***Supplementary Fig. 6. Cis-regulatory elements of the ARX and PAX4 loci with ARX and PAX4 motifs.*** **(A, C)** Accessibility and expression of the *ARX* (A) and *PAX4* (C) locus. Peaks with significant accessibility to gene expression correlation within a 100 kb region around the TSS are depicted by arcs. Peaks with ARX or PAX4 motif are marked in green and red, respectively. **(B, D)** Scatterplots showing the accessibility of each peak with ARX or PAX4 motif against the expression of *ARX* (for peaks with ARX motif) or *PAX4* (for peaks PAX4 motif) gene expression. Pearson correlation coefficients, p-values and the exact distance to the TSS are shown.

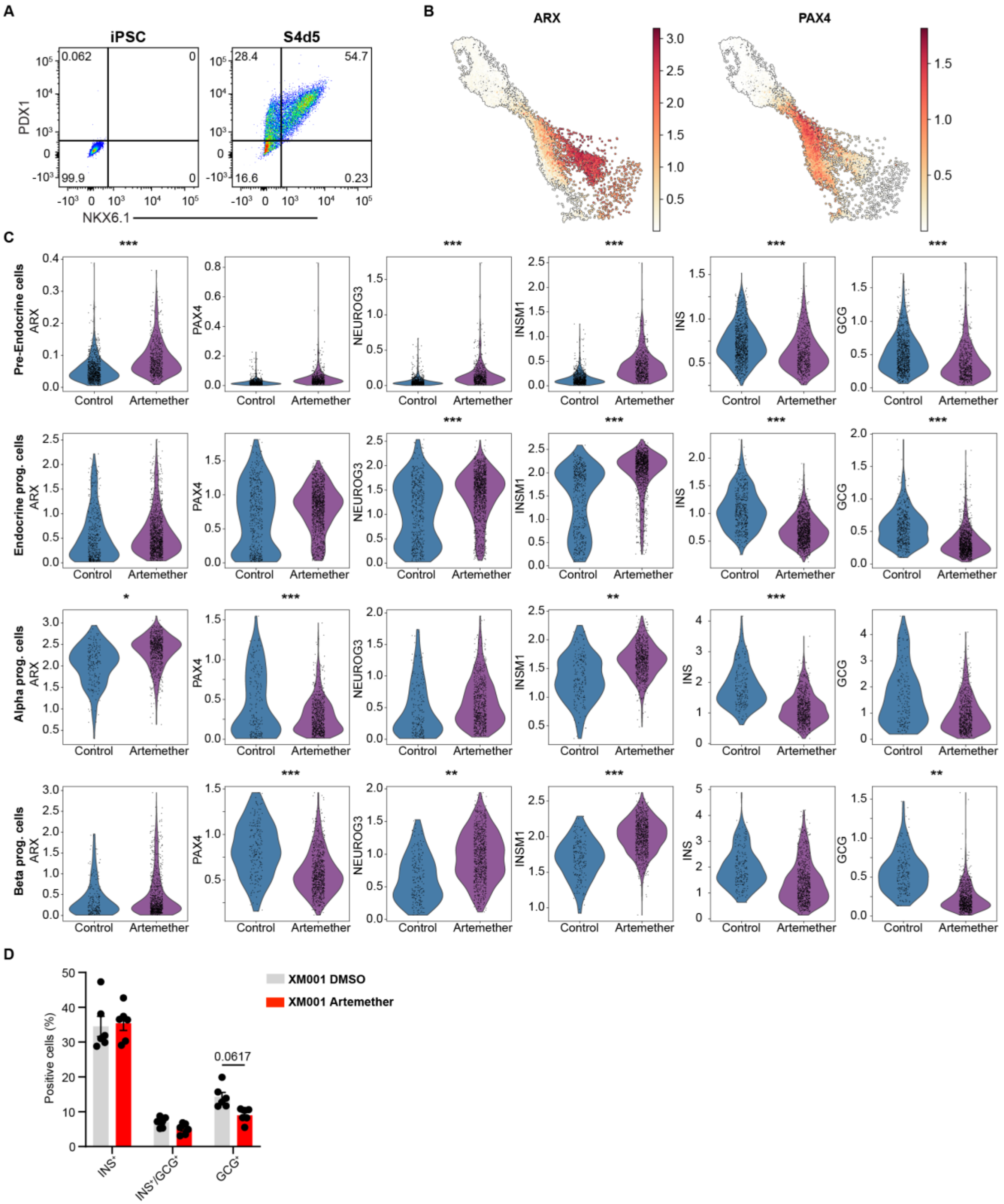


***Supplementary Fig. 7. Artemether promotes endocrine induction.*** **(A)** Flow cytometry plots showing differentiation efficiency (percentage of PDX1$^{+}$/NKX6.1$^{+}$ cells) at S4d5 of artemether-treated samples subsequently submitted for scRNA-Seq and snATAC-Seq at S5d4 (S4d5) and iPSC controls. **(B)** UMAPs showing *PAX4* and *ARX* expression at S5d4 after artemether treatment. **(C)** Violin plots showing indicated genes expression within specified clusters of DMSO- and artemether-treated datasets at S5d4 **(D)** Quantification of hormone-positive cells at S6d14 after continuous treatment,

with artemether starting from S4 of wild-type hIPSC (XM001). Data are presented as mean ± SE, n=6. Two-ways ANOVA with Šídák multiple comparison test.

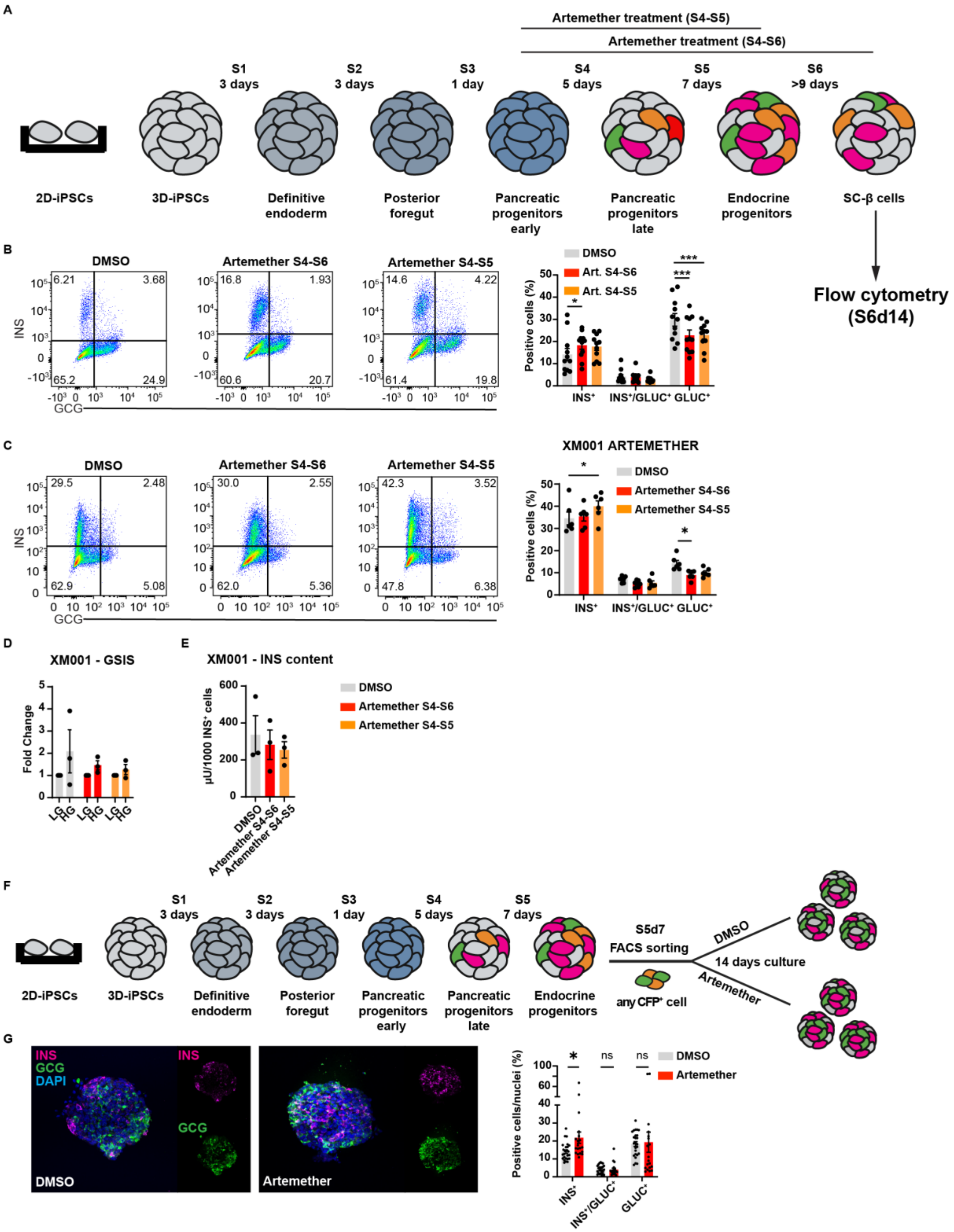


***Supplementary Fig. 8. Artemether has a profound effect on endocrine lineage induction and segregation but not on insulin secretion or α-to-β cell transdifferentiation.*** **(A)** Experimental strategy for short (S4-S5) and long (S4 to S6) artemether treatment followed by flow cytometry analysis at S6d14. **(B-C)** Representative flow cytometry plots and related quantification of the

percentage of hormones-positive cells in ARX/PAX4 clusters (C) or wild type cells (XM001 – D) at S6d14 after indicated artemether treatments; n=11, 6. **(D-E)** Static insulin secretion of SC-islets treated as indicated in a GSIS assay (D) and related insulin content (E). n=3 biological replicates from distinct differentiations **(F)** Experimental strategy for assessment of α-to-β transdifferentiation after artemether treatment. We sorted any $CFP^+$ cell (whether ARX-$CFP^+$ or PAX4-$mCherry^+$/ ARX-$CFP^+$) at S5d7 and then culture them for 2 weeks with artemether or DMSO. **(G)** Representative maximum intensity projections of Z-stack confocal acquisitions of IF staining, and respective quantification, showing insulin and glucagon hormones within artemether-treated and DMSO-treated control clusters at S6d14; n=3 biological replicates from distinct differentiations, each dot represents a single technical replicate (cells cluster) from all 3 biological replicates. **(B-G)** INS: insulin, GCG: glucagon, LG: low glucose, HG: high glucose. Data are presented as mean ± SE, two-ways ANOVA with Šídák multiple comparison test. Scale bars: 50 µm.

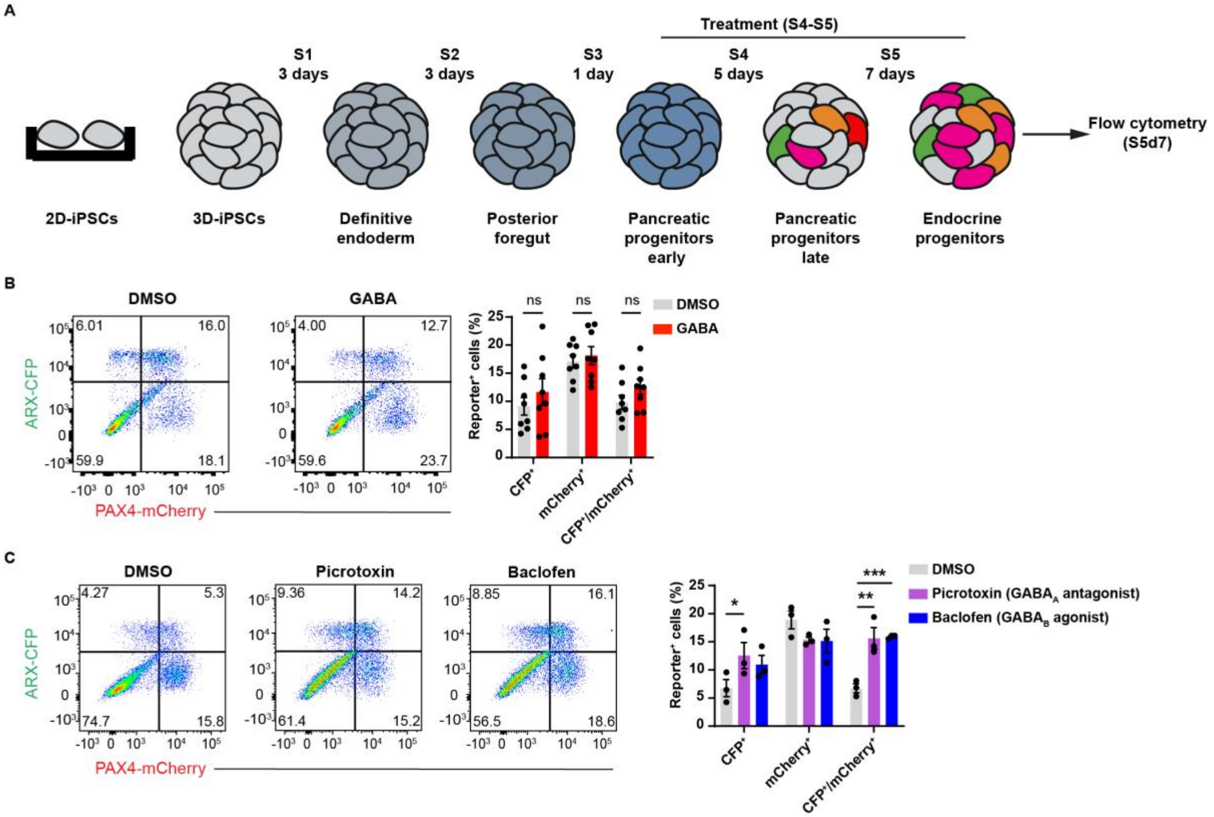


***Supplementary Fig. 9. Modulation of GABA signaling does not mimic artemether treatment.*** **(A)** Experimental strategy for modulation of GABA signalling during S4 and S5 followed by flow cytometry analysis at S5d7. **(B-C)** Representative flow cytometry plots and related quantifications of the percentage of ARX-CFP$^{+}$, ARX-CFP$^{+}$/PAX4-mCherry$^{+}$ and PAX4-mCherry$^{+}$ cells at S5d7 after indicated treatments. Data are presented as mean ± SE; n=8, 3 biological replicates from distinct differentiations. Two-ways ANOVA with Šídák multiple comparison test; ns: not significant.

**Resolving human α *versus* β cell fate allocation for the generation of stem cell-derived islets**

Melis Akgün Canan[1,2,3,4#], Corinna Cozzitorto[1,5#], Michael Sterr[1,2,5], Lama Saber[1,5], Eunike S.A. Setyono[1,5], Alessandro Dema[1,5], Kei Kozawa[1,5], Xianming Wang[1,5], Juliane Merl-Pham[6], Tobias Greisle[1,5], Ingo Burtscher[1,2,5], Heiko Lickert[1,2,5*]

[1]Institute of Diabetes and Regeneration Research, Helmholtz Center Munich, Neuherberg, Germany.
[2]School of Medicine, Technical University of Munich, Munich, Germany.
[3]Stem Cell Institute, Ankara University, Ankara, Türkiye.
[4]Integrated Technologies Research Center (BÜTAM), Ankara University, Ankara, Türkiye
[5]German Center for Diabetes Research (DZD), Neuherberg, Germany.
[6]Metabolomics and Proteomics Core, Helmholtz Center Munich, Heidemannstr, Germany.

[#]These authors contributed equally: Melis Akgün Canan, Corinna Cozzitorto
*Corresponding author's email: heiko.lickert@helmholtz-munich.de

**SUPPLEMENTARY TABLES 1-7**

## Supplementary table 1. Primary antibody list

| Name | Host/ Conjugated | Company | Order number | Dil. IHC | Dil. FACS | Dil. WB | Lot Number |
|---|---|---|---|---|---|---|---|
| **ARX** | Sheep | R&D Systems | AF7068 | 1:100 | | 1:1000 | CFOM0217021 |
| **Caspase-3, Cleaved** | Rabbit | Cell Signaling | 9661S | 1:100 | 1:100 | | 42 |
| **FOXA2 (HNF-3β)** | Rabbit | Cell Signaling | 8186S | 1:300 | 1:200 | | AB_10891055 |
| **GFP** | Chicken | Aves Labs | GFP-1020 | 1:300 | 1:300 | | 1223FP03 |
| **GFP** | Mouse | Abclonal | ABIN3020573 | | | 1:2000 | 0000541687 |
| **Glucagon** | Mouse | Merck, Sigma-Aldrich | G2654-.2ML | 1:1000 | 1:500 | | 04061833629314 |
| **Glucagon** | FITC | Novus Biologicals | NBP2-21803P | | 1:180 | | |
| **HSP90** | Rabbit | Cell signaling | 4874S | | | 1:5000 | D5D9Z |
| **Insulin** | Guinea pig | Bio-Rad | 5330-0104G | 1:500 | 1:500 | | |
| **Insulin-T56-706** | APC | BD | 565689 | | 1:40 | | 2168160 |
| **Ki-67** | Rabbit | Abcam | ab15580 | 1:300 | 1:500 | | GR45436-1 |
| **NEUROG3** | Rabbit | Aviva Systems Biology | OACD05949 | 1:200 | 1:100 | | |
| **NESTIN (10C2)** | Mouse | Abcam | ab22035 | 1:200 | | | 446723 |
| **NKX2-2** | Mouse | Abcam | ab187375-500ul | 1:300 | 1:200 | | GR299445-2 |
| **NKX6-1** | Rabbit | Bio-techne, Novus | NBP1-82553 | 1:300 | 1:200 | | |
| **Oct-3/4** | Goat | Santa Cruz | sc-8628 | 1:500 | | | 3170808 |
| **PDX1** | Goat | R&D Systems | AF2419 | 1:300 | 1:100 | | |
| **PAX4** | Rabbit | Life Technologies | PA1-108 | 1:200 | | 1:1000 | 2407885 |
| **RFP (5F8)** | Rat | chromotek | ORD003515 | 1:300 | 1:300 | | |
| **RFP-TagRFP** | Rabbit | Biocat/Evrogen | AB234 | | | 1:3000 | |
| **SOX17** | Goat | Neuromics | GT15094 | 1:400 | 1:200 | | |
| **SLC18A** | Rabbit | Atlas Antibodies | ATAHPA063797-100 | | 1:200 | | |
| **Snail** | Goat | R&D Systems | AF3639 | 1:300 | | | 5036-WN-010 |
| **Tubulin beta III** | Rabbit | Abcam | ab18207 | 1:1000 | | | 1056145-1 |

**Supplementary table 2. Secondary antibody list**

| Name | Host | Fluorophore/Conjugate | Company | Order number | Dilution |
|---|---|---|---|---|---|
| **anti-Guinea Pig IgG** | Donkey | AlexaFluor 488 | Biozol, Jackson ImmunoResearch | 706-545-148 | 1:500 |
| **anti-Guinea Pig IgG** | Donkey | AlexaFluor 647 | Biozol, Jackson ImmunoResearch | 706-165-148 | 1:500 |
| **anti-Goat IgG** | Donkey | AlexaFluor 488 | Thermo Fisher Scientific, Invitrogen | A11055 | 1:500 |
| **anti-Goat IgG** | Donkey | AlexaFluor 555 | Thermo Fisher Scientific, Invitrogen | A21432 | 1:500 |
| **anti-Goat** | Donkey | AlexaFluor 647 | Biozol, Jackson ImmunoResearch | 705-605-147 | 1:500 |
| **anti-Mouse IgG** | Donkey | AlexaFluor 488 | Thermo Fisher Scientific, Invitrogen | A21202 | 1:500 |
| **anti-Mouse IgG** | Donkey | AlexaFluor 555 | Thermo Fisher Scientific, Invitrogen | A31570 | 1:500 |
| **anti-Mouse** | Donkey | AlexaFluor 647 | Thermo Fisher Scientific, Invitrogen | 715-605-151 | 1:500 |
| **anti-Rabbit IgG** | Donkey | AlexaFluor 488 | Thermo Fisher Scientific, Invitrogen | A21206 | 1:500 |
| **anti-Rabbit IgG** | Donkey | AlexaFluor 555 | Thermo Fisher Scientific, Invitrogen | A31572 | 1:500 |
| **anti-Rabbit IgG** | Donkey | AlexaFluor 647 | Thermo Fisher Scientific, Invitrogen | A31573 | 1:500 |
| **anti-Rat IgG** | Donkey | Cy3 | Biozol, Jackson ImmunoResearch | 712-165-153 | 1:500 |
| **anti-Sheep IgG** | Donkey | AlexaFluor 488 | Biozol, Jackson ImmunoResearch | 713-546-147 | 1:500 |
| **anti-Sheep IgG, Fc fragm. specific** | Rabbit | HRP | Biozol, Jackson ImmunoResearch | 313-035-046 | 1:5000 |
| **anti-Rabbit IgG (H+L)** | Goat | HRP | Dianova/Jackson | 111-035-144 | 1:5000 |
| **anti-Mouse IgG (H+L)** | Goat | HRP | Dianova/Jackson | 115-035-146 | 1:5000 |

## Supplementary table 3. PAX4 single guide RNA

| PAX4-sgRNA | Sequence |
|---|---|
| **Forward PAX4-sgRNA** | CACC GGG GAGCATGCATCAGGACGGTG |
| **Reverse PAX4-sgRNA** | AAACCACCGTCCTGATGCATGCTC CCC |
| **Kozak sequence** | CACC |
| **PAM sequence** | 5'-NGG-3' |

## Supplementary table 4. Primer for genotyping

| Primer | Sequence | Primer number |
|---|---|---|
| **Pax4 3' KI forward primer** | GGATCACTCTCGGCATGGAC | EP308 |
| **Pax4 3'KI reverse primer** | TCTGAGGGCTTCTGGGACTTGG | EP1849 |
| **Pax4 5'KI forward primer** | CAGCAGGTTAGAGATGCTAAGAG | EP408 |
| **Pax4 5'KI reverse primer** | GCCTTCTCAGCCCTGGAAGACAC | EP1848 |

## Supplementary table 5. Primer for cloning homology arms of Pax4

| Primer | Sequence with overhangs | Tm |
|---|---|---|
| **5'HA Pax4 forward** | CTATAGGGCGAATTGGAGCTCCACCGC GCCGGCTCTTCAGGCGTGTTCATCTC | 73ºC |
| **5'HA Pax4 reverse** | CTTCGCTGGCTCTGGCATGGTGGCGC GGCTGACCCTCCTCAGAAGGATGAGAC | 71ºC |
| **3'HA Pax4 forward** | GCGACGTTGAGGAAAACCCAGGACCAATGCATCAGGACGGTGAGGAGCCTGGG | 74ºC |
| **3'HA Pax4 forward** | CCCCTCGAGGTCGACGGTATCGATA GCTGGCGTTGGATTTCCCAGGCAAAGAGG | 75ºC |

| Supplementary table 6. Kit | Company | Catalogue number |
|---|---|---|
| **Gel Extraction Kit** | Qiagen | 28704 |
| **PCR Purification Kit** | Qiagen | 28104 |
| **Plasmid Mini Kit** | Qiagen | 12125 |
| **Plasmid PLUS Midi Kit** | Qiagen | 12943 |
| **Insulin ELISA** | Mercodia | 10-1113-01 |

## Supplementary table 7. qPCR probes

| Gene | TaqMan® Primer Prob ID |
|---|---|
| **GAPDH** | Hs02758991_g1 |
| **PAX4** | Hs00173014_m1 |
| **ARX** | Hs002292465_m1 |
| **mCherry/mORANGE** | Mr07319438_mr |
| **CFP/GFP** | mr04329676_mr |
