# Supplementary material for: Resolving human α versus β cell fate allocation for the generation of stem cell-derived islets
Source: Nat Commun. 2026 Jul 9;17:6050. doi: 10.1038/s41467-026-75255-7 (PMC13351057; doi:10.1038/s41467-026-75255-7)
Supplement: Supplementary file 2 — Reporting Summary [file 41467_2026_75255_MOESM2_ESM.pdf]

nature portfolio

Corresponding author(s): Heiko Lickert

Last updated by author(s): Apr 27, 2026

Reporting Summary

Nature Portfolio wishes to improve the reproducibility of the work that we publish. This form provides structure for consistency and transparency in reporting. For further information on Nature Portfolio policies, see our Editorial Policies and the Editorial Policy Checklist.

Statistics

For all statistical analyses, confirm that the following items are present in the figure legend, table legend, main text, or Methods section.

n/a	Confirmed	
☐	☒	The exact sample size (*n*) for each experimental group/condition, given as a discrete number and unit of measurement
☐	☒	A statement on whether measurements were taken from distinct samples or whether the same sample was measured repeatedly
☐	☒	The statistical test(s) used AND whether they are one- or two-sided<br>*Only common tests should be described solely by name; describe more complex techniques in the Methods section.*
☐	☒	A description of all covariates tested
☐	☒	A description of any assumptions or corrections, such as tests of normality and adjustment for multiple comparisons
☐	☒	A full description of the statistical parameters including central tendency (e.g. means) or other basic estimates (e.g. regression coefficient) AND variation (e.g. standard deviation) or associated estimates of uncertainty (e.g. confidence intervals)
☐	☒	For null hypothesis testing, the test statistic (e.g. *F*, *t*, *r*) with confidence intervals, effect sizes, degrees of freedom and *P* value noted<br>*Give P values as exact values whenever suitable.*
☒	☐	For Bayesian analysis, information on the choice of priors and Markov chain Monte Carlo settings
☒	☐	For hierarchical and complex designs, identification of the appropriate level for tests and full reporting of outcomes
☒	☐	Estimates of effect sizes (e.g. Cohen's *d*, Pearson's *r*), indicating how they were calculated

Our web collection on statistics for biologists contains articles on many of the points above.

Software and code

Policy information about availability of computer code

Data collection	Flow cytometry and sorting: BD FACS Aria III.<br>Confocal microscopy: Zeiss LSM 880 Airy Scan.<br>qPCR: ViiA7, 96 well/Array card (Applied Bioscience)<br>scMultiomics: Chromium Controller (10X Genomics)
Data analysis	FACS and flow cytometry analysis: FlowJo v10.7.1.<br>Image analyses: Zeiss Zen 3.4 Lite Blue, ImageJ (1.54p)/Fiji (NIH).<br>Statistical analyses: GraphPad Prism software 10.0.3 (La Jolla, CA).<br>Sanger sequencing anaylsis: Chromas 2.6.6<br>scMultiomics analysis: Scanpy v1.10.0, Muon v0.1.6, DropletUtils v1.22.0, Signac v 1.9.0/1.13.0, scrublet v0.2.3, DoubletDetection v4.2, scds v1.18.0, scDblFinder v1.16.0, DoubletFinder v2.0.4, scvi-tools v1.1.2, GenomicRanges v1.54.1, SCTransform v0.4.1, scry v1.14.0, Deep Count Autoencoder (DCA) v0.3.2, Pando v1.1.1, DElegate v 1.2.1, edgeR v4.0.16

For manuscripts utilizing custom algorithms or software that are central to the research but not yet described in published literature, software must be made available to editors and reviewers. We strongly encourage code deposition in a community repository (e.g. GitHub). See the Nature Portfolio guidelines for submitting code & software for further information.

April 2023

Data

Policy information about availability of data

All manuscripts must include a data availability statement. This statement should provide the following information, where applicable:

- Accession codes, unique identifiers, or web links for publicly available datasets
- A description of any restrictions on data availability
- For clinical datasets or third party data, please ensure that the statement adheres to our policy

The single-cell multiome data generated in this study have been deposited in the GEO database under accession code GSE264015 [https://www.ncbi.nlm.nih.gov/geo/query/acc.cgi?acc=GSE264015].
The mass spectrometry proteomics data generated in this study have been deposited in the ProteomeXchange Consortium via the PRIDE partner repository under accession code PXD064617 [https://www.ebi.ac.uk/pride/archive/projects/PXD064617].
The remaining data generated in this study are provided in the Supplementary Information, Supplementary Data, and Source Data file.

Research involving human participants, their data, or biological material

Policy information about studies with human participants or human data. See also policy information about sex, gender (identity/presentation), and sexual orientation and race, ethnicity and racism.

Reporting on sex and gender	The human iPSC line described in this study is derived from an already published iPSC line (HMGUi001-A) derived from a female donor (Wang et al., 2018)
Reporting on race, ethnicity, or other socially relevant groupings	Caucasian
Population characteristics	N/A
Recruitment	No human subjects were recruited for this study
Ethics oversight	The Ethics Committee of the Technical University Munich positively voted on non-commercial research on human iPS cells (219/20 S). The donor of the original material expressed his/her informed consent to the generation of iPSCs from fibroblasts and further research activities. The material was donated under an epidemiological study at the University hospital Tübingen.

Note that full information on the approval of the study protocol must also be provided in the manuscript.

Field-specific reporting

Please select the one below that is the best fit for your research. If you are not sure, read the appropriate sections before making your selection.

☒ Life sciences ☐ Behavioural & social sciences ☐ Ecological, evolutionary & environmental sciences

For a reference copy of the document with all sections, see nature.com/documents/nr-reporting-summary-flat.pdf

Life sciences study design

All studies must disclose on these points even when the disclosure is negative.

Sample size	No statistical methods were used to predetermine sample sizes. The sample size was chosen based on previous experience in the laboratory and the literature. For multiomics one biological samples was analyzed. All n values are clearly stated in Figure legends.
Data exclusions	No data were excluded from any experiment if the cells expressed both mCherry and CFP reporter proteins
Replication	All experiments, but the multiomics analysis, were repeated at least 3 times with independent samples. Results were always very similar.
Randomization	No experiment was randomized
Blinding	The investigators were not blinded. Blinding was not performed because values derived from all experiments were quantitative and did not require subjective interpretation.

Reporting for specific materials, systems and methods

We require information from authors about some types of materials, experimental systems and methods used in many studies. Here, indicate whether each material, system or method listed is relevant to your study. If you are not sure if a list item applies to your research, read the appropriate section before selecting a response.

Materials & experimental systems

n/a	Involved in the study
☐	☒ Antibodies
☐	☒ Eukaryotic cell lines
☒	☐ Palaeontology and archaeology
☒	☐ Animals and other organisms
☒	☐ Clinical data
☒	☐ Dual use research of concern
☒	☐ Plants

Methods

n/a	Involved in the study
☒	☐ ChIP-seq
☐	☒ Flow cytometry
☒	☐ MRI-based neuroimaging

Antibodies

Antibodies used

See antibodies supplementary table in Supplementary Information

Validation

-anti-ARX: https://www.rndsystems.com/products/human-arx-antibody_af7068?gclsrc=aw.ds&gad_source=1&gad_campaignid=20161432922&gclid=CjwKCAjwu9fHBhAWEiwAzGRC__ngDboCXM8vVd395FH8E0E3iNi8BRwHWrgEUyVAGg1icfhO9apA-BoCgV8QAvD_BwE
-anti-Cleaved Caspase-3 (Asp175): https://www.cellsignal.com/products/primary-antibodies/cleaved-caspase-3-asp175-antibody/9661?srsltid=AfmBOoqNNmS9EfOs5dsQIM5rm8tMt2bQkYXy6IZ4tkJkWHm3mBKidCXa
-anti-FoxA2/HNF3β (D56D6): https://www.cellsignal.com/products/primary-antibodies/foxa2-hnf3b-d56d6-xp-rabbit-mab/8186?srsltid=AfmBOorSrTVQsPC9akAuSEQRNAumSNzd4n9dTd2Jcy-z18qzS9-mAPs-
-anti-GFP: https://www.antibodiesinc.com/products/anti-green-fluorescent-protein-antibody-gfp
-anti-glucagon: https://www.sigmaaldrich.com/DE/en/product/sigma/g2654?srsltid=AfmBOorE2g_T_1WFOXLlj5ry4B61IxnF7Rz3GZxLaYF2VxiZVbzZS68R
-anti-glucagon (FITC): https://www.novusbio.com/products/glucagon-antibody-09_nbp2-21803?srsltid=AfmBOorKxTRoLsPspM3iOQ2z7bC_hNQZTNiC7r5vJNM_TybrZVsPF2JE
-anti-insulin: https://www.bio-rad-antibodies.com/static/datasheets/5330-/pig-porcine-insulin-antibody-5330-0104g.pdf
-anti-insulin (APC): https://www.bdbiosciences.com/en-de/products/reagents/flow-cytometry-reagents/research-reagents/single-color-antibodies-ruo/alexa-fluor-647-mouse-anti-insulin.565689?tab=product_details
-anti-Ki67: https://www.abcam.com/en-us/products/primary-antibodies/ki67-antibody-ab15580?srsltid=AfmBOopkBQlUbp0XLeWE1tZHHNXCj2y1aFlt98XR1KqbuzLuF5EzAy2k
-anti-RFP (5F8): https://www.ptglab.com/products/RFP-antibody-5F8.htm?srsltid=AfmBOorw9qzEqTVsu4WvSl93K5ftQ0YkMJA90XFZN4wGzFgVwEmkgy4P
-anti-NEUROG3: https://www.avivasysbio.com/neurog3-antibody-oacd05949.html?srsltid=AfmBOorZLspFIy_Kadas2ekIeD9MMm_AYQQ9WlDjBIPHL_HgSD0l3uMN
-anti-nestin: https://www.abcam.com/en-us/products/primary-antibodies/nestin-antibody-10c2-neural-stem-cell-marker-ab22035?srsltid=AfmBOorlbWsvI7iaAh7_YCEX-0J5VP_Fd4560XVSJzTj3ERfBs59gEPT
-anti-NKX2-2: https://www.abcam.com/en-us/products/primary-antibodies/nkx22-antibody-nx2-294-ab187375?srsltid=AfmBOorUMni53kR77s5QIwhUUHYGcuwTqTNml3MOWLWpa03m825rcM48
-anti-NKX6-1: https://www.novusbio.com/products/nkx61-antibody_nbp1-82553?srsltid=AfmBOooIUhELnP-_0Mgys3qlS81SphHTt5HTVK2K8l0W_wKQ77Bnata4
-anti-Oct3/4: https://www.scbt.com/p/oct-3-4-antibody-n-19?srsltid=AfmBOoqpPBUg45b9tCdpTcYakP5OkRglkqXU5ZjRIFAC6AY5Jyji5OvD
-anti-PDX1: https://www.rndsystems.com/products/human-pdx-1-ipf1-antibody_af2419?gclsrc=aw.ds&gad_source=1&gad_campaignid=20161432922&gclid=CjwKCAjwu9fHBhAWEiwAzGRC_w_aeLMqSaUUhlDY91SBG-cGM7hBKlmIRmYOvSh2fZZWtMPlwKkyUhoCxEoQAvD_BwE
-anti-PAX4: https://www.thermofisher.com/antibody/product/PAX4-Antibody-Polyclonal/PA1-108
-anti-SOX17: https://www.neuromics.com/ittrium/reference/D8x1ba7x8x1/Datasheet.pdf?srsltid=AfmBOopz3w95dn7ygse-PeXitv7UgafByCo_2vAQvQ-6I84g62nowfVy
-anti-SLC18A1: https://www.atlasantibodies.com/products/primary-antibodies/triple-a-polyclonals/anti-slc18a1-antibody-hpa063797/
-anti-SNAIL: https://www.rndsystems.com/products/human-snail-antibody_af3639
-anti-beta III tubulin: https://www.abcam.com/en-us/products/primary-antibodies/beta-iii-tubulin-antibody-neuronal-marker-ab18207?srsltid=AfmBOopE72MpHCGS3kmjpmk3Mdd3dQ44l5LyL_qExB3Mg3REj6uWsglo

Eukaryotic cell lines

Policy information about cell lines and Sex and Gender in Research

Cell line source(s)

HMGUi001-A-46 (sex: female; Lickert lab)
HMGUi001-A-4 (sex: female; Lickert lab)

Authentication

HMGUi001-A-4 RRID: CVCL_ZZ82

Mycoplasma contamination

All cell lines were tested routinely for mycoplasma and resulted negative.

Commonly misidentified lines
(See ICLAC register)

No commonly misidentified cell lines were used in this study

Plants

Seed stocks	N/A
Novel plant genotypes	N/A
Authentication	N/A

Flow Cytometry

Plots

Confirm that:

☒ The axis labels state the marker and fluorochrome used (e.g. CD4-FITC).

☒ The axis scales are clearly visible. Include numbers along axes only for bottom left plot of group (a 'group' is an analysis of identical markers).

☒ All plots are contour plots with outliers or pseudocolor plots.

☒ A numerical value for number of cells or percentage (with statistics) is provided.

Methodology

Sample preparation	Single cell suspensions of undifferentiated or differentiated cells were fixed for 20 min in 4%PFA, permeabilized/blocked and stained with the mentioned primary and secondary antibodies or conjugated antibodies and their isotype controls.
Instrument	BD Facs Aria III
Software	FlowJo™ v10.7.1
Cell population abundance	mCherry+ and/ or CFP+ cells were sorted by flow cytometry (see Fig.1 and Suppl. Fig. 2)
Gating strategy	For FSC/SSC plot the gates were placed surrounding the main population. For FSC-A/ FSC-W plot, the gates were placed to include only single cell population. Gate for positive/negative populations were put based on the unstained, isotype or secondary antibody control.

☒ Tick this box to confirm that a figure exemplifying the gating strategy is provided in the Supplementary Information.
