# Supplementary material for: Resolving human α versus β cell fate allocation for the generation of stem cell-derived islets
Source: Nat Commun. 2026 Jul 9;17:6050. doi: 10.1038/s41467-026-75255-7 (PMC13351057; doi:10.1038/s41467-026-75255-7)
Supplement: Supplementary file 3 — Transparent Peer Review file [file 41467_2026_75255_MOESM3_ESM.pdf]

nature portfolio

# Peer Review File

# Resolving human α versus β cell fate allocation for the generation of stem cell-derived islets

Corresponding Author: Professor Heiko Lickert

Parts of this Peer Review File have been redacted as indicated to remove third-party material.

**This file contains all reviewer reports in order by version, followed by all author rebuttals in order by version.**

Version 0:

Reviewer comments:

Reviewer #1

(Remarks to the Author)
Previous genetic studies in mice have proposed a model for alpha ane beta cell specification in which ARX and PAX4 undergo cross-inhibitory regulation. The authors have now generated a hPSC line with a double KI reporter to track ARX and PAX4 during islet differentiation. They Ccmbining the system with single-cell analysis, and data from previously published single-cell datasets, they propose a model in which PAX4 indirectly induces ARX expression in endocrine progenitors, which inhibits PAX4 expression and thereby induces alpha cell specification. Based on multiomic analysis of cells at this stage, they generate a GRN to identify putative targets of these genes, including FOXO1. They also use this tool to test two candidate chemical inducers of beta cell differentiation.

The main interest of this manuscript is that it offers a human reporter tool to simultaneously follow ARX and PAX4 activity, which can be very useful if it is made available for the community, as it could facilitate manipulation of beta vs alpha cell formation in vitro, in particular for large-scale screening efforts.

The proposed mechanism for alpha vs beta cell specification is different from what was known from mice genetic studies. However, it is not entirely supported by their data. The effects of compounds have some interest, although further experiments are needed to define the targets involved. The multi-omic analyses from different time points has been used to build a GRN (and did not require the dual reporter) but it has not yielded novel targets. Multi-omics has also been used to quantify the abundance of cell types in the samples, and to check for some markers.

Concerning artemether, it did not clearly arise from the GRN studies, at least from the explanations, and it is unclear why the dual reporter line is relevant given that the major analysis is with multi-omics. The analysis of artemether is of interest, given the controversy in the field, but it does not really provide more clarity to the field.

In summary, this is a potentially interesting tool, which would need to be made widely available. The authors could have taken advantage of this tool for more in-depth studies and should be encouraged to do so.

Other specific points:
In line 274, the authors state that “we queried our single cell ATAC-Seq data for potential PAX4 and ARX binding sites within each other’s putative cis-regulatory elements (CRE). While we found motifs of each TF in putative CREs of the other gene, there was no correlation between the accessibility of the CRE and TF expression”. Data regarding these correlations should be presented with the manuscript. Moreover, co-accessibility and motif detection is an indirect method to assess TF binding. One good way to discard binding of each TF in each other’s CRE for good would be to use ChIP-seq experiments for ARX and PAX4 binding. If this is one of the conclusions of the manuscript this is an essential experiment.

The authors present FOXO1 as one of the putative targets identified from the GRN. The role of FOXO1 in alpha cells and endocrine cell de/differentiation has been proposed previously. As the authors point out, FOXO1 knockdown or inhibition results in dedifferentiation and loss in cell identity, while an inhibitor of FOXO1 was reported to cause increased hESC differentiation towards beta cells, or else dedifferentiation. The authors do not explain well their own findings in relation to these previous studies, one does not get the impression that these new findings bring more clarity to the field. It would be interesting to continue with the differentiation experiment to see how this impacts the beta to alpha cell ratio upon FOXO1 inhibitor treatment, rather than focusing just on the assessment of the percentage of PAX4-mCherry+ or ARX-CFP+ cells.

Given that the authors propose that the multimome GRN is useful to identify druggable hubs for optimization of differentiation protocols, it would be of interest to identify and validate putative unknown targets of either PAX4 and ARX.

Regarding PAX4 modulation upon ARX: in line 300, the authors state that “Taken together these results suggest a model of human β cells differentiation in which PAX4 and ARX do not stochastically cross inhibit each other, but instead PAX4 in EPs indirectly induces ARX expression, that in turn indirectly represses PAX4 expression, inducing differentiation toward the α cell lineage” They should re-state this sentence in some way to propose this mechanism as a hypothesis of what they are seeing.

The authors propose that PAX4 activates ARX expression. In mouse genetic studies, loss of PAX4 does not lead to depletion in alpha cell formation, rather the opposite (line 53: KO mice for Pax4 have increased number of α cells and virtually no β cells). Also, from previous assays in hESC models (Gage et al, 2014), overexpression of PAX4 leads to decreased ARX expression. In another study by Lau et al (Nat Commun, 2023), a stable knock-down on PAX4 in EndoC-βH1 cells led to a decrease in ARX expression, whereas its KO in a stem cell system that was differentiated to islets shown increased expression of genes contributing to an alpha cell signature, including ARX. In general, the authors should check other previously published papers using stem cell systems to study these two transcription factors (e.g. Lau et al., Nat Commun, 2023; Gage et al, Islets, 2014; Gage et al., PLoS One, 2015), to examine how plausible this model might be, and to enrich the manuscript.

In addition, the authors could test in genetic model systems whether PAX4 affects ARX gene activation, and compare their results to the ones from previous studies.

Minor issues
In the drawing the T2A and endogenous PAX4 coding exons/sequences would be useful to see how the final PAX4 protein is generated.

· Line 151: “The percentage of fluorophore-positive cells started to increase in the transition between late PPs and EPs (day 3 of S4 – S4d3)”. The Figure seems to shown it starts to increase earlier.

· Figure 4A: there is no point in showing a color to label PAX4 binding sites if there is no binding site for this TF in the figure.

· Figure 4B: I do not fully understand how they calculated the direction of the regulation.

Reviewer #2

(Remarks to the Author)
Melis Akgün Canan and colleagues present a compelling study exploring how two well-known transcription factors, ARX and PAX4, influence the fate decisions of alpha and beta cells using iPSC reporter lines. The authors utilize a range of advanced technologies, including transcriptome studies and stem cells, alongside conventional methods like flow cytometry and immunofluorescence staining. Their findings provide a comprehensive overview of the mutual impact of PAX and ARX on alpha and beta differentiation. Additionally, the study demonstrates that FOXO1 inhibition and the controversial compound Artemisinins promote iPSC-derived beta cell differentiation. Overall, the study is well-designed and executed, and the discussion is clear and well-developed. However, several comments need to be addressed.

Major Comments:
The authors use PAX4 and ARX reporter cell lines to sort cells and then run sc-RNAseq and snATACseq separately, rather than a combined multiome ATAC+Gene expression analysis. They then integrate these datasets and examine PAX4 and ARX gene patterns within the sorted data. This approach gives the impression of a closed loop in developing the topic. To reach unbiased conclusions, computational models such as RNA velocity and trajectory analysis should be conducted in parallel to validate the findings.

Minor Comments:
1. Lines 112-116: We notice differences in the UMAP shape and labeling in this manuscript compared to the original manuscript, ref 37. The authors should clearly explain how they recapitulated the reference data, including whether they obtained raw FASTQs, how they processed the data, and how they performed annotation and clustering.
2. Lines 199-200: Which algorithm was used for integrating the two datasets? Was it weighted nearest neighbor or another method? It appears to be non-multiomic, donor-matched separate scRNA/snATAC sequencing. If both sequencing types influenced the clustering, how were weights assigned?
3. Supplementary Figure 3C: It is unclear how alpha vs. alpha progenitors and beta vs. beta progenitors were divided. The dots patterns were quite similar within each subcluster.
4. Lines 279-280: How did you determine the putative ARX binding site in its own gene and promote transcription by simply examining peak coverage? Please elaborate on the method or reasoning.
5. Methods 7.4: Which tool was used to visualize the calculated GRN after motif identification?
6. Lines 208-282 and Figure 4B: How was this chart created? Please provide details in the Methods section.
7. Lines 287-299 and Fig. 4B to E: In Fig4b, FOXO1didn’t have direct or indirect interaction with PAX4. How does FOXO1

inhibition support the working model in Fig 4E? This point requires clarification.
8. Figure 5A/B/C: Were these figures generated from reporter-based sorted cells? Did this multiome data project on the data in Figure 2? The cell population percentages in the control were quite similar to the unsorted data in Figure 2C. What are the PAX4 and ARX expression levels in Figure 5B? Do these two show the similar patterns like in Fig2B?
9. Figure 5G: The gating is different between DMSO and Artemether.
10. Methods 7.3: Specify the hyperparameters used for each algorithm, such as mitochondrial gene fraction cut-off, minimum number of genes, TSS enrichment, or nucleosome signals, et al. Additionally, it seems multiple doublet detection algorithms were used and it seems excessive compared to other studies. Could you explain the rationale behind this choice?
11. Methods 7.3 (GRN Inference): Validation of inferred networks using independent datasets is crucial for building a reliable GRN. The authors should provide validation using external datasets.
12. Line 601: Specify the nature of the internal reference dataset. What type of data does it include, how many cells, and was it previously published? Since annotation and labeling are critical aspects of scRNA analysis, it would be beneficial to include UMAP and gene expression visuals of your reference in the supplemental data.

Reviewer #3

(Remarks to the Author)
Key results

Pluripotent Stem cell-derived pancreatic islets do not perfectly mimic human or mouse islets in vivo, partially due to the reduced differentiation efficiency from the endocrine progenitor stage to the beta-cell stage. To improve our understanding of the mechanisms of endocrine cell specification during human pancreas development, Canan et al. investigate whether the classic model that describes ARX and PAX4 stochastic expression patterns in mice is conserved in human pancreas development. They generate a double reporter human induced pluripotent stem cell (iPSC) line for both ARX and PAX4 (ARXCFP/CFP; PAX4mCherry/mCherry). Using different experimental strategies, including single-cell multi-omic analysis, the authors conclude that ARX and PAX4 expression does not overlap in progenitor cells, but that PAX4 is co-expressed with NGN3+ endocrine progenitor and expressed in pre-beta and beta cells. In NGN3+/PAX4+ progenitors that develop into pre-alpha cells, PAX4 is suggested to (indirectly) activate ARX expression. From multi-omic datasets, the authors predict a gene-regulatory network (GRN) in which ARX and PAX4 do not directly interact with each other and identify FOXO1 as being regulated by ARX. Finally, the authors test the effect of artemether on alpha vs beta specification in their iPSC double tracer model and find some effects on proliferation and segregation into different endocrine fates. To summarise, the authors employed a novel iPSC model with dual reporters that allowed them to study the expression and dynamics of ARX and Pax4 and led them to propose a new working model for ARX and PAX4 expression patterns and function in human pancreas development.

Validity

-Overall, the authors employed appropriate techniques to address their research questions, the data seem valid, and the authors' interpretation of their work is appropriate.

-One of the main claims of the manuscript is that PAX4 and ARX expression do not overlap in cells. However, it is possible (but difficult to tell from the graphs alone) that Figures 1B, 2E and 3C show double positive (PAX4+ / ARX+) populations. Additionally, the authors find cells that are double positive for the fluorescent tags CFP (for ARX) and mCherry (for PAX4), but they argue that mCherry is still detected for a while after PAX4 expression. Although possible, it is difficult to prove that these ‘fluorescent’ double-positives are not ‘true’ double positives.

-The proposed working model of ARX and PAX4 mechanism (Figure 4E) states that all ARX+ cells originate from PAX4+ cells. However, the paper shows the presence of a population of cells that are CFP-ARX+/ mCherry-PAX4- which might have never expressed PAX4. Since such cells might originate from a separate progenitor population compared to the CFP-ARX+/mCherry-PAX4+ cells, the authors should leave this option open in their model/discussion. The authors conclude (ca line 300) that in endocrine progenitors, PAX4 indirectly represses ARX and that, in turn, ARX indirectly represses PAX4. This cannot be concluded based on the shown data.
Significance

-The manuscript is a valuable contribution to the scientific discussion on human pancreas development and sheds new light on the differences between human vs murine endocrinogenesis.

-The authors introduce an interesting iPSC line that will allow for further research into the cellular mechanisms and lineage fate decisions.

Data and methodology

Overall, the data are of high quality and are discussed clearly in the text. However, the kinetics experiment in supplementary figure 2 raises some concerns. In this figure, the author compares protein expression dynamics of mCherry and CFP fluorophores to endogenous PAX4 and ARX expression and concludes that mCherry is expressed for longer than PAX4,

while CFP more faithfully represents ARX expression. To prove this, the author quantifies immunofluorescent stainings for ARX and PAX4 in an unedited, wild-type hiPSC line, and compares it to the quantification of CFP and mCherry from the other, CRISPR-engineered line. Since differentiation experiments can have some level of variability, and CRISPR engineering can partially impact cell identity and differentiation capacity, the author is advised to quantify the expression of PAX4 and ARX directly on the engineered hiPSC line.

Quantification of the immunofluorescence stainings throughout the manuscript will strengthen the quality of the data.

Analytical approach

The statistical analyses are conducted thoroughly and accurately.

Suggested improvements

-Figure 2E: please show the percentage of cells with NGN3, ARX and/or PAX4 expression. Even though the eagle-eyed view shows distinct populations, it is hard to tell whether there are smaller double/triple positive populations.

-Supplementary figure 2: The authors measured endogenous protein levels of ARX and PAX4 to map their expression patterns. This expression pattern is key for their interpretation of the measurements of the fluorescent tags and consequently for their proposed mechanism. However, the authors do not show the endogenous proteins in the mutant line even though the gene and protein patterns of ARX and PAX4 could differ between the wild-type line and the mutant line. Additionally, figure S2C is strangely plotted, and the time points are unclear. Finally, the replicate number is too low (n=2 or more). Please add additional information and data to make this figure more robust.

-What are the levels of ARX (protein and mRNA), CFP (protein and mRNA), PAX4 (protein and mRNA) and mCherry (protein and mRNA) in the double reporter iPSC line in the different stages of differentiation (from S3 to end of S6). How does the expression of ARX and PAX4 (protein and mRNA) in the double reporter iPSC line compare with the expression of those in the wild-type line?

-Figure 3C: as previously mentioned, it is difficult to tell whether ARX+/PAX4+ cells are present. Please add a quantification of the immunofluorescent staining and consider flow cytometry for ARX and PAX4 expression analysis after reaggregation. This could provide valuable additional information.

-Figure 4A: The figure is difficult to read (e.g. PAX4 on the PAX4 locus on the left corner of the graph). Further clarification in the graph such as highlighting the PAX4 locus or more details in the text could improve the reader’s comprehension.

-Figure 6: Since Artemether was shown to increase the proportion of INS+ beta cells and reduce GCG+ alpha cells at the end of the differentiation protocol, it is recommended to add the flow cytometry analysis of dCaspase3 and Ki67 at the end of Stage 6 too.

To make a stronger and conclusive claim on the newly described PAX4/ARX model, a more refined experimental design that allows performing lineage tracing on the endocrine progenitors would be required (for example – lentiviral cell tagging and multiomics at multiple time points). In this manuscript, there is no way to tell whether all ARX+ cells had previously expressed PAX4. Please add a comment about the current limitations of the mCherry-PAX4/ CFP-ARX line employed in this study in the discussion.

Clarity and context

-The manuscript is very well written, and the data is very well structured.
-Figure 4E (working model) could be more detailed with more space for established and putative mechanisms.

References

-The authors referenced key past works and placed their data in context.

Version 1:

Reviewer comments:

Reviewer #1

(Remarks to the Author)

The authors have improved the manuscript, which now provides convincing evidence that ARX and PAX4 are only sometimes co-expressed during human pancreatic endocrinogenesis, both in fetal pancreas and hiPSC-differentiated cells. Their extensive multiomics analyses suggests that ARX and PAX4 regulation is indirect. In addition, in vitro potency assays using purified cell populations show that PAX4+ give rise to beta cells, and ARX+ or ARX+PAX4+ fluorescent cells preferentially gave rise to alpha cells. They also removed the FOXO1 inhibitor data, and include ReST inhibitor studies which strengthen the study. The GRN analysis also provides many possible upstream regulators of endocrine differentiation genes.

A central proposal of the study is that ARX-PAX4 regulation is not direct. This may be correct, but the evidence is still largely suggestive . The chromatin IP experiments would be needed to prove this point. The authors mention the limitations posed by antibody availability, but the discussion of negative ChIP results in other organs or cell types is not, in itself, compelling, as there could be direct repressor effects during transient stages of endocrinogenesis—an effect that would not necessarily be captured in gut organoids or beta cell lines. In the absence of direct evidence, it seems premature to claim that the data establish a fundamentally new differentiation model. That said, the single-cell analyses appear robust and provide a strong foundation for future experiments aimed at identifying the direct transcriptional regulators of ARX and PAX4.

The observation that PAX4 knockout leads to elevated ARX expression raises a serious challenge to the authors' hypothesis that PAX4 induces ARX. The authors speculate that this apparent contradiction could reflect compensatory activity of other upstream regulators that promote ARX expression despite loss of PAX4. However, this explanation remains highly speculative in the absence of supporting data. If the knockout results suggest the opposite of the proposed model, additional evidence is needed to justify why the authors' interpretation is nevertheless likely to be correct.

Regarding the artemether experiments, treatment leads to a modest increase in $INS^{+}$ cells following sorting of $ARX\text{-}CFP^{+}$ or $PAX4\text{-}mCherry^{+}/ARX\text{-}CFP^{+}$ populations. The authors conclude that these data rule out α-to-β transdifferentiation because glucagon-positive cell numbers remain unchanged, which could also be due to compensatory changes. The attribution of the small increase in insulin-positive cells to residual endocrine progenitors due to incomplete sorting is plausible but not definitive. Similarly, while the use of chemical GABA agonists and antagonists suggests that GABA signaling is unlikely to mediate the observed effects, additional approaches would be required to conclusively exclude this mechanism. Importantly, no alternative mechanism is proposed to explain the changes in $INS^{+}$ cells. The authors appropriately acknowledge that the effects are modest, do not impact insulin secretion, and that further optimization of artemether dosage and treatment duration would be necessary.

There is another recent dual alpha vs beta reporter systems (e.g. Sci Rep 2024 Aug 27;14:19863) which would make sense to mention

Reviewer #2

(Remarks to the Author)
The revised manuscript is substantially improved, and the authors have addressed the majority of my concerns from the first review. The narrative is clearer, the GRN analysis is better contextualized, and the PAX4–ARX model is now presented with more appropriate caution. I appreciate the additional methods clarifications, improvements in figure legends, and the strengthened logic linking multiomics to the proposed regulatory framework.
At this stage, several clarifications are needed:
1. Please clarify whether mCherry protein persists after endogenous PAX4 downregulation (important for interpreting double-positive reporter cells in Fig. 2 and Fig. 3E).
2. Clarify the exact experimental design underlying Suppl. Fig. 2B (bulk vs sorted populations, which transcripts are measured, normalization method).
3. In lines ~316–318, the manuscript states that "REST seems to repress PAX4… suggesting REST as a direct upstream regulator of PAX4 and endocrine induction." However, the GRN table does not indicate a strong or consistent REST→PAX4 repressive relationship, nor a REST→ARX interaction. REST instead clusters with a non-endocrine/progenitor module (HES1, ID2, HMGA1/2, SOX9), with negative associations to INSM1 and PAX6.
4. In Suppl. Fig. 7C, both INS and GCG expression appear visually reduced across conditions. The description around lines ~366–367 does not explain this pattern, and readers may find it confusing.

Reviewer #3

(Remarks to the Author)
The authors have satisfactorily addressed all the comments raised.

Version 2:

Reviewer comments:

Reviewer #2

(Remarks to the Author)

The authors have addressed my concerns. I have no further comments.

**Open Access** This Peer Review File is licensed under a Creative Commons Attribution 4.0 International License, which permits use, sharing, adaptation, distribution and reproduction in any medium or format, as long as you give appropriate credit to the original author(s) and the source, provide a link to the Creative Commons license, and indicate if changes were made.
In cases where reviewers are anonymous, credit should be given to 'Anonymous Referee' and the source.
The images or other third party material in this Peer Review File are included in the article's Creative Commons license, unless indicated otherwise in a credit line to the material. If material is not included in the article's Creative Commons license and your intended use is not permitted by statutory regulation or exceeds the permitted use, you will need to obtain permission directly from the copyright holder.
To view a copy of this license, visit https://creativecommons.org/licenses/by/4.0/

**Point-by-point response to the reviewer´s comments.**

We thank the reviewer for taking the time to evaluate our work and for their insightful comments. Addressing them has greatly improved our study. We have revised the text to address all the comments raised (changes are highlighted in yellow in the revised manuscript). Below is a point-by-point response with our responses shown in blue.

We included one new main figure and five additional supplementary figures.

In particular, we:

- Performed extensive temporal analysis on the expression of both *PAX4* and *ARX* during *in vitro* differentiation toward β cells in both the ARX/PAX4 cell line and wildtype parental line, both at mRNA and protein level (Suppl. Fig. 2; Fig. 2G)
- Carried out extensive analysis on the number of $ARX^+/PAX4^+$ cells and TFs expression within this population during both *in vivo* and *in vitro* human differentiation toward β cells (Fig. 1B; Suppl. Fig. 1A; Suppl Fig. 3I-L)
- Conducted extensive analysis on the number of $ARX^+/PAX4^+$ cells after potency assays (Fig. 3; Suppl. Fig. 5)
- Performed new potency assays for mid endocrine progenitors after sorting at S5d4 (Fig. 3)
- Extensively revised the methods used for the generation of our gene-regulatory network (GRN) (Fig. 4)
- Added a new drug perturbation assay, inhibiting an identified GRN target, REST (Fig. 4; Suppl. Fig. 6)
- Performed an extensive characterization of artemether-treated SC-islets downstream diverse treatment schemes (cellular composition, cell proliferation and death, and insulin secretion – Fig. 6; Suppl. Fig. 8)
- Tested the ability of artemether to trigger α-to-β transdifferentiation *in vitro* (Suppl. Fig. 8)
- Investigated GABA signaling activation as a possible mechanism of action of artemether (Suppl. Fig. 9)

Finally, we increased sample sizes where requested, quantified immunofluorescence through the entire manuscript, modified the text to improve clarity of the Results and Methods sections, and put the present study in context with previous literature in the Discussion section.

We hope the reviewers and editor will find the revised manuscript now suitable for publication in *Nature Communications*.

**REVIEWER COMMENTS**

Reviewer #1 (Remarks to the Author):

Previous genetic studies in mice have proposed a model for alpha ane beta cell specification in which ARX and PAX4 undergo cross-inhibitory regulation. The authors have now generated a hPSC line with a double KI reporter to track ARX and PAX4 during islet differentiation. They Ccmbining the system with single-cell analysis, and data from previously published single-cell datasets, they propose a model in which PAX4 indirectly induces ARX expression in endocrine progenitors, which inhibits PAX4 expression and thereby induces alpha cell specification. Based on multiomic analysis of cells at this stage, they generate a GRN to identify putative targets of these genes, including FOXO1. They also use this tool to test two candidate chemical inducers of beta cell differentiation.

The main interest of this manuscript is that it offers a human reporter tool to simultaneously follow ARX and PAX4 activity, which can be very useful if it is made available for the community, as it could facilitate manipulation of beta vs alpha cell formation in vitro, in particular for large-scale screening efforts.

The proposed mechanism for alpha vs beta cell specification is different from what was known from mice genetic studies. However, it is not entirely supported by their data. The effects of compounds have some interest, although further experiments are needed to define the targets involved. The multi-omic analyses from different time points has been used to build a GRN (and did not require the dual reporter) but it has not yielded novel targets. Multi-omics has also been used to quantify the abundance of cell types in the samples, and to check for some markers.

Concerning artemether, it did not clearly arise from the GRN studies, at least from the explanations, and it is unclear why the dual reporter line is relevant given that the major analysis is with multi-omics. The analysis of artemether is of interest, given the controversy in the field, but it does not really provide more clarity to the field.

In summary, this is a potentially interesting tool, which would need to be made widely available. The authors could have taken advantage of this tool for more in-depth studies and should be encouraged to do so.

We would like to thank the reviewer for the constructive suggestions that helped us to improve our manuscript. We appreciate all comments and answer below to each one of the points raised.

As highlighted by the reviewer, the ARX/PAX4 double reporter iPSC line will be extremely useful for the stem cell community and will be made available upon request.

While we could not fully prove our proposed mechanisms for α versus β cell specification, the proposed mechanism stems from multiple lines of bioinformatic and biological evidence. We analyze the expression pattern of the two TFs during human gestation (Fig. 1B; Suppl. Fig. 1A) and SC-islets differentiation *in vitro*, derived from two different differentiation protocols in two different laboratories (Suppl. Fig.1B-C; Suppl Fig. 3I-L) using sc/snRNA-seq. In all datasets, we found very little co-expression of *ARX* and *PAX4* at the mRNA level. Using multiomics data from our reporter cell line, we then generated a GRN, where no direct interaction was found between ARX and PAX4 (Fig. 4A-B), suggesting that the two TFs do not directly regulate each other´s expression. At the protein level, staining of wild type clusters undergoing direct β-cell differentiation against both endogenous TFs (Suppl. Fig. 2D) detected single positive cells for ARX

and PAX4 TF protein in the same aggregate at all analyzed stages (S4, S5 and S6), but detected maximum 1% of double positive cells ($ARX^+$/$PAX4^+$) at S4d3 and less than 1% of these cells from S4d5 onward. If PAX4 and ARX TFs were co-expressed in the same endocrine progenitor to oppose each other's function, we would expect a much higher percentage of pancreatic progenitors (PP at S4) or endocrine progenitors (EP at S5 and S6) co-expressing the two TFs. Taken together, this data further supports our model in which ARX and PAX4 act to ensure α- and β-cell differentiation, respectively, but do not directly interact with each other in a common EP by antagonizing each other's function. Supporting this notion, PAX4 acts mainly as an activator at the EP stage in our system and GRN, while ARX exhibits mainly a transcriptional repressor activity. In addition, potency assays after sorting of reporters positive populations at both S5d4 and S5d7 showed that any cell expressing *ARX* will eventually differentiate into an α-cell (Fig. 3C and Suppl. Fig. 5C).

We better explained the generation and analysis of the multiomics data in both the Results and the Material and Methods section of the manuscript. Moreover, we now construct the GRN with the widely used tool Pando to make it more reliable. We have changed the text to make it more easily understandable and highlighted novel players in the GRN connecting PAX4 and ARX, as well as already described ones in the Discussion section.

Regarding the treatment with artemether, the reporter line is relevant because the multiomics analysis in Fig. 2 allows the biological validation downstream of any multiomics conclusions with or without treatments, and it is based on reporter positive sorted populations. This sorting step allowed enrichment and annotation of $ARX^+$, $PAX4^+$ and $ARX^+$/$PAX4^+$ cells, together with the study of cell populations that would have been lost or underrepresented in a sc-RNA-seq experiment without fluorescent reporter enrichment. We also added experiments using different treatment schemes to understand if 1) the artemether-induced EP would give rise to more and/or better β-like cells (Suppl Fig. 8A-E), and 2) artemether treatment of α-like cells would results in α-to-β transdifferentiation *in vitro* (Suppl Fig. 8F-G).
In addition, we added experiments to test its possible mechanisms of action based on previously published data (Suppl. Fig. 9). Artemether has been suggested to activate the GABA signaling cascade (Li *et al.*, 2017). Treatment of our cell line with GABA at S4 and S5 *per se* did not trigger any difference in the number of $ARX^+$ and $PAX4^+$ cells at S5d7 (Suppl. Fig. 9B). Interestingly both treatments with Picrotoxin and Baclofen (a $GABA_A$ antagonist and a $GABA_B$ agonist, respectively) resulted in increased numbers of $ARX^+$/$PAX4^+$ and $ARX^+$ cells (Suppl. Fig. 8C), having both the opposite effect than artemether treatment. Taken together, this data excludes GABA signaling as a potential mechanism of action of artemether for induction of endocrine progenitors *in vitro*. As already stated in the manuscript, artemether treatment induced upregulation of *NEUROG3* and *INSM1* expression specifically in EPs.

Other specific points:
In line 274, the authors state that "we queried our single cell ATAC-Seq data for potential PAX4 and ARX binding sites within each other's putative cis-regulatory elements (CRE). While we found motifs of each TF in putative CREs of the other gene, there was no correlation between the accessibility of the CRE and TF expression". Data regarding these correlations should be presented with the manuscript. Moreover, co-accessibility and motif detection is an indirect method to assess TF binding. One good way to discard binding of each TF in each other's CRE for

good would be to use ChIP-seq experiments for ARX and PAX4 binding. If this is one of the conclusions of the manuscript this is an essential experiment.

We agree that this is important information, data regarding ARX and PAX4 putative CREs and their correlation with TFs expression has been added to the Results part (Fig. 4A; Suppl. Fig. 6).

We agree with the reviewer, however due to the lack of antibodies amenable for ARX and PAX4 pulldown, the necessary ChIP-Seq (or similar) experiments are not possible. During this revision, we generated custom made antibodies against PAX4. Unfortunately, none of these newly generated antibodies performed better in western blot or immunofluorescence compared to the commercially available antibody that we already use. However, CHIP-qPCR performed on human small intestine organoids with overexpression of FLAG-tagged ARX and/or PAX4 showed no direct interaction between ARX and PAX4 in enteroendocrine cells (Lin et al., 2023, Science), a cell type developmentally closely related to pancreatic endocrine cells**.** Moreover, in a recent paper (Russ-Silsby and Lee, 2025), CUT and RUN assay on a stable β cell line (EndoC-βH1) transduced with FLAG-tagged PAX4 showed no binding of PAX4 on the ARX *locus*. Within the same locus, the authors also showed no overlap with differentially expressed genes in PAX4 KO stem cells-derived EP and PAX4 accessible peaks identified in the PAX4 overexpressing EndoC-βH1. This information has been added to the Discussion section.

The authors present FOXO1 as one of the putative targets identified from the GRN. The role of FOXO1 in alpha cells and endocrine cell de/differentiation has been proposed previously. As the authors point out, FOXO1 knockdown or inhibition results in dedifferentiation and loss in cell identity, while an inhibitor of FOXO1 was reported to cause increased hESC differentiation towards beta cells, or else dedifferentiation. The authors do not explain well their own findings in relation to these previous studies, one does not get the impression that these new findings bring more clarity to the field. It would be interesting to continue with the differentiation experiment to see how this impacts the beta to alpha cell ratio upon FOXO1 inhibitor treatment, rather than focusing just on the assessment of the percentage of PAX4-mCherry+ or ARX-CFP+ cells.

We thank the reviewer for the suggestion. We assessed the ratio of insulin- and glucagon-positive cells after FOXO1 inhibition at S5d7 and found no differences in the number of endocrine cells, showing that FOXO1 inhibition does not influence α or β differentiation during SC-islets differentiation *in vitro*. Consequently, we remove these experiments from the main figures and show them only here (please see below).

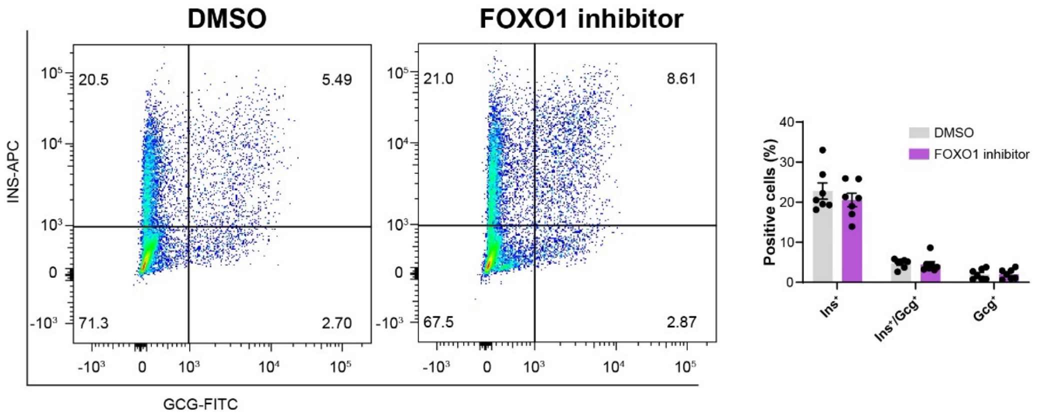

This result highlights the ARX/PAX4 cell line as an agnostic tool to test both endocrine induction and α versus β cell differentiation. In fact, differences in the number of reporters-positive cells at the end of S5 can indicate both altered endocrine induction (and EP numbers) and/or altered α or β cell differentiation. In this study we show both cases: continuous artemether treatment resulted into a dramatically increased number of EP at S5d7 that did not give rise to a proportionally increased number of endocrine cells at S6d14; on the other hand, REST inhibition resulted into a small increase in the number of EP that was mirrored by a small increase in the number of β cells at S6d14.

Given that the authors propose that the multimome GRN is useful to identify druggable hubs for optimization of differentiation protocols, it would be of interest to identify and validate putative unknown targets of either PAX4 and ARX.

We agree with the reviewer, and we tried two different approaches to comply.
By our GRN KLF5 seems to negatively regulate *PAX4* expression through regulatory elements in the endocrine progenitors. We tried to inhibit KLF5 using MLS264 (Bialkowska et al., 2013), but it unfortunately resulted into massive cell death.
Another interesting candidate that seems to interact with PAX4 is REST. By our GRN, REST seems to repress *PAX4* in endocrine progenitors, suggesting it as a direct upstream regulator of *PAX4* during human in vitro β cell differentiation. This last correlation is in line with literature where *REST* inhibition in pancreatic organoids, using the small molecule X5050, increases endocrinogenesis (Rovira et al., 2021). We inhibited REST, specifically at S5 and showed increases PAX4-mCherry$^+$ cells at S5d7 together with an increased number of INS$^+$ cells at the same stage (Fig. 4D-E). This data has been added to the Results section of the revised manuscript and discussed in the Discussion section. Other potential druggable targets (such as TAL2, ID4 and others) were included in the Discussion section.

Regarding PAX4 modulation upon ARX: in line 300, the authors state that "Taken together these results suggest a model of human β cells differentiation in which PAX4 and ARX do not stochastically cross inhibit each other, but instead PAX4 in EPs indirectly induces ARX expression, that in turn indirectly represses PAX4 expression, inducing differentiation toward the α cell lineage" They should re-state this sentence in some way to propose this mechanism as a hypothesis of what they are seeing.

We thank the reviewer for this suggestion. We restated this sentence as follows: "*Taken together, these results led us to hypothesize a model of human α versus β cell fate allocation in which PAX4 and ARX do not stochastically cross inhibit each other. Instead, we suggest that PAX4 in EPs would indirectly induce ARX expression with other upstream regulators. ARX in turn would directly reinforce its own expression and indirectly repress PAX4 expression, dominating differentiation toward the α cell lineage (Fig. 4F).*"

The authors propose that PAX4 activates ARX expression. In mouse genetic studies, loss of PAX4 does not lead to depletion in alpha cell formation, rather the opposite (line 53: KO mice for Pax4 have increased number of α cells and virtually no β cells). Also, from previous assays in hESC models (Gage et al, 2014), overexpression of PAX4 leads to decreased ARX expression. In another study by Lau et al (Nat Commun, 2023), a stable knock-down on PAX4 in EndoC-βH1 cells led to a decrease in ARX expression, whereas its KO in a stem cell system that was differentiated to islets

shown increased expression of genes contributing to an alpha cell signature, including ARX. In general, the authors should check other previously published papers using stem cell systems to study these two transcription factors (e.g. Lau et al., Nat Commun, 2023; Gage et al, Islets, 2014; Gage et al., PLoS One, 2015), to examine how plausible this model might be, and to enrich the manuscript.

We agree that this is important information, and we apologize that it was not properly explained in the first version of the manuscript. The Discussion section has been further expanded to include a more thorough comparison of ours and others´ data.
While we propose an indirect activation of *ARX* by PAX4, we also identify multiple upstream regulators of *ARX* expression (such as ONECUT1, MECOM, KLF5, POU2F2, GATA5, ID2, ID4, HMGA1, EGR1, IRX1, IRX2, RFX3, SOX2 and HHEX). Some of these transcription factors would specifically activate *ARX* while others are shared with *PAX4*. In this scenario, *Pax4* deletion in mouse would enhance the activity of these upstream regulators on *Arx* and reinforce differentiation towards α cells.
In contrast to our use of single-cell multiomics data, previous studies analyzed the effect of *PAX4* modulations using bulk-based technologies. In Gage *et al.*, 2014 the overexpression of *PAX4* in acute settings resulted in a reduction in glucagon-positive cells and *ARX* expression. Since the evaluation of *ARX* expression has been performed using bulk qPCR, and not at the single-cell level, the authors cannot discriminate between a reduction in *ARX* mRNA level per cell and a reduction of bulk *ARX* expression due to reduced $ARX^+$ cells number in their sample. Lau *et al.*, 2023 also performed bulk RNA-seq during SC islets differentiation at the end of PE, EP and SC-Islets stages - stages where PAX4 is not or no longer expressed whereas our dataset showed that it is at the mid-EP stage where lineage commitment occurs. Moreover, investigators in this study used 3 independent PAX4 KO hiPSC, but only see a “directionally consistent, albeit nonsignificant, derepression of alpha cell genes (ARX, GCG, TTR)”.

In addition, the authors could test in genetic model systems whether PAX4 affects ARX gene activation, and compare their results to the ones from previous studies.

We thank the reviewer for the suggestion. After multiple failed attempts to transfect EP cells at S5 in 2D or 3D with commonly used methods, we turned to the recently published viral like particles (VLPs – Geilenkeuser and Armbrust et al., 2025) for transduction of S5d4 cells. We differentiated wildtype iPSC in 2D until S5d4, we then transduced them with three different VLPs: empty, overexpressing *GFP*, or overexpressing *GFP-PAX4*. We isolated RNA after 24 hours and tested the mRNA levels of *GFP*, *PAX4* and *ARX*. As shown in the figure below, in line with our proposed model in which PAX4 does not directly induce *ARX*, overexpression of *PAX4* at S5d4 did not interfere with *ARX* expression at S5d5.  We add this preliminary data for the reviewer only.

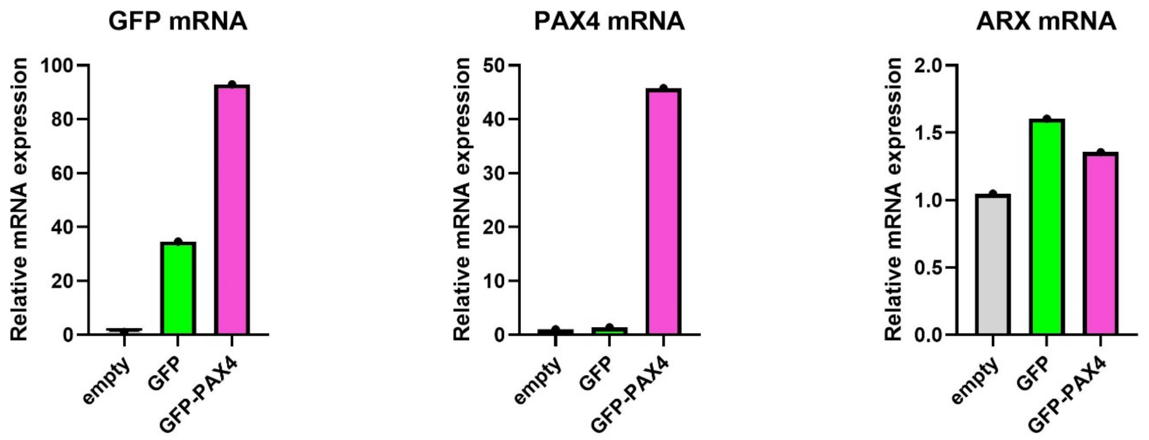


Minor issues
In the drawing the T2A and endogenous PAX4 coding exons/sequences would be useful to see how the final PAX4 protein is generated.

We thank the reviewer for the constructive suggestion, a schematic highlighting the structures of the final proteins has been added to Figure 1 (Fig. 1C).

· Line 151: "The percentage of fluorophore-positive cells started to increase in the transition between late PPs and EPs (day 3 of S4 – S4d3)". The Figure seems to shown it starts to increase earlier.

We thank the reviewer for raising this point. Yes, the number of $CFP^+$ cells started to increase at S4d2. The text has been changed accordingly.

· Figure 4A: there is no point in showing a color to label PAX4 binding sites if there is no binding site for this TF in the figure.

We thank the reviewer for the constructive suggestion; we updated Figure 4 with new data.

· Figure 4B: I do not fully understand how they calculated the direction of the regulation.

We agree that this is important information, and we apologize that it was not properly explained in the first version of the manuscript. We have now revised the generation of the GRN and now use the established tool Pando and show a UMAP-based representation of the network. The direction of the interaction is determined by the model coefficients (negative regulation: coefficient < 0, positive regulation: coefficient > 0).

Reviewer #2 (Remarks to the Author):

Melis Akgün Canan and colleagues present a compelling study exploring how two well-known transcription factors, ARX and PAX4, influence the fate decisions of alpha and beta cells using iPSC reporter lines. The authors utilize a range of advanced technologies, including transcriptome studies and stem cells, alongside conventional methods like flow cytometry and

immunofluorescence staining. Their findings provide a comprehensive overview of the mutual impact of PAX and ARX on alpha and beta differentiation. Additionally, the study demonstrates that FOXO1 inhibition and the controversial compound Artemisinins promote iPSC-derived beta cell differentiation. Overall, the study is well-designed and executed, and the discussion is clear and well-developed. However, several comments need to be addressed.

We would like to thank the reviewer for the positive comments and the constructive suggestions that helped us to improve our manuscript. We appreciate all comments and answer below to each one of the points raised.

Major Comments:
The authors use PAX4 and ARX reporter cell lines to sort cells and then run sc-RNAseq and snATACseq separately, rather than a combined multiome ATAC+Gene expression analysis. They then integrate these datasets and examine PAX4 and ARX gene patterns within the sorted data. This approach gives the impression of a closed loop in developing the topic. To reach unbiased conclusions, computational models such as RNA velocity and trajectory analysis should be conducted in parallel to validate the findings.

We agree that this is an important point and we apologize that it was not properly explained in the first version of the manuscript, since we performed single-nuclei multiome analysis (i.e. paired sn-RNAseq and snATACseq) on the sorted cells using 10X Genomics' Single Cell Multiome ATAC and gene-expression kit. To improve readability the related text of both Results and Methods sections have been edited. RNA velocity and trajectory analysis were carried out, however without achieving meaningful results. We have tried several different methods for RNA-velocity and pseudotime computation, but the results were inconsistent and did not recapitulate the anticipated developmental trajectories. We therefore did not include these results in the manuscript but provide them herein.

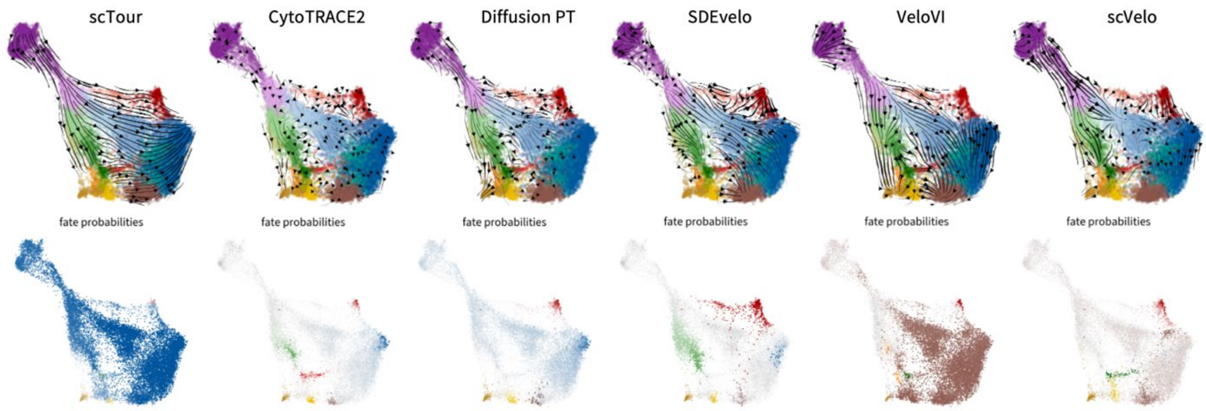


Minor Comments:
1. Lines 112-116: We notice differences in the UMAP shape and labeling in this manuscript compared to the original manuscript, ref 37. The authors should clearly explain how they recapitulated the reference data, including whether they obtained raw FASTQs, how they processed the data, and how they performed annotation and clustering.

We thank the reviewer for pointing this out. The data, originally published in ref. 37, has been reanalyzed in our lab by Yang *et al.* (ref 38). Here we use the UMAP representation and annotation from our reanalysis. We have added a reference to the preprint and apologize that we have not stated this in the first version of the manuscript.

2. Lines 199-200: Which algorithm was used for integrating the two datasets? Was it weighted nearest neighbor or another method? It appears to be non-multiomic, donor-matched separate scRNA/snATAC sequencing. If both sequencing types influenced the clustering, how were weights assigned?

We thank the reviewer for raising this point. We used 10X Genomics' Single Cell Multiome ATAC and gene-expression kit to generate paired snRNA-seq and snATAC-seq data. Both modalities were separately integrated with scANVI (gene expression) and PoissonATAC (ATAC) to remove batch effects. Subsequently, the different modalities were integrated using WNN as implemented in Muon to generate a knn network informed by both modalities. We have updated the Methods section and Figures to make this more clear.

3. Supplementary Figure 3C: It is unclear how alpha vs. alpha progenitors and beta vs. beta progenitors were divided. The dots patterns were quite similar within each subcluster.

We thank the reviewer for the comment. During this revision, we revised several steps of the multiome analysis, including the cell type annotation. In contrast to the previous version, we opted for a broader annotation scheme, where we do not discriminate between subtypes of alpha and beta cell progenitors. In general, we annotated more mature alpha and beta cells by the expression of their respective hormone, while their progenitors were labeled based on the presence of lineage and progenitor specific factors (*FEV, PAX4, NEUROG3* and *ARX, NEUROG3*) together with lower expression of the respective hormone. We have updated the Suppl. Tabe 1 to better explain how cells were annotated.

4. Lines 279-280: How did you determine the putative ARX binding site in its own gene and promote transcription by simply examining peak coverage? Please elaborate on the method or reasoning.

We thank the reviewer for the question. We propose that ARX might regulate its own expression based on motif matching and correlation analysis of chromatin accessibility with gene expression. For this, we used the Signac functions AddMotifs and LinkPeaks. Within the *ARX* locus, we found several open chromatin regions harboring an ARX motif that also show significant correlation with *ARX* gene expression. Due to the coincidence of a motif match and correlated ATAC – Gene expression signals, we assume that these sites are bound by ARX and implicated in the regulation of *ARX* gene.

5. Methods 7.4: Which tool was used to visualize the calculated GRN after motif identification?

The networks are now visualized using a modified version of Pando's `plot_network_graph` function which is based on ggraph. We added this information to the Methods section.

6. Lines 208-282 and Figure 4B: How was this chart created? Please provide details in the Methods section.

We thank the reviewer for this question. The GRN now included in the manuscript was infer using Pando, for visualization we used a modified version of Pando's plotting function (see above). We updated the Method section.
The previous version of the GRN was based on correlation between TFs (circular nodes) and ATAC peaks (triangular nodes), where correlations between TF/target genes and peaks were visualized by light-colored edges and correlations between peaks and target genes with darker colors. Positive and negative correlations were shown in blue and red respectively. The previous plot was also generated with ggraph.

7. Lines 287-299 and Fig. 4B to E: In Fig4b, FOXO1didn't have direct or indirect interaction with PAX4. How does FOXO1 inhibition support the working model in Fig 4E? This point requires clarification.

We thank the reviewer for this comment. The reviewer is correct, FOXO1 did not have any interaction with PAX4. Since *FOXO1* is more expressed in α progenitors, we reason that inhibiting its function we could manipulate α versus β cell fate decision. Biological validation of this hypothesis, as already stated in our responses to reviewer one, revealed no differences in the number of endocrine positive cells upon FOXO1 inhibition. For this reason, we remove this treatment from the main figures and show the data only here (see responses to reviewer 1).

8. Figure 5A/B/C: Were these figures generated from reporter-based sorted cells? Did this multiome data project on the data in Figure 2? The cell population percentages in the control were quite similar to the unsorted data in Figure 2C. What are the PAX4 and ARX expression levels in Figure 5B? Do these two show the similar patterns like in Fig2B?

We agree that this is important information, and we apologize that it was not properly explained in the first version of the manuscript. All multiome data sets in this study were generated from the same experiment comparing artemether with an untreated control. The untreated cells were used to generate 4 separate multiome samples, i.e. Unsorted (Control), ARX-CFP$^+$, ARX-CFP$^+$/PAX4-mCherry$^+$, PAX4-mCherry$^+$. The Unsorted sample served multiple purposes: as control for artemether treatment, as unbiased sample reflecting the actual cell type composition before sorting, and to include non-endocrine cells that are negative for both reporters. All 5 samples were preprocessed and annotated together.

Expression patterns of *ARX* and *PAX4* are similar between artemether treated cells and the control. Due to the increased fraction of endocrine progenitor cells upon artemether treatment, we observe an increased amount of *ARX*⁺ and *PAX4*⁺ cells. In addition, *ARX* expression is elevated in alpha progenitor cells.

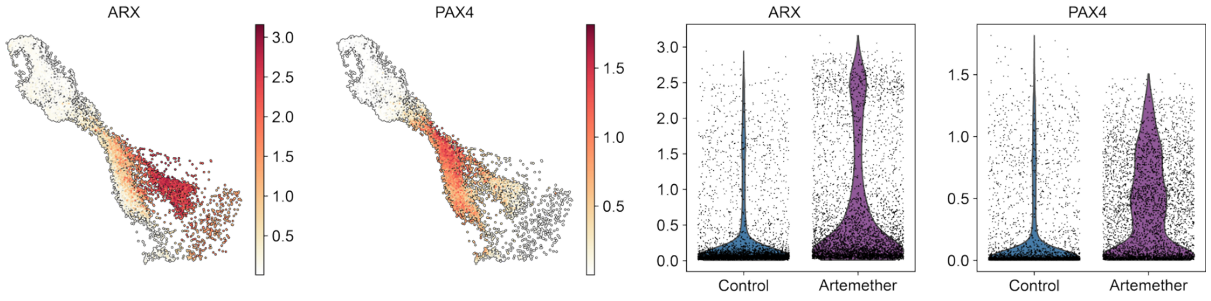


We updated Suppl. Figure 7.

9. Figure 5G: The gating is different between DMSO and Artemether.

We apologize for the mistake; the gating has been modified to be the same between both samples in the revised manuscript.

10. Methods 7.3: Specify the hyperparameters used for each algorithm, such as mitochondrial gene fraction cut-off, minimum number of genes, TSS enrichment, or nucleosome signals, et al. Additionally, it seems multiple doublet detection algorithms were used and it seems excessive compared to other studies. Could you explain the rationale behind this choice?

We agree that this is important information and have therefore included the QC parameters in Suppl. Table 1.
Regarding the doublet detection procedure, we generally find that none of the methods provide satisfactory results on their own. The rationale behind our approach is that combining the results from multiple doublet detection methods into a majority vote improves the doublet classification. A similar approach is used by Demuxafy (https://genomebiology.biomedcentral.com/articles/10.1186/s13059-024-03224-8). Moreover, we find that training a scANVI model on data containing doublets, followed by iterative steps of fine clustering and exclusion of doublet-enriched clusters, further improved the removal of doublets (see Methods for more details).

11. Methods 7.3 (GRN Inference): Validation of inferred networks using independent datasets is crucial for building a reliable GRN. The authors should provide validation using external datasets.

We thank the reviewer for raising this point and we agree that a validated GRN inference method is important. Since we lack an external dataset to validate the correlation-based GRN in the previous version of the manuscript, we decided to apply a published and widely used method (Pando) instead. We updated Figures, Results and Methods sections accordingly.

12. Line 601: Specify the nature of the internal reference dataset. What type of data does it include, how many cells, and was it previously published? Since annotation and labeling are

critical aspects of scRNA analysis, it would be beneficial to include UMAP and gene expression visuals of your reference in the supplemental data.

We agree that the use of unpublished data is not ideal and thank the reviewer for pointing this out. We have revised the code for the multiome analysis substantially during this revision. As a part of this revision, we have removed the dependency on our internal reference dataset used for initial labels provided to the integration methods. We now solely rely on marker genes for this initial annotation step (see Methods for more details).

Reviewer #3 (Remarks to the Author):

Key results

Pluripotent Stem cell-derived pancreatic islets do not perfectly mimic human or mouse islets in vivo, partially due to the reduced differentiation efficiency from the endocrine progenitor stage to the beta-cell stage. To improve our understanding of the mechanisms of endocrine cell specification during human pancreas development, Canan et al. investigate whether the classic model that describes ARX and PAX4 stochastic expression patterns in mice is conserved in human pancreas development. They generate a double reporter human induced pluripotent stem cell (iPSC) line for both ARX and PAX4 (ARXCFP/CFP; PAX4mCherry/mCherry). Using different experimental strategies, including single-cell multi-omic analysis, the authors conclude that ARX and PAX4 expression does not overlap in progenitor cells, but that PAX4 is co-expressed with NGN3+ endocrine progenitor and expressed in pre-beta and beta cells. In NGN3+/PAX4+ progenitors that develop into pre-alpha cells, PAX4 is suggested to (indirectly) activate ARX expression. From multi-omic datasets, the authors predict a gene-regulatory network (GRN) in which ARX and PAX4 do not directly interact with each other and identify FOXO1 as being regulated by ARX. Finally, the authors test the effect of artemether on alpha vs beta specification in their iPSC double tracer model and find some effects on proliferation and segregation into different endocrine fates. To summarise, the authors employed a novel iPSC model with dual reporters that allowed them to study the expression and dynamics of ARX and Pax4 and led them to propose a new working model for ARX and PAX4 expression patterns and function in human pancreas development.

Validity

-Overall, the authors employed appropriate techniques to address their research questions, the data seem valid, and the authors' interpretation of their work is appropriate.

We thank the reviewer for the positive comment.

-One of the main claims of the manuscript is that PAX4 and ARX expression do not overlap in cells. However, it is possible (but difficult to tell from the graphs alone) that Figures 1B, 2E and 3C show double positive (PAX4+ / ARX+) populations. Additionally, the authors find cells that are double positive for the fluorescent tags CFP (for ARX) and mCherry (for PAX4), but they argue that mCherry is still detected for a while after PAX4 abasexpression. Although possible, it is difficult to prove that these ‘fluorescent’ double-positives are not ‘true’ double positives.

We thank the reviewer for raising these important points. We analyzed the expression patterns of ARX and PAX4 in more detail to evaluate the extend of *ARX*/*PAX4* co-expression. Categorizing the cells in our dataset by the presence of *ARX* and *PAX4* transcripts (normalized counts ≥ 1), showed that both genes are expressed in some cells, mostly common progenitor and alpha/epsilon progenitor cells. However, in these cells, the expression seems to be shifted to either *PAX4* (common progenitors) or *ARX* (alpha/epsilon progenitors), with only very few cells expressing both genes at higher levels. These results have been added to Suppl. Figure 3. Due to drop-out, commonly observed in single-cell data, we cannot quantify the number of *ARX*/*PAX4* co-expressing cells precisely. However, IHC for the endogenous TFs on the parental WT cell line identified only 1% of the total cells as positive for both PAX4 and ARX at S4d3 and less than 1% at later stages (Suppl. Fig. 2D). Moreover, IHC showed that cell double positive for the fluorescent reporters lack endogenous PAX4 (Fig. 2G). This data has been added to the Results section.

-The proposed working model of ARX and PAX4 mechanism (Figure 4E) states that all ARX+ cells originate from PAX4+ cells. However, the paper shows the presence of a population of cells that are CFP-ARX+/ mCherry-PAX4- which might have never expressed PAX4. Since such cells might originate from a separate progenitor population compared to the CFP-ARX+/mCherry-PAX4+ cells, the authors should leave this option open in their model/discussion. The authors conclude (ca line 300) that in endocrine progenitors, PAX4 indirectly represses ARX and that, in turn, ARX indirectly represses PAX4. This cannot be concluded based on the shown data.

We thank the reviewer for these comments.
As noted, there is a population of ARX$^{+}$/PAX4$^{-}$ cells at S4. We believe that these might be ε progenitors, as suggested in the early human pancreas atlas recently published in Ma *et al.* 2023. We updated the Discussion section accordingly in the revised manuscript.
We also agree that without ChIP-Seq or similar methods, we cannot directly prove the indirect relation of the two TFs. However, due to the lack of antibodies amenable for ARX and PAX4 pulldown, the necessary ChIP-Seq experiments are not possible. However, CHIP-qPCR performed on human small intestine organoids with overexpression of FLAG-tagged ARX and/or PAX4 showed no direct interaction between ARX and PAX4 in enteroendocrine cells (Lin et al., 2023), a closely related cell type to pancreatic endocrine cells**.** Moreover, in a recent paper (Russ-Silsby and Lee, 2025), CUT and RUN assay on a stable β cell line (EndoC-βH1) transduced with FLAG-tagged PAX4 showed no binding of PAX4 on the ARX *locus*. Within the same locus, the authors also showed no overlap between differentially expressed genes in PAX4 KO stem cells-derived EP and PAX4 accessible peaks identified in the PAX4 overexpressing EndoC-βH1. We edited the model, and the Discussion section as requested.

Significance

-The manuscript is a valuable contribution to the scientific discussion on human pancreas development and sheds new light on the differences between human vs murine endocrinogenesis.

We thank the reviewer for the positive comment.

-The authors introduce an interesting iPSC line that will allow for further research into the cellular mechanisms and lineage fate decisions.

We thank the reviewer for the positive comment.

Data and methodology

Overall, the data are of high quality and are discussed clearly in the text. However, the kinetics experiment in supplementary figure 2 raises some concerns. In this figure, the author compares protein expression dynamics of mCherry and CFP fluorophores to endogenous PAX4 and ARX expression and concludes that mCherry is expressed for longer than PAX4, while CFP more faithfully represents ARX expression. To prove this, the author quantifies immunofluorescent stainings for ARX and PAX4 in an unedited, wild-type hiPSC line, and compares it to the quantification of CFP and mCherry from the other, CRISPR-engineered line. Since differentiation experiments can have some level of variability, and CRISPR engineering can partially impact cell identity and differentiation capacity, the author is advised to quantify the expression of PAX4 and ARX directly on the engineered hiPSC line.

We thank the reviewer for the suggestion. We quantified endogenous *PAX4* and *ARX* mRNA and proteins levels by qPCR, WB, and immunofluorescence on both the reporter and parental WT hiPSC lines (Suppl Fig. 2), and updated Results and Discussion sections accordingly.

Quantification of the immunofluorescence stainings throughout the manuscript will strengthen the quality of the data.

We thank the reviewer for the constructive suggestion, quantification of immunofluorescent staining has been performed and added throughout the manuscript (Suppl. Fig. 2, Fig. 3, Suppl. Fig. 5, Suppl. Fig. 7).

Analytical approach

The statistical analyses are conducted thoroughly and accurately.

We thank the reviewer for the positive comment.

Suggested improvements

-Figure 2E: please show the percentage of cells with NGN3, ARX and/or PAX4 expression. Even though the eagle-eyed view shows distinct populations, it is hard to tell whether there are smaller double/triple positive populations.

We thank the reviewer for raising this point. Due to drop-out effects, it is unfortunately not possible to accurately quantify percentages of cells expressing a given gene based on scRNA-seq data. As an approximation, we classified cells based on non-zero normalized counts for *ARX* and *PAX4* (see above) and plot the imputed expression against each other to visualize expression

patterns better. We included plots showing *ARX* vs *PAX4*, *ARX* vs *NEUROG3* and *PAX4* vs *NEUROG3* in Suppl Figure 3.

-Supplementary figure 2: The authors measured endogenous protein levels of ARX and PAX4 to map their expression patterns. This expression pattern is key for their interpretation of the measurements of the fluorescent tags and consequently for their proposed mechanism. However, the authors do not show the endogenous proteins in the mutant line even though the gene and protein patterns of ARX and PAX4 could differ between the wild-type line and the mutant line. Additionally, figure S2C is strangely plotted, and the time points are unclear. Finally, the replicate number is too low (n=2 or more). Please add additional information and data to make this figure more robust.

We thank the reviewer for the constructive suggestion. The endogenous levels of PAX4, ARX, and their reporters have been quantified by qPCR, WB and immunofluorescence on both the reporter and parental WT hiPSC lines (Suppl. Fig. 2). We also increased the biological replicates of our analysis (Fig. 1E; Suppl. Fig 2A).

-What are the levels of ARX (protein and mRNA), CFP (protein and mRNA), PAX4 (protein and mRNA) and mCherry (protein and mRNA) in the double reporter iPSC line in the different stages of differentiation (from S3 to end of S6). How does the expression of ARX and PAX4 (protein and mRNA) in the double reporter iPSC line compare with the expression of those in the wild-type line?

This is a very interesting question; we thank the reviewer for raising this point. We added RNA and protein expressions analysis of ARX, PAX4, and respective fluorophores to the Results sections of the revised manuscript (Suppl. Fig. 2). We stop our analysis at S5d7 due to our focus on endocrine induction and α versus β cell fate decision, both of which mainly occurred at S5. In addition, we found a strong preference of our cell line to differentiate more prominently into α cells (Fig. 5G), probably due to the high expression of ARX expression through differentiation. For this reason, we decide to use their parental wildtype line for analysis at S6 for various treatments.

-Figure 3C: as previously mentioned, it is difficult to tell whether ARX+/PAX4+ cells are present. Please add a quantification of the immunofluorescent staining and consider flow cytometry for ARX and PAX4 expression analysis after reaggregation. This could provide valuable additional information.

We thank the reviewer for the constructive suggestion. Due to lack of antibodies against ARX and PAX4 working for flow cytometry, we performed and quantified IHC for endogenous PAX4 and ARX on samples sorted at S5d4 and then cultured until S5d7. As shown in Fig. 3E, we could detect, on average, less than 1% double positive cells for both ARX and PAX4. The same was true for samples sorted at S5d7 and cultured for two weeks until S6d14 (Suppl. Fig. 5C).

-Figure 4A: The figure is difficult to read (e.g. PAX4 on the PAX4 locus on the left corner of the graph). Further clarification in the graph such as highlighting the PAX4 locus or more details in the text could improve the reader's comprehension.

We thank the reviewer for the constructive suggestion, we updated Fig. 4A, added Suppl. Fig. 6 and updated the Results section in the revised manuscript.

-Figure 6: Since Artemether was shown to increase the proportion of INS+ beta cells and reduce GCG+ alpha cells at the end of the differentiation protocol, it is recommended to add the flow cytometry analysis of dCaspase3 and Ki67 at the end of Stage 6 too.

In compliance with the reviewer's request, we performed cell death and proliferation analysis at S6d14 upon artemether treatment. In line with its protective effect against apoptosis at S5d7, continuous artemether treatment until S6d14 resulted into decreased cell death and unchanged proliferation. These results have been added to Fig. 6 and the Results section of the revised manuscript.

To make a stronger and conclusive claim on the newly described PAX4/ARX model, a more refined experimental design that allows performing lineage tracing on the endocrine progenitors would be required (for example – lentiviral cell tagging and multiomics at multiple time points). In this manuscript, there is no way to tell whether all ARX+ cells had previously expressed PAX4. Please add a comment about the current limitations of the mCherry-PAX4/ CFP-ARX line employed in this study in the discussion.

We agree with the reviewer on this point. We edited the Discussion section of the new version of the manuscript accordingly.

Clarity and context

-The manuscript is very well written, and the data is very well structured.

We thank the reviewer for the positive comment.

-Figure 4E (working model) could be more detailed with more space for established and putative mechanisms.

We agree with the reviewer and added more details to our working model.

References

-The authors referenced key past works and placed their data in context.

We thank the reviewer for the positive comment.

**REVIEWER COMMENTS**

Reviewer #1 (Remarks to the Author):

The authors have improved the manuscript, which now provides convincing evidence that ARX and PAX4 are only sometimes co-expressed during human pancreatic endocrinogenesis, both in fetal pancreas and hiPSC-differentiated cells. Their extensive multiomics analyses suggests that ARX and PAX4 regulation is indirect. In addition, in vitro potency assays using purified cell populations show that PAX4+ give rise to beta cells, and ARX+ or ARX+PAX4+ fluorescent cells preferentially gave rise to alpha cells. They also removed the FOXO1 inhibitor data, and include ReST inhibitor studies which strengthen the study. The GRN analysis also provides many possible upstream regulators of endocrine differentiation genes.

We would like to thank the reviewer for the time taken to review our revised manuscript. We appreciate all comments and, following the advise of the editor, we answer below to the points raised.

A central proposal of the study is that ARX-PAX4 regulation is not direct. This may be correct, but the evidence is still largely suggestive . The chromatin IP experiments would be needed to prove this point. The authors mention the limitations posed by antibody availability, but the discussion of negative ChIP results in other organs or cell types is not, in itself, compelling, as there could be direct repressor effects during transient stages of endocrinogenesis—an effect that would not necessarily be captured in gut organoids or beta cell lines. In the absence of direct evidence, it seems premature to claim that the data establish a fundamentally new differentiation model. That said, the single-cell analyses appear robust and provide a strong foundation for future experiments aimed at identifying the direct transcriptional regulators of ARX and PAX4.

We agree with the reviewer that there could be direct repressor effects during transient stages of endocrinogenesis, however due to the lack of antibodies amenable for ARX and PAX4 pulldown, the definitive ChIP-Seq experiments are not possible. During the previous round of revision, we generated custom made antibodies against PAX4, but none of these performed better in Western blot or immunofluorescence compared to the commercially available antibody that we already use. It is for these reasons that we state in the discussion that for final prove the proposed model would need to be tested combining lineage tracing, cell sorting and single cell multiomic assays at multiple time point (line 450-455). However, having said this, the obvious lack of co-expression of mRNA *in vivo* and of mRNA and protein *in vitro* make a model of mutual inhibition of ARX and PAX TFs very unlikely.

The observation that PAX4 knockout leads to elevated ARX expression raises a serious challenge to the authors' hypothesis that PAX4 induces ARX. The authors speculate that this apparent contradiction could reflect compensatory activity of other upstream regulators that promote ARX expression despite loss of PAX4. However, this explanation remains highly speculative in the absence of supporting data. If the knockout results suggest the opposite of the proposed model, additional evidence is needed to justify why the authors' interpretation is nevertheless likely to be correct.

We thank the reviewer for this comment. Due to the lack of single cell gene expression data on the mentioned PAX4 KO cell lines, we remove our previous suggestion of PAX4 *indirect* regulation on *ARX*. Unfortunately, Lau and colleagues performed bulk RNA-seq of PAX4 KO and mutant hiPSC during SC islets differentiation. This precludes us to further investigate our GRN modulation upon PAX4 KO, and the authors to discriminate between an increased *ARX* mRNA level per cell and an increase of bulk *ARX* expression due to increased $ARX^+$ cell numbers (such α or bihormonal cells) in their sample. This last hypothesis seems particularly probable due to the increased number of α cells upon *Pax4* deletion in mice (Sosa-Pineda, 1997), and of bihormonal (INS+/GCG+) cell in human PAX4 loss-of-function and dominant negative mutant iPSCs upon differentiation to SC-islets (Lau, 2023). In addition, the investigators used 3 independent PAX4 KO hiPSC, but only see a "directionally consistent, albeit nonsignificant, derepression of alpha cell genes (ARX, GCG, TTR)" mostly driven by one sample in their analysis (see heatmap below from Lau et al., 2023 – Fig. 3E).

**[Figure Redacted]**

Regarding the artemether experiments, treatment leads to a modest increase in $INS^+$ cells following sorting of $ARX\text{-}CFP^+$ or $PAX4\text{-}mCherry^+/ARX\text{-}CFP^+$ populations. The authors conclude that these data rule out α-to-β transdifferentiation because glucagon-positive cell numbers remain unchanged, which could also be due to compensatory changes. The attribution of the small increase in insulin-positive cells to residual endocrine progenitors due to incomplete sorting is plausible but not definitive. Similarly, while the use of chemical GABA agonists and antagonists suggests that GABA signaling is unlikely to mediate the observed effects, additional approaches would be required to conclusively exclude this mechanism. Importantly, no alternative mechanism is proposed to explain the changes in $INS^+$ cells. The authors

appropriately acknowledge that the effects are modest, do not impact insulin secretion, and that further optimization of artemether dosage and treatment duration would be necessary.

We thank the reviewer for their interest in artemether mechanism-of-action.
Under continuous treatment with α-to-β-promoting agents, changes in glucagon (reduction) and/or bihormonal cells (increase) are expected. The small increase in $INS^+$ cell following artemether treatment of any sorted $CFP^+$ population (ARX-$CFP^+$ or PAX4-$mCherry^+$/ARX-$CFP^+$) is not accompanied by any of these changes. In addition, due to incomplete sorting and the low level of proliferation at S6 stage (~12.5%, Fig. 6F), we find compensatory mechanisms not likely. Even if we are also curious about mechanisms-of-action of artemether, we find that further pursuing this line of research would be out of the scope of the current study.

There is another recent dual alpha vs beta reporter systems (e.g. Sci Rep 2024 Aug 27;14:19863) which would make sense to mention

We thank the reviewer for this suggestion, we mentioned this paper in the Introduction section.

Reviewer #2 (Remarks to the Author):

The revised manuscript is substantially improved, and the authors have addressed the majority of my concerns from the first review. The narrative is clearer, the GRN analysis is better contextualized, and the PAX4–ARX model is now presented with more appropriate caution. I appreciate the additional methods clarifications, improvements in figure legends, and the strengthened logic linking multiomics to the proposed regulatory framework.

We would like to thank the reviewer for the time taken to review our revised manuscript. We appreciate all comments and answer below to each of the points raised.

At this stage, several clarifications are needed:
1. Please clarify whether mCherry protein persists after endogenous PAX4 downregulation (important for interpreting double-positive reporter cells in Fig. 2 and Fig. 3E).

We agree with the reviewer that this is important information. As shown in Fig. 3D-E mCherry protein persists after endogenous PAX4 downregulation until at least S5d7. In this experiment we sorted using the mCherry reporter at S5d4 and stained for both mCherry and endogenous PAX4. At S5d7 the mCherry protein was clearly detectable in both PAX4-$mCherry^+$ and ARX-$CFP^+$/ PAX4-$mCherry^+$ clusters (Fig. 3D), while endogenous PAX4 was absent (Fig. 3E). The same results were seen at S6d14 after sorting for the reporter at S5d7 (Suppl. Fig. 5C). We clarified this point in the Results section of the revised manuscript (line 276-279).

2. Clarify the exact experimental design underlying Suppl. Fig. 2B (bulk vs sorted populations, which transcripts are measured, normalization method).

We thank the reviewer for this comment. Suppl. Fig. 2B shows a bulk qPCR analysis using commercially available TaqMan assays for *ARX*, *PAX4*, *GFP* for *CFP* and *mORANGE* for *mCherry* (Probes ID detailed in Material table 7). Gene expression levels were determined by the $2^{-\Delta\Delta CT}$

method following normalization to reference gene (GAPDH). We specified the type of analysis in the respective figure legend and Methods section.

3. In lines ~316–318, the manuscript states that "REST seems to repress PAX4… suggesting REST as a direct upstream regulator of PAX4 and endocrine induction." However, the GRN table does not indicate a strong or consistent REST→PAX4 repressive relationship, nor a REST→ARX interaction. REST instead clusters with a non-endocrine/progenitor module (HES1, ID2, HMGA1/2, SOX9), with negative associations to INSM1 and PAX6.

We thank the reviewer for this comment, and we agree that this is important information. Our GRN identified REST as an upstream repressor of the endocrine induction machinery (*FEV, INSM1, NKX2-2*) and a promoter/keeper of PP identity (ONECUT1, SOX9, HES1) in non-endocrine and pre-endocrine cells. The results of REST inhibition in our system further strengthen this hypothesis and are in line with the previous literature in pancreatic organoids, Zebrafish embryos, and *Rest* KO mice. This data highlights a previously unrecognized role of REST in preserving pancreatic cell identity while suppressing endocrine induction, and thus preventing endocrine cell fate acquisition in PP during human *in vitro* differentiation toward β cells. We updated both the Results and the Discussion sections

4. In Suppl. Fig. 7C, both INS and GCG expression appear visually reduced across conditions. The description around lines ~366–367 does not explain this pattern, and readers may find it confusing.

We thank the reviewer for this comment. We specified both in the text of both the Results section and the figure legend that the plots in Suppl. Fig. 7C represent gene expression of S5d4. We also better explained the cellular composition of SC-islets at S5d7 and S6d14 upon the two different artemether treatment schemes (line 373-375).

Reviewer #3 (Remarks to the Author):

The authors have satisfactorily addressed all the comments raised.

We would like to thank the reviewer for the time taken to review our revised manuscript and for helping us improve it with their previous suggestions and comments.
